# Dipeptidic Phosphonates: Potent Inhibitors of *Pseudomonas aeruginosa* Elastase B Showing Efficacy in a Murine Keratitis Model

**DOI:** 10.1002/advs.202411807

**Published:** 2025-02-19

**Authors:** Alexander F. Kiefer, Christian Schütz, Colya N. Englisch, Dominik Kolling, Samira Speicher, Andreas M. Kany, Roya Shafiei, Noran A. Wadood, Ahmad Aljohmani, Niklas Wirschem, Ravindra P. Jumde, Andreas Klein, Asfandyar Sikandar, Yu‐Mi Park, Gabriela Krasteva‐Christ, Daniela Yildiz, Ahmed S. Abdelsamie, Katharina Rox, Jesko Köhnke, Rolf Müller, Markus Bischoff, Jörg Haupenthal, Anna K. H. Hirsch

**Affiliations:** ^1^ Helmholtz Institute for Pharmaceutical Research Saarland (HIPS) Campus E8.1 66123 Saarbrücken Germany; ^2^ Helmholtz Centre for Infection Research (HZI) Inhoffenstraße 7 38124 Braunschweig Germany; ^3^ German Center for Infection Research (DZIF) Inhoffenstraße 7 38124 Braunschweig Germany; ^4^ PharmaScienceHub (PSH) Campus A2.3 66123 Saarbrücken Germany; ^5^ Institute for Medical Microbiology and Hygiene Saarland University Kirrbergerstraße 100 66421 Homburg/Saar Germany; ^6^ Institute for Food Chemistry Callinstraße 5 30167 Hannover Germany; ^7^ Department of Pharmacy Saarland University Campus E8.1 66123 Saarbrücken Germany; ^8^ Institute of Anatomy and Cell Biology Saarland University Kirrbergerstraße 100 66421 Homburg/Saar Germany; ^9^ Institute of Experimental and Clinical Pharmacology and Toxicology PZMS ZHMB Saarland University Kirrbergerstraße 100 66421 Homburg/Saar Germany; ^10^ Department of Chemical Biology Helmholtz Centre for Infection Research (HZI) Inhoffenstraße 7 38124 Braunschweig Germany; ^11^ Helmholtz International Lab for Anti‐Infectives Campus E8.1 66123 Saarbrücken Germany

**Keywords:** elastase B, keratitis, phosphonates, *Pseudomonas aeruginosa*

## Abstract

The ubiquitous opportunistic pathogen *Pseudomonas aeruginosa* is responsible for severe infections and notoriously known for acquiring antimicrobial resistance. Inhibiting the bacterium's extracellular elastase, LasB – a zinc‐dependent protease – presents a promising strategy to mitigate its virulence. Within this medicinal chemistry–driven hit‐to‐lead optimization campaign, a new series of highly potent dipeptidic phosphonates is designed and synthesized following a structure–based drug‐discovery approach. In vitro and in vivo evaluation reveal beneficial pharmacokinetic profiles, excellent selectivity over human off‐targets and good tolerability in murine toxicity studies. Ultimately, the scaffold presented herein demonstrates promising in vivo efficacy in a murine *Pseudomonas aeruginosa* keratitis model in combination with the antibiotic meropenem.

## Graphical abstract

A new series of highly potent dipeptidic phosphonates targeting the *Pseudomonas aeruginosa* protease LasB shows promising in vitro effects, favorable pharmacokinetics, good tolerability, and in vivo efficacy, highlighting their potential as a treatment for *Pseudomonas* keratitis when combined with meropenem.

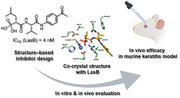

## 1 Introduction

The infectious inflammation of the cornea, known as infectious keratitis, ranks as the fifth leading cause of blindness and visual impairment worldwide.^[^ ^1^, ^2^ ^]^ Annually, it accounts for more than 1.5 million new cases of monocular blindness, emphasizing its persistent and substantial public‐health impact.^[^ ^3^ ^]^ Among the causes of corneal opacity, microbial keratitis – attributable to bacterial, fungal, and protozoal pathogens – emerges as the predominant etiology of corneal blindness in both developed and low‐ and middle‐income countries.

**Figure 1:**
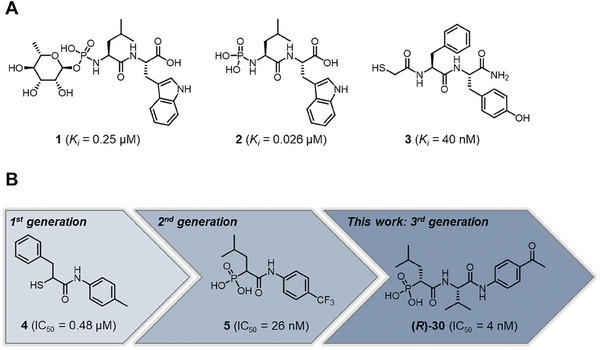
(**A**) Structures of known peptidic LasB inhibitors. Inhibitory constant *K*_i_ values for elastase LasB are given in brackets. (**B**) Fragment‐to‐hit optimization of phosphonate dipeptides via mercaptoacetamide **4** and α‐substituted phosphonic acid **5**. Half‐maximal inhibitory concentrations (IC_50_s) are given in brackets.

In addition to *Staphylococcus aureus* and *Streptococcus pneumoniae*, *Pseudomonas* *aeruginosa* is a primary bacterial pathogen associated with infections in compromised corneas (*Pseudomonas* keratitis).^[^ ^2^, ^4^ ^]^ Particularly contact lens‐associated keratitis is predominantly caused by *P. aeruginosa*.^[^ ^5^, ^6^, ^7^ ^]^ Intensive topical antibiotic therapy, utilizing fluoroquinolones (e.g., ciprofloxacin), aminoglycosides (e.g., tobramycin), or *β*‐lactam antibiotics (e.g., meropenem), remains the primary treatment approach.^[^ ^8^, ^9^ ^]^ Nevertheless, this Gram‐negative opportunistic bacterium can rapidly develop antibiotic resistance, making infections extremely difficult to treat effectively.^[^ ^10^, ^11^ ^]^ Notably, *P. aeruginosa* infections often progress from corneal perforation to severe liquefactive necrosis of the cornea, driven by complex pathogenic mechanisms involving both host and bacterial factors.^[^ ^12^, ^13^ ^]^ Here, *P. aeruginosa* proteases have been considered to be important virulence factors for invasion in acute ocular infections.^[^ ^14^, ^15^ ^]^ *P. aeruginosa* secretes different proteases, such as staphylolysin (LasA),^[^ ^16^ ^]^ aminopeptidase,^[^ ^17^ ^]^ protease IV,^[^ ^18^ ^]^ and, importantly, elastase LasB (elastase B, pseudolysin). The most abundant extracellular protease LasB, is a zinc‐dependent metalloprotease encoded by *lasB*.^[^ ^19^ ^]^ LasB can degrade collagen, mucins and other host proteins such as surfactant proteins,^[^ ^20^ ^]^ cytokines,^[^ ^21^ ^]^ and immunoglobulins, and can cause other harmful effects on various components of the immune system.^[^ ^22^, ^23^ ^]^ Immunization with LasB has been demonstrated to confer protection against *Pseudomonas* keratitis in both rabbits and mice.^[^ ^12^, ^24^, ^25^ ^]^ Additionally, studies have shown that peptidic metal‐chelating elastase inhibitors (**1**–**3**)^[^ ^26^, ^27^, ^28^ ^]^ can significantly reduce corneal melting in an experimental rabbit model of *Pseudomonas* keratitis (**Figure** **1**A).^[^ ^29^ ^]^ Although effective, advancing this approach to a more sophisticated level by slowing the infection to allow sufficient time for antibiotic treatment to preserve vision remains a significant challenge.

In this study, we describe the systematic hit‐to‐lead optimization process, which integrated a peptidic backbone with α‐substituted phosphonates as a zinc‐binding group. This strategy was based on a LasB inhibitor scaffold reported previously by our group that is efficacious after inhalative administration.^[^ ^30^ ^]^ In this work, we improved the in vitro activity, resulting in highly potent compounds that inhibit LasB with single‐digit nanomolar potency. The binding modes of three phosphonic acid‐containing dipeptides to LasB were elucidated through X‐ray co‐crystal structures. Additionally, in vitro profiling was performed to characterize our frontrunners, followed by in vivo toxicity studies. Finally, an in vivo proof‐of‐concept study demonstrated the efficacy of adjunctive treatment with meropenem in a murine *Pseudomonas* keratitis model.

## 2 Results and Discussion

### 2.1 Structure–Activity Relationship of Phosphonic Acid‐Containing Dipeptides

Our group recently reported the design, synthesis, and biological evaluation of small‐molecule inhibitors targeting *P. aeruginosa* elastase B.^[^ ^30^, ^31^, ^32^, ^33^, ^34^ ^]^ Optimization of the initial thiol‐based hit **4** involved introducing an isobutyl side chain at the α‐position and substituting the zinc‐binding motif with a phosphonic acid group (1^st^ and 2^nd^ generation). This strategic modification led to the development of phosphonate **5**, which exhibited an 18‐fold enhancement in half‐maximal inhibitory concentration (IC_50_) compared to the original hit. Building on these findings, the modifications implemented in the 3^rd^ generation of compounds was based on two main objectives. First, based on the co‐crystal structures of previously reported 1^st^ and 2^nd^ generation LasB inhibitors, we expected an improvement exploiting a possible growth vector. Second, considering LasB's intrinsic protease activity, we sought to incorporate structural motifs resembling the peptidic substrate found in compounds such as phosphoramidon and peptide‐based metal‐chelating elastase inhibitors (Figure 1B).^[^ ^19^ ^]^

First, we systematically incorporated amino acid moieties adjacent to the leucine‐derived phosphonate, as detailed in **Table** **1**. Starting with glycine, we synthesized the dipeptide **7**, which exhibited a significant decrease in activity compared to phosphonate **5**. Conversely, the incorporation of a single methyl group by introducing alanine in **8** led to a remarkable 28‐fold increase in activity. Increasing bulkiness in the valine‐derived inhibitor **9** was the first compound in the series to achieve a single‐digit nanomolar IC_50_ value. The corresponding (*R*)‐valine analogue **10** also demonstrated substantial activity, though slightly reduced compared to **9**. Incorporation of the beta‐amino acid (*S*)‐*β*‐Val in **11** caused a significant decrease in LasB inhibition, likely due to deviations from the natural peptide structure. In contrast, cyclopropylglycine (**12**), cyclobutylglycine (**13**), cyclohexylglycine (**14**) and isoleucine (**15**) derivatives exhibited similar activity to **9**. Notably, replacing leucine with cyclopropyl alanine (**16**) resulted in more than a two‐fold increase in activity, potentially due to entropic effects. The introduction of *O*‐methyl‐protected serine facilitated the separation of diastereomers **(*R*)‐18** and **(*S*)‐18** via preparative high‐performance liquid chromatography (HPLC). The (*R*)‐configured diastereomer **(*R*)‐18** exhibited twice the potency of **(*S*)‐18**, aligning with our recent findings on α‐substituted phosphonic acids^[^ ^30^ ^]^ and mercaptoacetamide^[^ ^32^ ^]^ derivatives. Peptides containing bulkier residues such as cyclohexyl alanine (**19**) and pyran‐4‐yl alanine (**20**) resulted in an over 48‐fold loss in inhibition, likely due to steric repulsion. Additionally, the incorporation of proline into the backbone (**21**) led to a substantial drop in activity, likely due to significant conformational changes. Introduction of aromatic amino acids such as phenylglycine (**22**), phenylalanine (**23**), 2‐pyridyl‐alanine (**(*R*)‐24**/**(*S*)‐24**) and 3‐pyridyl‐alanine (**(*R*)‐25**/**(*S*)‐25**) resulted in significantly less potent derivatives. The diastereomeric mixtures of the latter two compounds could be resolved by preparative HPLC, following the same trend as previously described.

**Table 1:** LasB inhibition of α‐isobutyl phosphonates with varied amino acids in dipeptide backbone.

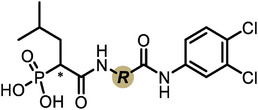					
Cmpd	*R*	IC_50_ [nM]	Cmpd	*R*	IC_50_ [nM]
**7**	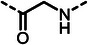	1756 ± 30	(*R*)‐**18**	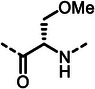	86.7 ± 3.6
**8**	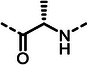	61.7 ± 1.6	(*S*)‐**18**		197 ± 5.4
**9**	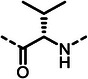	5.1 ± 0.2	**19**	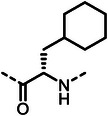	242 ± 21
**10**	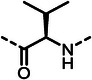	11.3 ± 0.4	**20**	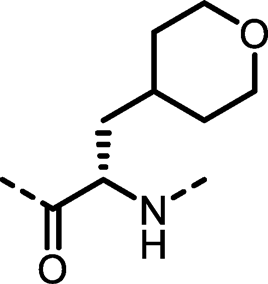	379 ± 8
**11**	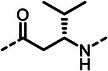	2715 ± 55	**21**	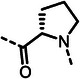	2539 ± 69
**12**	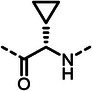	28.8 ± 0.8	**22**	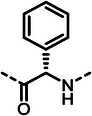	99.6 ± 2.5
**13**	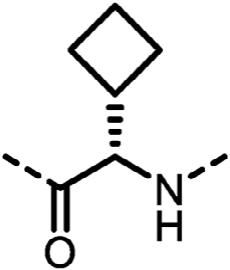	11.9 ± 0.3	**23**	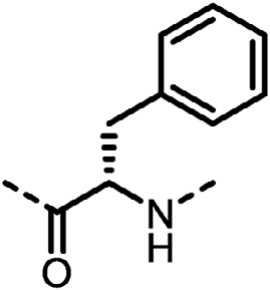	426 ± 48
**14**	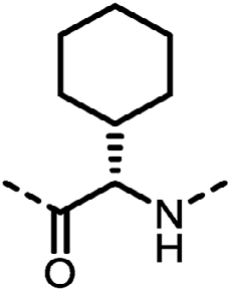	12.4 ± 0.3	(*R*)‐**24**	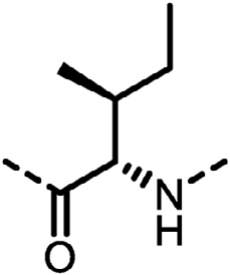	416 ± 23
**15**	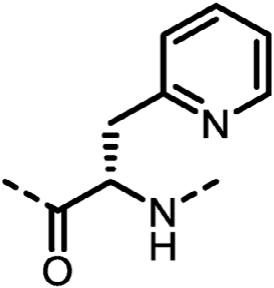	13.1 ±0.5	(*S*)‐**24**		1360 ± 50
**16**	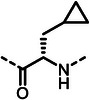	27.1 ± 0.9	(*R*)‐**25**	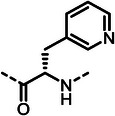	574 ± 16
**17**	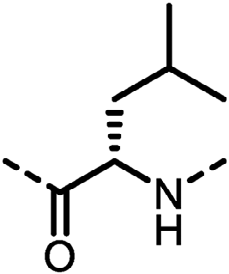	59.4 ± 1.7	(*S*)‐**25**		1500 ± 60

With dipeptide **9** in hand, we proceeded to investigate different substitution patterns of the aryl core, incorporating both electron‐withdrawing and ‐donating substituents (**Table** **2**). Drawing on our previous research on α‐substituted phosphonic acid derivatives, we hypothesized that substitution at the *para*‐position would be more favorable for LasB inhibition compared to *ortho* and *meta* positions. To test this hypothesis in the context of the dipeptide class, we first evaluated the mono‐chlorine derivatives **27** and **28**. Our results confirmed that single‐digit nanomolar potency was attainable only when the substituent was introduced at the *para*‐position. The substitution of the chlorine atom with a trifluoromethyl‐group in **29** resulted in a slight decrease in inhibitory activity. Notably, the introduction of an acetyl group allowed for the separation of diastereomers via preparative HPLC. The (*R*)‐configured diastereomer, **(*R*)‐30**, exhibited significantly greater efficacy compared to its (*S*)‐configured counterpart, **(*S*)‐30**. Furthermore, the activity of **(*R*)‐30** was comparable to that of **9**. Modification with electron‐donating groups, such as methyl (**32**) or methoxy (**33**), resulted in only a slight reduction in activity. Surprisingly, the incorporation of an isopropoxy substituent (**34**) significantly decreased the activity by a factor of five. Conversely, the introduction of a phenoxy substituent (**35**) restored the activity. The separated imidazoyl‐group‐containing diastereomers **(*R*)‐36** and **(*S*)‐36** exhibited a similar trend to the acetyl‐containing dipeptides, with the (*R*)‐configured inhibitor being more active. Lastly, the incorporation of a bulky morpholino residue (**37**) resulted in a 16‐fold decrease in activity compared to **9**.

**Table 2:** LasB inhibition of α‐isobutyl phosphonate containing dipeptides with varied aryl groups.

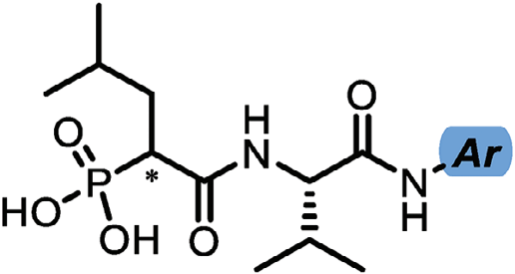					
Cmpd	*Ar*	IC_50_ [nM]	Cmpd	*Ar*	IC_50_ [nM]
**27**	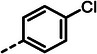	9.0 ± 0.4	**32**	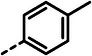	12.9 ± 0.4
**28**	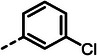	282 ± 13	**33**	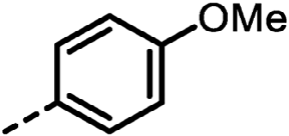	19.9 ± 0.8
**29**	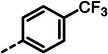	15.2 ± 0.3	**34**	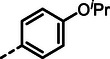	56.7 ± 2.0
**30**	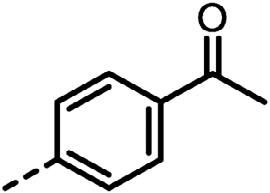	5.0 ± 0.2	**35**	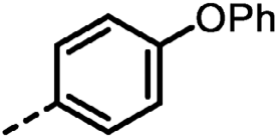	8.0 ± 0.3
**(*R*)‐30**		3.7 ± 0.1	**(*R*)‐36**	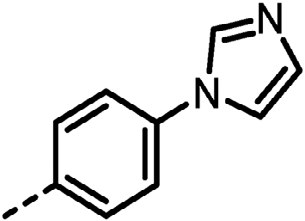	10.9 ± 0.3
**(*S*)‐30**		129 ± 4.0	**(*S*)‐36**		417 ± 14
**31**	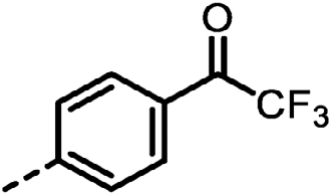	21.7 ± 0.6	**37**	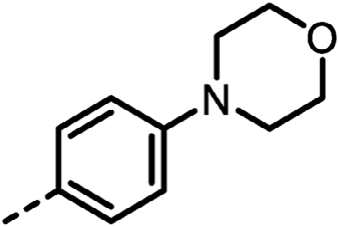	83.7 ± 2.1

In conclusion, we successfully identified and optimized a novel dipeptide scaffold, achieving nanomolar activity. We selected the most potent LasB inhibitors, **9**, **30**, **(*R*)‐30**, being selected for further in‐depth evaluation.

### 2.2 Co‐Crystallization Studies

**Figure 2:**
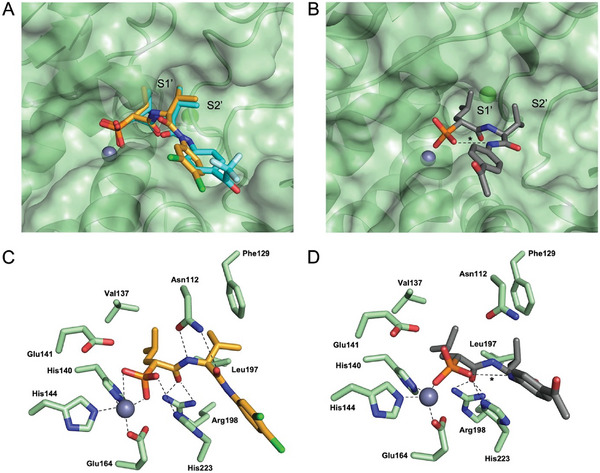
Crystal structure of LasB in complex with **9**, **31** and **30** (PDB codes: 9FQD, 9FQY, 8RIB). (**A**) Superposition and surface representation of the LasB (green) co‐crystal structures in complex with **9** (orange) and **31** (cyan). (**B**) Surface representation of LasB (green) in complex with **30** (gray). The intramolecular hydrogen bond is highlighted by an asterisk. (**C**) Schematic 2D representation of interactions between **9** and LasB. The catalytically active Zn^2+^ cation coordinated by His140, His144, Glu164, and the phosphonate is shown as a gray sphere. (**D**) Schematic 2D representation of interactions between **30** and LasB. The aryl ring of **30** is significantly shifted in the co‐crystal structure, possibly because of the intramolecular hydrogen bond between a phosphonate oxygen and the second amide nitrogen atom (highlighted by an asterisk). The dimethyl moiety of the valine is rotated by 90° compared to the structure of LasB in complex with derivative **9**.

The dipeptide compounds designed in this study show, for the first time, activity in the single‐digit nanomolar range compared to previously published phosphonates.^[^ ^30^ ^]^ This is probably due to the peptidic valine linker between the isobutyl and benzyl ring decorated with electron‐withdrawing groups. To gain further insights into the binding mode of the optimized compounds and explain the increased affinity, we determined the co‐crystal structures of compounds **9**, **30**, and **31** (**Figure** **2**; Table S1, Supporting Information). Since the dipeptide compounds share the phosphonate zinc‐binding group, the *iso*‐butyl moiety, and the amide with the compound in a previously published structure (PDB ID 8CC4), key interactions were conserved. These include hydrogen bonds to the side chains of His223, Glu141, and Asn112 and the bidentate hydrogen bond to Arg198 (Figure S1, Supporting Information). However, in contrast to the α‐aryl‐substituted phosphonates, the peptidic valine linker of the optimized compounds occupies the lipophilic S2' pocket of LasB and engages in a hydrophobic interaction with Leu197 (Figure 2A,C). The increased binding affinity to LasB can be rationalized primarily by the introduction of the additional amide bond of the compound series presented here and the substitution pattern of the adjacent aryl ring. For example, the carbonyl oxygen atom of the keto‐group in compound **31** forms additional hydrogen bonds with Arg208 and His224, which results in activity in the low two‐digit nanomolar range (Figures S1,S2, Supporting Information). In the co‐crystal structure of LasB with **9**, the side chain of Asn112 forms a hydrogen bond with the carbonyl oxygen atom of the dipeptide motif as well as an additional hydrogen bond with the amide nitrogen atom (Figure 2B–D). The extremely efficient bidentate chelation of zinc by the phosphonate, as well as a bidentate hydrogen bond with the side chain of the catalytically active His223 could explain the increased affinity of compound **9**. Compared to the other compounds, **30** (and the more active diastereomer **(*R*)‐30**) exhibited the most potent in vitro activity. The significant difference in affinity for the two diastereomers of **30** can be explained by the occupancy of the *iso*‐butyl moiety in the S1’ pocket, which enables a more efficient hydrophobic interaction for **(*R*)‐30**.

Extremely striking regarding the binding mode of **30** is the coordination of zinc by the phosphonate oxygen atom: In contrast to the other two compounds (**9** and **31**), the chelation we observed was not bidentate. Instead, only one of the phosphonate oxygen atoms engages the Zn^2+^ cation. This enables the formation of an intramolecular hydrogen bond between a phosphonate oxygen atom and the second amide NH (starting at the phosphonate) (Figure 2D). The change in ligand orientation leads to the disruption of the bidentate hydrogen‐bonding interaction between LasB Asn112 and **30**, which appears to be more than compensated for by the intermolecular interaction and resulting compound conformation. The reduction of available conformations for **30** may lead to decreased entropic penalty upon binding to LasB, resulting in a significantly different binding mode compared to **9** and **31** (Figure S3–S6, Supporting Information).

### 2.3 Pharmacokinetic Studies

Regarding their in vitro absorption–distribution–metabolism–excretion–toxicity (ADMET) properties, frontrunners **9** and **30** as well as additional representative compounds **13**, **14, (*R*)‐36** have demonstrated the same excellent profile observed with the previously reported monoaryl phosphonates.^[^ ^30^ ^]^ The tested inhibitors exhibited high solubility, good metabolic and plasma stability (Tables **3** and Figure S2, Supporting Information). For **30**, these properties were validated across multiple species with excellent microsomal and plasma stability in mouse, rat, and minipig fractions (Table S3, Supporting Information). As expected, the high stability could also be confirmed in selected assays for the more active diastereomer **(*R*)‐30** (Table S2, Supporting Information). Additionally, we evaluated the Calu‐3 permeability of **9** and **30**. As anticipated for these polar structures, both compounds demonstrated low permeability, with values of 0.71 ± 0.24 and 0.86 ± 0.39 × 10⁻⁶ cm ^−1^s, respectively (Figure S7, Supporting Information). These are also expected considering the low lipophilicity, as also characterized by LogD_7.4_. As ADMET data were promising, we conducted a pharmacokinetic study with 30 mg/kg **30** via subcutaneous administration. We observed a good exposure in plasma and also a good exposure in epithelial lining fluid (ELF) and lung tissue. However, ELF exposure was slightly lower compared to plasma with an ELF/plasma ration of ≈0.38. Additionally, the compound distributed well in other tissues (Figure S8, Table S4 and Figure S5, Supporting Information). No accumulation into any tissue investigated that may cause unwanted toxicity was observed.

**Table 3:** In vitro ADME results of selected LasB inhibitors. Cl_int_, intrinsic clearance; t_1/2_, half‐life.

Cmpd	Kinetic Solubility PBS pH 7.4 [µM]	Liver S9 Cl_int_ [µL/mg/min]		Plasma t_1/2_ [min]		LogD_7.4_
		Mouse	Human	Mouse	Human	
**9**	>200	<5.8	<5.8	>150	>150	0.41
**30**	>200	<5.8	<5.8	>150	>150	−0.58

### 2.4 Selectivity Toward Human Off‐Targets

Next, we investigated the activity of seven selected LasB inhibitors (**9**, **13**, **14**, **30**, **(*R*)‐30**, **31**, **(*R*)‐36**) against four human zinc‐dependent matrix metalloproteinases (MMP) 1–3 and tumor necrosis factor‐alpha converting enzyme (TACE, or a disintegrin and metalloprotease 17 (ADAM17)) as human off‐targets. All of these targets play crucial roles in physiological processes, hence selectivity over these by our compounds is desirable. Satisfyingly, at a concentration of 100 µM, none of the tested compounds exhibited more than 15% inhibitory activity against these off‐targets (Table S6, Supporting Information).

Moreover, we explored the safety profile of frontrunner **(*R*)‐30** in more detail in order to detect additional crucial off‐target interactions. For this purpose, we employed the SafetyScreen44 in vitro panel comprising selected binding, inhibition and uptake assays.^[^ ^35^ ^]^ The panel is a specialized pharmacological profiling tool that tests compounds against 44 key molecular targets, aiming to identify potential safety risks early in drug discovery. At a comparably high concentration of 100 µM (general compound concentration in these assays is 10 µM), **(*R*)‐30** displayed inhibition binding to ten targets by >50%, for four of them even by >80% (Figures S9,S10 and Table S7, Supporting Information). The latter comprise off‐targets such as the potassium channel hERG (79.4% binding inhibition), the sodium channel (91.1%), the **5**‐hydroxytryptamin transporter (5‐HTT) (84.8%) or cyclooxygenase 1 (COX1) (81.5%) (Table S7, Supporting Information).

It remains to be determined whether this compound also causes functional inhibition. Nonetheless, the overall observation is significant and warrants detailed consideration, particularly when the compound is administered systemically. Specifically, **(*R*)‐30** is well‐suited for topical administration, such as ocular application. In such cases, it can be assumed that only minimal quantities will enter the bloodstream, thereby minimizing the likelihood of adverse effects. Additionally, this observation seems to be compound‐related and not class‐specific, as we reported previously on an excellent safety profile for a monoaryl phosphonate derivative, which might be more favorable for systemic administration.^[^ ^30^ ^]^

### 2.5 Toxicity Studies

The seven dipeptides were also tested for cytotoxic effects against HepG2, HEK293, and A549 cell lines. Encouragingly, all LasB inhibitors demonstrated minimal to no impact on human cells (Table S8, Supporting Information). This is in agreement with previous findings on structurally simpler phosphonates.^[^ ^30^ ^]^ **30** was also tested in a zebrafish embryo toxicity model, which revealed no signs of toxicity up to five days post fertilization at up to 100 µM (Table S9, Supporting Information).

Motivated by these results in cell culture and zebrafish embryos, but with the partial yellow flags in the Safety Screen44 in mind, we conducted a safety study in mice with frontrunner **30**. These experiments were carried out although, as described above, topical application is preferred for the dipeptides. We aimed to generate information about the maximum tolerated dose in a representative species in order to de‐risk the scaffold for further development. For the first toxicity study, **30** was applied as a single, subcutaneous dose ranging from 25–400 mg/kg. At all doses up to 400 mg/kg, no clinical signs and no macroscopic findings were observed. **30** only showed a slight effect on food consumption and on thymus weight with a moderate increase (Tables S10,S11 and S12, Supporting Information). Based on these encouraging results, we performed a repeated–dose follow‐up study using an administration scheme with TID intravenous dosing, escalating the total dose to 300 and 600 mg/kg/day, for 3 days. For this study, we further decided to investigate the more active diastereomer **(*R*)‐30**. Again, no treatment–related mortality, nor effects on body weight or clinical signs were observed. There was no effect on food uptake either, yet slightly increased weight of the liver, spleen, and testes were found (Tables S13,S14 and S15, Supporting Information). Whether the observed differences in the minor effects on organ weight and food consumption are related to the application of the pure diastereomer versus the mixture in the two studies cannot be clearly assessed at this stage, but might be a possible explanation.

Despite these observations, the effects noted were minimal and occurred only at relatively high doses. For a topical application, as suggested above, significantly lower amounts of compound will enter the system, suggesting a safe application of these LasB inhibitors is possible.

Although we are developing antivirulence agents, which are not supposed to have antibacterial activity but only to reduce the pathogenicity of *P. aeruginosa*, we tested the activity of compounds against *P. aeruginosa* strains PA14 and PA54 and did not observe any effect on bacterial growth at 100 µM (Table S16 and Figure S11, Supporting Information). Furthermore, we did not observe an effect on biofilm formation of PAO1 using a peg lid‐based minimum biofilm eradication concentration (MBEC) model (Figure S12, Supporting Information).^[^ ^36^ ^]^

### 2.6 In Vivo Studies in a Murine Keratitis Model

To assess the therapeutic potential of our LasB inhibitors – which exhibit excellent in vitro activities with favorable in vitro ADME, low cytotoxicity and good tolerability – for treating an infection via topical administration, we decided to employ a *Pseudomonas* keratitis model in mice recently established in our group.^[^ ^37^ ^]^

As our in vivo model utilizes the *P. aeruginosa* clinical isolate PA54, the expression of *lasB* was verified as initial step. The expression in PA54 was compared to the reference strain PAO1, known for its moderate *lasB* expression, and the high‐level *lasB* producer strain PA14, respectively (Table S17 and Figure S13, Supporting Information). These experiments revealed that PA54 is characterized by *lasB* transcript rates under in vitro growth conditions in between the transcript rates produced by PAO1 and PA14, making it suitable for the administration of a LasB inhibitor.

Based on our previous findings on target engagement in a murine pneumonia model in combination with a standard‐of‐care antibiotic,^[^ ^30^ ^]^ we applied the dipeptide **(*R*)‐30** alone and in combination with the commonly used antibiotic meropenem.^[^ ^9^ ^]^ The keratitis model was carried out using aged female C57BL/6 mice, as previously published.^[^ ^37^ ^]^ For infection, 5*10^7^ colony forming units (CFUs) of PA54 were topically administered on denuded, mechanically harmed corneas. Treatments with meropenem, **(*R*)‐30**, or meropenem in combination with **(*R*)‐30** were performed every 8 h for 72 h (first treatment at 6 h post infection). Each of the in total nine 5 µL‐doses contained 1 mg/mL **(*R*)‐30** and/or 0.5 mg mL^−1^ meropenem. Sham‐treated mice received 5 µL‐doses of the vehicle (0.9% saline and 1% DMSO) at the time points indicated above, and uninfected eyes received no treatment.

**Figure 3:**
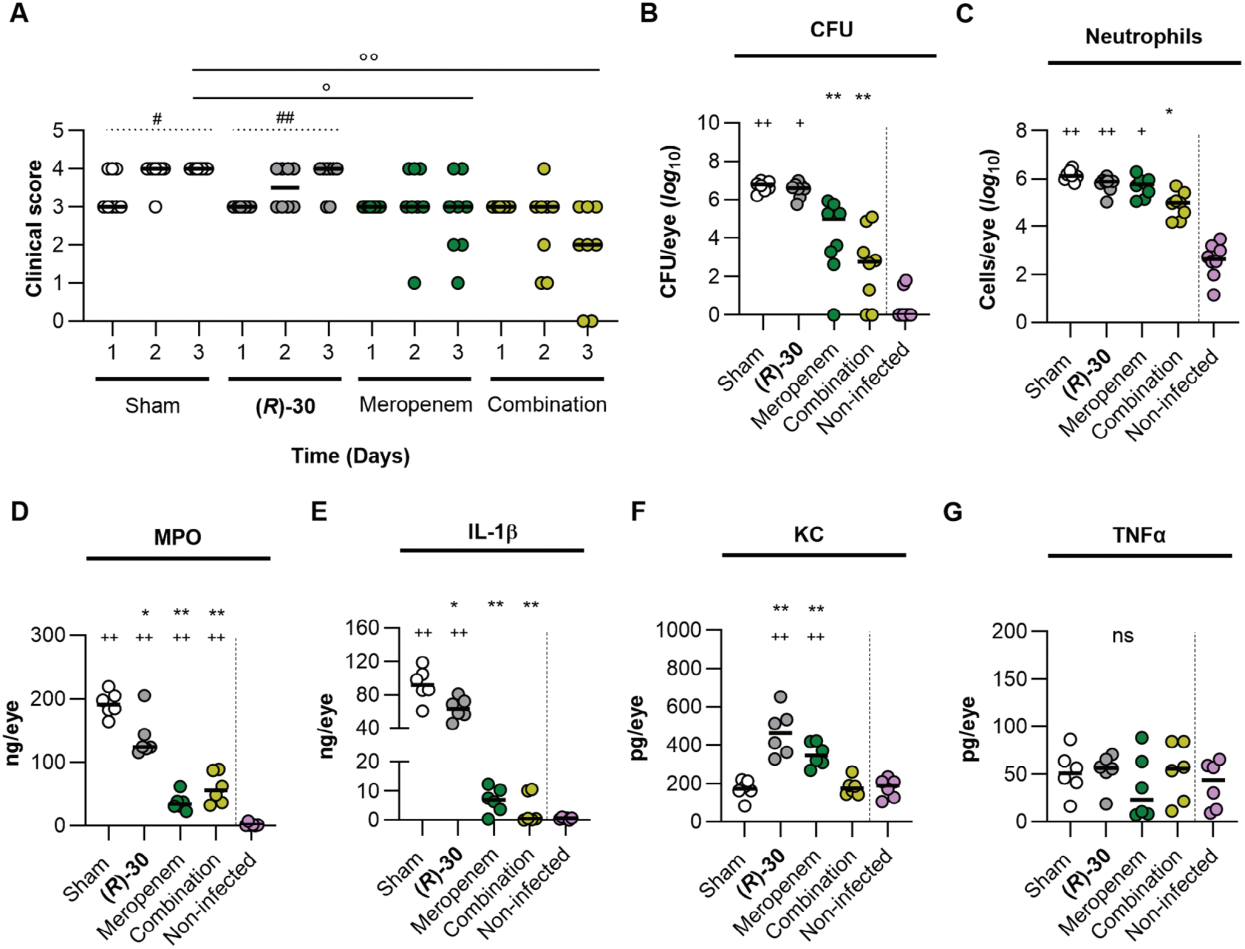
Impact of drug treatment on keratitis development in C57BL/6N mice after infection with strain PA54. (**A**): Evolution of clinical score from day 1 to day 3 (n = 8 per group). (**B**) Determination of bacterial loads in whole‐eye homogenates at 3 days post infection (n = 8 per group). (**C**) Neutrophils detected in whole‐eye homogenates at 3 days post infection (n = 6 per group). (**D‐F**) Myeloperoxidase (MPO, **D**), interleukin‐1β (IL‐1β, **E**), keratinocyte chemoattractant (KC, **F**), and tumor necrosis factor α (TNFα, **G**) contents in whole eye homogenates obtained from eyes at 3 days post infection (n = 6 per group). Data represent the values of every individual animal (symbols) and the median (horizontal line). ns, not significant; ° or # or + or *, *P* < 0.05; °° or ## or ++ or **, *P* < 0.01. + and *, Brown–Forsythe and Welch ANOVA test with Dunnett's T3 multiple comparison test. +, comparison between the mean of each group with the mean of the non‐infected control. *, comparison between the mean of each group with the mean of the sham‐treated control. °, Ordinary one‐way ANOVA with Dunnett's multiple comparison test. #, RM one‐way ANOVA test with Dunnett's multiple comparison test.

As a general measure of condition, the change in body weight of the animals was assessed and revealed no systematic changes, which indicates good tolerability in agreement with the previous safety study (Figure S14, Supporting Information). To gain first insights on the impact of our treatments on disease progression, we determined the clinical keratitis scores on a daily basis (**Figure** **3**A). While the infected eyes of all sham‐treated or only LasB inhibitor‐treated mice displayed a severe keratitis at day three post infection (i.e., a median clinical score of 4), this was not the case with the meropenem‐treated groups. When treated with the antibiotic alone, already three of the eight meropenem‐treated animals showed a decrease in keratitis severity over the study time window (median clinical score of 3 at day three post infection). This effect was more pronounced when meropenem was used in combination with **(*R*)‐30** in which 5 out of 8 eyes displayed a clinical score ≤2 at the endpoint, indicating an enhanced healing of the injured tissue (Figure 3A; median clinical score of 2).

When assessing the bacterial loads being present in the infected eyes on day 3 post infection, a comparable pattern emerged. Treatment of the infected eyes with **(*R*)‐30** alone had no clear effect on the bacterial loads in the eyes 3 days post infection. Treatment of the infected eyes with meropenem alone reduced the bacterial load in the median by 64‐fold, and this effect was even more pronounced in **(*R*)‐30** plus meropenem treated eyes (4‐*log*_10_ reduction; Figure 3B). While **(*R*)‐30** had minimal impact on the infection process when used alone, these observations suggest that combining the LasB inhibitor with the antibiotic significantly enhanced bacterial infection clearance, surpassing the effectiveness of the antibiotic alone.

To further study the pathophysiological changes beyond LasB inhibition, we investigated the cellular and humoral immune response. Here, we observed a clear impact of the combination treatment on eye neutrophil levels – key‐markers of the acute innate immune response – which were significantly reduced when compared to the sham‐treatment group (Figure 3C). Notably, when compared to the uninfected controls, eye neutrophil levels were statistically significantly higher in all infection groups, except for the **(*R*)‐30** plus meropenem group. For the myeloperoxidase (MPO) levels, significant increase was observed for all infection groups when compared to the non‐infected control (Figure 3D). However, when compared to the sham‐treated control, all groups that received treatment displayed lowered MPO levels. These findings suggest that the LasB inhibitor, even in the absence of the antibiotic, is likely to alter the release of MPO at the infection site. Further support for an immune‐cell modulating activity of **(*R*)‐30** was obtained by the determination of the contents of the neutrophil attracting and activating cytokines/chemokines interleukin 1β (IL‐1β), keratinocyte chemoattractant (KC), and tumor necrosis factor α (TNFα) in the tissue homogenates (Figure 3E–G). Treatment of the infected eyes with the LasB inhibitor **(*R*)‐30** allowed for a significant reduction in IL‐1β levels when compared to sham treatment, although not to the levels seen with the meropenem and combination treatment, respectively, which were almost on the level of this cytokine seen in uninfected eyes (Figure 3E). A different picture emerged for the murine IL‐8 homolog KC (Figure 3F). KC levels in the eyes of infected and sham‐treated mice as well as in combination therapy‐treated mice were found to be on the same level as the KC levels seen in uninfected eyes, while significantly higher amounts of this chemokine were seen in meropenem and LasB inhibitor‐treated mice. No clear differences in the contents of TNFα were observed in the eyes of all groups (Figure 3G). We further determined levels of **(*R*)‐30** in eye homogenate and serum (Figure S15, Supporting Information) and confirmed exposure of the pathoblocker in the eye, enabling the effects observed here. Serum levels showed that some compound reached the system. Yet, with average levels below 500 nM, compared to the test concentrations (100 µM) in the off‐target screening described above, suggesting that a safe ocular administration is achievable. The high levels of KC in eye homogenates of LasB‐inhibitor‐treated mice are probably explained by efficient inhibition of LasB by **(*R*)‐30** in the infected eye tissue, as KC/LasB co‐incubation studies confirmed KC to be a substrate for this protease and that the proteolytic activity of LasB on KC is effectively blocked by **(*R*)‐30** (Figure S16, Supporting Information).

Altogether, these findings suggest that a LasB inhibitor treatment is able to affect the immune response in infected eye tissue, but without altering the bacterial load and neutrophil content at the infection site. However, a significant impact on the disease development can be reached, when the LasB inhibitor is combined with an antibiotic, leading to noticeable reduction in symptoms of the infection and to a significant reduction in the number of bacteria at the infection site, which is clearly superior to treatment with the antibiotic alone.

## 3 Conclusions

*P. aeruginosa* is a priority pathogen responsible for a variety of diseases. In order to tackle this critical and increasingly resistant pathogen, antivirulence approaches have recently gained significant attention. After reporting on a phosphonate‐based LasB inhibitor with in vivo activity against lung infections, we have now developed this compound class further, assessing its application for the treatment of bacterial keratitis. In a substrate‐inspired approach, a dipeptide backbone was introduced, leading to single‐digit nanomolar on‐target activity for five LasB inhibitors (**9, 27, 30, (*R*)‐30**, and **35**).

Furthermore, the binding modes of compounds **9**, **30**, and **31** were elucidated using X‐ray crystallography, and the influence of the peptidic leucine linker was investigated. Additional interactions with the amino acid side chains of LasB were identified and were mainly responsible for the increased activity of the compounds. In contrast to that, an intramolecular hydrogen bond for the inhibitor was observed in the co‐crystal structure of LasB in complex with compound **30**. This presumably freezes a favored conformation, which explains the single‐digit nanomolar activity of **30**.

While potential off‐target effects were observed at high concentrations in vitro, frontrunner **(*R*)‐30** was well‐tolerated in vivo. In a murine infection model, it improved the activity of meropenem in the treatment of *Pseudomonas* keratitis, when applied in combination, and altered the immune response in infected eye tissue when applied alone.

Taken together, these findings expand the potential of phosphonate‐based LasB inhibitors from the known in vivo proof‐of‐concept after inhalative administration to the treatment of *Pseudomonas* keratitis, highlighting the versatility of the scaffold in tackling the virulence of this critical pathogen in vivo.

## 4 Experimental Section

Materials and experimental details are provided in the Supporting Information.

## Conflict of Interest

The authors declare the following competing financial interest: A.F.K, C.S., A.M.K, R.P.J, A.S.A, J.H, and A.K.H.H are co‐inventors on the international patent application (PCT/EP2021/073381) that incorporates methods outlined in this manuscript. Furthermore, the authors C.S., C.N.E., S.S., A.M.K., R.S., N.A.W., M.B., J.H., and A.K.H.H. are co‐inventors on a patent application (EP24178040.2) that was submitted on May 24th, 2024.

## Supporting information



Supporting Information

## Data Availability

The data that support the findings of this study are available from the corresponding author upon reasonable request.
