# Supplementary material for: Dipeptidic Phosphonates: Potent Inhibitors of Pseudomonas aeruginosa Elastase B Showing Efficacy in a Murine Keratitis Model
Source: Adv Sci (Weinh). 2025 Feb 19;12(14):2411807. doi: 10.1002/advs.202411807 (PMC11984924; doi:10.1002/advs.202411807)
Supplement: Supplementary file 1 — Supporting Information [file ADVS-12-2411807-s001.pdf]

ADVANCED SCIENCE
Open Access

Supporting Information

for *Adv. Sci.*, DOI 10.1002/advs.202411807

Dipeptidic Phosphonates: Potent Inhibitors of *Pseudomonas aeruginosa* Elastase B Showing Efficacy in a Murine Keratitis Model

*Alexander F. Kiefer, Christian Schütz, Colya N. Englisch, Dominik Kolling, Samira Speicher, Andreas M. Kany, Roya Shafiei, Noran A. Wadood, Ahmad Aljohmani, Niklas Wirschem, Ravindra P. Jumde, Andreas Klein, Asfandyar Sikandar, Yu-Mi Park, Gabriela Krasteva-Christ, Daniela Yildiz, Ahmed S. Abdelsamie, Katharina Rox, Jesko Köhnke, Rolf Müller, Markus Bischoff, Jörg Haupenthal and Anna K. H. Hirsch**

Supporting Information

Dipeptidic Phosphonates: Potent Inhibitors of *Pseudomonas aeruginosa* Elastase B Showing Efficacy in a Murine Keratitis Model

Alexander F. Kiefer[+,a,b,j], Christian Schütz[+,a,b,j], Colya N. Englisch[c], Dominik Kolling[d], Samira Speicher[a,b,j], Andreas M. Kany[a,b,j], Roya Shafiei[a,b,h,j], Noran A. Wadood[e,j], Ahmad Aljohmani[f,j], Niklas Wirschem[a,b,h,j], Ravindra P. Jumde[a,b,j], Andreas Klein[a,b,h,j], Asfandyar Sikandar[a,b,j], Yu-Mi Park[a,b,h,j], Gabriela Krasteva-Christ[e,j], Daniela Yildiz[f,j], Ahmed S. Abdelsamie[a,b,j], Katharina Rox[b,g], Jesko Köhnke[d], Rolf Müller[a,b,h,i,j], Markus Bischoff[c], Jörg Haupenthal[a,b,j] and Anna K. H. Hirsch*[,a,b,h,i,j]

Affiliation(s)

[a] A. F. Kiefer, C. Schütz, S. Speicher, A. M. Kany, R. Shafiei, N. Wirschem, R. P. Jumde, A. Klein, A. Sikandar, Y. Park, A. S. Abdelsamie, R. Müller, J. Haupenthal, A. K. H. Hirsch

Helmholtz Institute for Pharmaceutical Research Saarland (HIPS), Campus E8.1, 66123 Saarbrücken, Germany; Helmholtz Centre for Infection Research (HZI), Inhoffenstraße 7, 38124 Braunschweig, Germany

Email: anna.hirsch@helmholtz-hips.de

[b] A. F. Kiefer, C. Schütz, S. Speicher, A. Kany, R. Shafiei, N. Wirschem, R. P. Jumde, A. Klein, A. Sikandar, Y. Park, A. S. Abdelsamie, K. Rox, R. Müller, J. Haupenthal, A. K. H. Hirsch

German Center for Infection Research (DZIF) Inhoffenstraße 7, 38124 Braunschweig, Germany

[c] C. N. Englisch, M. Bischoff

Institute of Medical Microbiology and Hygiene, Saarland University, Kirrbergerstraße 100, 66421 Homburg/Saar, Germany

[d] D. Kolling, J. Köhnke

Institute for Food Chemistry, Callinstraße 5, 30167 Hannover, Germany

[e] N. A. Wadood, G. Krasteva-Christ

Institute of Anatomy and Cell Biology, Saarland University, Kirrbergerstraße 100, 66421 Homburg/Saar, Germany

[f] A. Aljohmani, D. Yildiz

Institute of Experimental and Clinical Pharmacology and Toxicology, PZMS, ZHMB, Saarland University, Kirrbergerstraße 100, 66421 Homburg/Saar, Germany

[g] K. Rox

Department of Chemical Biology, Helmholtz Centre for Infection Research (HZI), Inhoffenstraße 7, 38124 Braunschweig, Germany

[h] R. Shafiei, N. Wirschem, A. Klein, Y. Park, R. Müller, A. K. H. Hirsch

Department of Pharmacy, Saarland University, Campus E8.1, 66123 Saarbrücken, Germany

[i] R. Müller, A. K. H. Hirsch

Helmholtz International Lab for Anti-Infectives, Campus E8.1, 66123 Saarbrücken, Germany

[j] A. F. Kiefer, C. Schütz, S. Speicher, A. M. Kany, R. Shafiei, N. A. Wadood, A. Aljohmani, N. Wirschem, R. P. Jumde, A. Klein, A. Sikandar, Y. Park, A. S. Abdelsamie, G. Krasteva-Christ, D. Yildiz, R. Müller, J. Haupenthal, A. K. H. Hirsch

PharmaScienceHub (PSH), Campus A2.3, 66123 Saarbrücken, Germany

[+] Authors contributed equally.

Table of Contents

1. Chemistry 3

1.1 General procedure 1 (GP1): Synthesis of aniline substituted derivatives via mixed anhydride 3

1.2 General procedure 1a (GP1a): Synthesis of aniline substituted derivatives via EDC/HOBt coupling 4

1.3 General procedure 2 (GP2): Synthesis of phosphor containing dipeptides with varying amino acids via TBTU coupling 4

1.4 General procedure 2a (GP2a): Synthesis of phosphorus-containing dipeptides via EDC/HOBt coupling 5

1.5 General procedure 3 (GP3): Synthesis of phosphor containing dipeptides with varying aryl groups 5

2. X-ray crystallography 51

3. Biological evaluation 56

3.1 In vitro inhibition assays 56

3.2 Kinetic solubility 56

3.3 Lipophilicity determination 56

3.4 Metabolic stability in liver S9 fractions 56

3.5 Plasma stability 57

3.6 Calu-3 Permeability 58

3.7 In vivo pharmacokinetic studies 59

3.8 Test against zinc metalloproteases MMP1–3 and TACE (ADAM17), and testing of the Safety Screen44™ 62

3.9 Cytotoxicity studies 64

3.10 Evaluation of Zebrafish embryo toxicity 65

3.11 Tolerability study in male CD1 mice 65

3.12 Antibacterial effects 69

3.13 Inhibition of biofilm formation 71

3.14 In vivo target engagement studies using *Pseudomonas* keratitis model 72

3.15 Bioanalytical sample preparation of keratitis samples for determination of concentration of (*R*)-30 in eye and serum 79

3.16 LabB-KC studies 80

3. Statistical Analysis 82

4. References 83

1. Chemistry

All air- or moisture-sensitive reactions were carried out in dried glassware (>100 °C) under an atmosphere of nitrogen or argon. Dried solvents were distilled before use. Analytical TLC was performed on pre-coated silica gel plates (Macherey-Nagel, Polygram®SIL G/UV254). Visualization was accomplished with UV-light, $KMnO_4$ or a ceric ammonium molybdate chamber. The products were purified by flash chromatography on silica gel columns (Macherey-Nagel 60, 0.04-0.063 mm). Preparative high performance liquid chromatography (HPLC) was performed on a Waters Autopurifier System (APS) with a Phenomenex Gemini C18 column (250 × 4.6 mm, particle size 5 μm) as an analytical column for method development and a Phenomenex Gemini C18 column (250 × 19 mm, particle size 5 μm) for preparative separation. Detection was performed using mass trigger. ^{1}H-, and ^{13}C-spectra were recorded with a Bruker AV 500 [500 MHz, (^{1}H), 126 MHz (^{13}C)] spectrometer in $CDCl_3$, DMSO-d_6 or MeOH-d_4 unless otherwise specified. Chemical shifts are given in parts per million (ppm) and referenced against the residual proton or carbon resonances of the >99% deuterated solvents as internal standard. Coupling constants (*J*) are given in Hertz (Hz). Data are reported as follows: chemical shift, multiplicity (s = singlet, d = doublet, t =triplet, q = quartet, m = multiplet, dd = doublet of doublets, dt =doublet of triplets, br = broad and combinations of these) coupling constants, and integration. NMR spectra were evaluated using ACDLabs 2019. Liquid chromatography-mass spectrometry (LC-MS) was performed on a LC-MS system, consisting of a Dionex UltiMate 3000 pump, autosampler, column compartment, detector (Thermo Fisher Scientific, Dreieich, Germany) and ESI quadrupole MS (MSQ Plus or ISQ EC, Thermo Fisher Scientific,Dreieich, Germany). High-resolution mass was determined by LC-MS/MS using Thermo Scientific Q Exactive Focus Orbitrap LC-MS/MS system. Purity of the final compounds was determined by LC-MS using the area percentage method on the UV trace recorded at a wavelength of 254 nm and found to be >95%.

1.1 General procedure 1 (GP1): Synthesis of aniline substituted derivatives via mixed anhydride

The corresponding Boc-protected amino acid (1.0 equiv.) was dissolved in THF (0.1 M) and cooled down to −20 °C. Then NMM (2.5 equiv.) and isobutyl chloroformate (1.0 equiv.) were added dropwise. The reaction mixture was stirred at this temperature for 30 minutes and then the 3,4-dichloroaniline (1.0 equiv.), dissolved in THF (1 M), was added. After the reaction mixture had reached rt, it was diluted with EtOAc. The organic phase was washed with $KHSO_4$

(1 N) solution, saturated $NaHCO_3$ solution and saturated aqueous NaCl solution, dried over anhydrous Na_2SO_4, filtered and concentrated under reduced pressure. Column chromatographic purification afforded the corresponding peptide.

1.2 General procedure 1a (GP1a): Synthesis of aniline substituted derivatives via EDC/HOBt coupling

The corresponding Boc-protected amino acid (1.0 equiv.), 3,4-dichloroaniline (1.2 equiv.), EDC·HCl (4.0 equiv.), and HOBt (4.0 equiv.) was dissolved in DCM (0.125 M). To the resulting solution, was added DIPEA (5.0 equiv.) and kept stirring at rt. After complete conversion (LCMS), the reaction mixture was diluted with EtOAc (20 mL), and water (20 mL) was added. The organic phase was removed, and the aqueous phase was extracted with EtOAc (3 x 20mL). The combined organic phases were dried over anhydrous Na_2SO_4, filtered and concentrated under reduced pressure. Column chromatographic purification afforded the corresponding peptide.

1.3 General procedure 2 (GP2): Synthesis of phosphor containing dipeptides with varying amino acids via TBTU coupling

The Boc-protected peptide (1.0 equiv.) was dissolved in DCM (0.1 M) and treated at 0 °C with HCl (10.0 equiv., 4 M in dioxane). The mixture was warmed up to rt and after complete conversion (TLC), the solvent was removed under reduced pressure with the result that the crystalline hydrochloride remained, which was subsequently dissolved in DMF (0.1 M). 2-(diethoxyphosphoryl)-4-methylpentanoic acid (1.1 equiv.) was added to this solution, and the reaction mixture was cooled to 0 °C. Coupling was achieved by TBTU (1.1 equiv.) and NMM (2.5 equiv.). The reaction mixture was warmed up to rt and after complete conversion (TLC), diluted with EtOAc and washed successively with 1 N $KHSO_4$ solution, saturated aqueous $NaHCO_3$ solution and saturated aqueous NaCl solution. The organic layer was dried over anhydrous Na_2SO_4, filtered, and the residue was used without further purification for the next step.

To a solution of diethyl phosphonate dipeptide (1.0 equiv.) in dichloromethane (0.1 M), bromotrimethylsilane (7.0 equiv.) was added dropwise over a period of 15 min. The reaction mixture was stirred at room temperature overnight. Then MeOH was added and stirred at room temperature for 30 min to cleave the previously formed TMS ester. The solvents were removed under reduced pressure, and the crude product was purified *via* preparative HPLC.

1.4 General procedure 2a (GP2a): Synthesis of phosphorus-containing dipeptides via EDC/HOBt coupling

The Boc-protected peptide (1.0 equiv.) was dissolved in DCM (0.1 M) and treated at 0 °C with HCl (10.0 equiv., 4 M in dioxane). The mixture was warmed up to rt, and after complete conversion (TLC), the solvent was removed under reduced pressure with the result that the crystalline hydrochloride remained, which was subsequently dissolved in DMF (0.1 M) along with 2-(diethoxyphosphoryl)-4-methylpentanoic acid **6** (1.2–1.5 equiv.), HOBt.H_2O (2.0 equiv.). To the resulting solution were added EDC·HCl (2.0 equiv.) and DIPEA (2.5 equiv.) and kept stirring at rt. After complete conversion (LCMS), the reaction mixture was diluted with EtOAc and water was added. The organic phase was removed, and the aqueous phase was extracted with EtOAc (x 3). The combined organic phases were dried over anhydrous Na_2SO_4, filtered and concentrated under reduced pressure, and the residue was used without further purification for the next step.

To a solution of diethyl phosphonate dipeptide (1.0 equiv.) in dichloromethane (0.1 M), bromotrimethylsilane (7.0 equiv.) was added dropwise over 15 min. The reaction mixture was stirred at room temperature. After complete conversion (LCMS), MeOH was added and stirred at room temperature for 30 min to cleave the previously formed TMS ester. The volatiles were removed under reduced pressure and the crude product was purified *via* preparative HPLC.

1.5 General procedure 3 (GP3): Synthesis of phosphor containing dipeptides with varying aryl groups

(2-(diethoxyphosphoryl)-4-methylpentanoyl)-L-valine (1.0 equiv.) was dissolved in DCM. The corresponding amine (1.1 equiv.) and NMM (2.5 equiv.) were added, and the reaction mixture was cooled to 0°C. Subsequently, TBTU (1.1 equiv.) was added. The reaction mixture was warmed up to rt, and progress was monitored by LC-MS. Upon completion, the reaction mixture was diluted with EtOAc and washed with 1M NaOH solution (2x) and saturated aqueous NaCl solution. The organic layer was dried over anhydrous Na_2SO_4, filtered, and concentrated under reduced pressure. The residue was used without further purification for the next step.

To a solution of diethyl phosphonate dipeptide (1.0 equiv.) in dichloromethane (0.1 M), bromotrimethylsilane (7.0 equiv.) was added dropwise over a period of 15 min. The reaction mixture was stirred at room temperature overnight. Then MeOH was added and stirred at room temperature for 30 min to cleave the previously formed TMS ester. The solvents were removed under reduced pressure, and the crude product was purified *via* preparative HPLC.

2-(Diethoxyphosphoryl)-4-methylpentanoate (6a)

Literature reported ethyl 2-bromo-4-methylpentanoate[1] (14.45 g, 64.7 mmol) and $P(OEt)_3$ (22.41 mL, 129.4 mmol) were mixed and heated for 48 h at 150 °C. After that, the mixture was cooled down to rt, and Et_2O (350 mL) was added. The mixture was transferred into a separatory funnel and washed with saturated aqueous NaCl solution (2 x 350 mL), dried over $MgSO_4$, filtered and concentrated under reduced pressure. The product was purified using flash chromatography (SiO_2, hexanes/EtOAc 1:1), and compound **6a** (7.93 g, 28.3 mmol, 44%) was obtained as pale-yellow oil.

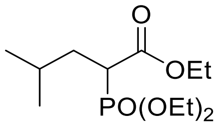

^1H NMR (500 MHz, $CDCl_3$) δ: 4.18 – 4.24 (m, 2H), 4.11 – .17 (m, 4H), 3.00 – 3.07 (m, 1H), 1.95 – 2.07 (m, 1H), 1.55 – 1.66 (m, 2H), 1.33 (dt, 6H, J = 2.3, 7.0 Hz), 1.28 (t, 3H, J = 7.2 Hz), 0.92 (d, 3H, J = 6.1 Hz), 0.89 (d, 3H, J = 6.3 Hz). ^{13}C NMR (126 MHz, $CDCl_3$) δ: 169.4 (d, J = 5.5 Hz), 62.7 (d, J = 6.4 Hz), 62.6 (d, J = 6.4 Hz), 61.3, 44.5, 43.4, 35.5 (d, J = 5.5 Hz), 26.9 (d, J = 14.7 Hz), 22.9, 21.2, 16.4 (d, J = 3.7 Hz), 16.3 (d, J = 3.7 Hz), 14.1. ^{31}P NMR (202 MHz, $CDCl_3$) δ: 23.4. HRMS (ESI+) calculated for $C_{12}H_{26}O_5P$ $[M+1]^+$ 281.1518, found: 281.1503.

2-(Diethoxyphosphoryl)-4-methylpentanoic acid (6)

The compound **6c** (7.93 g, 28.3 mmol) was dissolved in EtOH (270 mL), and NaOH (2.15 g, 53.9 mmol) in dist. H_2O (100 mL) was added. The mixture was stirred at room temperature overnight. The progress was monitored using LC-MS. After completion, the mixture was transferred into a separatory funnel, dist. water (300 mL) and Et_2O (400 mL) were added, and the layers were separated. The aqueous layer was acidified to pH = 1 using HCl (6 M), and extracted with EtOAc (3 x 300 mL). The combined organic layers were washed with saturated aqueous NaCl solution (2 x 500 mL), dried over $MgSO_4$, filtered and concentrated under reduced pressure. The compound **6** (6.63 g, 26.3 mmol, 98%) was obtained as a pale-yellow oil, which was used without further purification.

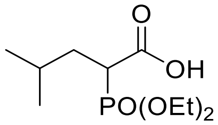

^1H NMR (500 MHz, $CDCl_3$) δ: 8.33 (br s, 2H), 4.13 – 4.25 (m, 4H), 3.07 (ddd, 1H, J = 3.1, 11.3, 23.0 Hz), 1.99 (dddd, 1H, J = 4.8, 8.5, 11.4, 13.5 Hz), 1.58 – 1.70 (m, 1H), 1.49 –1.57 (m, 1H),

1.33 (dt, 6H, *J* = 2.7, 7.1 Hz), 0.92 (d, 3H, *J* = 6.6 Hz), 0.89 (d, 3H, *J* = 6.6 Hz). ^{13}C NMR (126 MHz, $CDCl_3$) δ: 171.9 (d, *J* = 3.7 Hz), 63.7 (d, *J* = 6.4 Hz), 62.9 (d, *J* = 6.4 Hz), 44.4, 43.4, 35.6 (d, *J* = 5.5 Hz), 26.8 (d, *J* = 13.8 Hz), 23.0, 21.2, 16.3 (d, *J* = 2.8 Hz), 16.2 (d, *J* = 2.8 Hz).^{31}P NMR (202 MHz, $CDCl_3$) δ: 24.3. HRMS (ESI+) calculated for $C_{10}H_{22}O_5P$ [M+1]$^+$ 253.1205, found: 253.1191.

***Tert*-butyl (2-((3,4-dichlorophenyl)amino)-2-oxoethyl)carbamate (7a)**

According to **GP1**, (*tert*-butoxycarbonyl)-glycine (500 mg, 2.85 mmol) was reacted with NMM (793 µL, 7.13 mmol), IBCF (369 µL, 2.85 mmol) and 3,4-dichloroaniline (462 mg, 2.85 mmol). After purification by column chromatography (SiO_2, hexanes/EtOAc 3:2), the corresponding *tert*-butyl (2-((3,4-dichlorophenyl)amino)-2-oxoethyl)carbamate **7a** (455.6 mg, 1.42 mmol, 50%) was obtained as white amorphous solid.

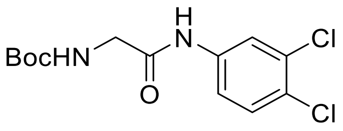

^{1}H NMR (500 MHz, $CDCl_3$) δ: 8.41 (br s, 1H), 7.76 (d, *J* = 2.0 Hz, 1H), 7.32 – 7.39 (m, 2H), 5.24 (br s, 1H), 3.92 (d, *J* = 6.0 Hz, 2H), 1.50 (s, 9H). ^{13}C NMR (126 MHz, $CDCl_3$) δ: 167.9, 156.7, 137.0, 132.8, 130.5, 127.7, 121.5, 119.0, 81.2, 45.9, 28.3. HRMS (ESI+) calculated for $C_{13}H_{17}Cl_2N_2O_3$ [M+H]$^+$ 319.0611, found 319.0615.

***Tert*-butyl (*S*)-(1-((3,4-dichlorophenyl)amino)-1-oxopropan-2-yl)carbamate (8a)**

According to **GP1**, (*tert*-butoxycarbonyl)-L-alanine (378 mg, 2.00 mmol) was reacted with NMM (550 µL, 5.0 mmol), IBCF (259 µL, 2.0 mmol) and 3,4-dichloroaniline (324 mg, 2.00 mmol). After purification by column chromatography (SiO_2, hexanes/EtOAc 9:1), the corresponding *tert*-butyl (*S*)-(1-((3,4-dichlorophenyl)amino)-1-oxopropan-2-yl)carbamate **8a** (637.1 mg, 1.91 mmol, 95%) was obtained as white amorphous solid.

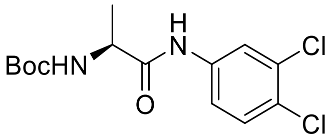

^{1}H NMR (500 MHz, $CDCl_3$) δ: 8.97 (br s, 1H), 7.70 – 7.77 (m, 1H), 7.27 (br s, 2H), 5.15 (br d, *J* = 7.2 Hz, 1H), 4.35 (br s, 1H), 1.47 (s, 9H), 1.43 ppm (d, *J* = 7.2 Hz, 3H). ^{13}C NMR (126 MHz, $CDCl_3$) δ: 171.2, 156.5, 137.4, 132.6, 130.3, 127.2, 121.3, 118.8, 81.1, 50.9, 28.3, 17.3. HRMS (ESI+) calculated for $C_{14}H_{19}Cl_2N_2O_3$ [M+H]$^+$ 333.0767, found 333.0762.

Tert-Butyl (_S_)-(1-((3,4-dichlorophenyl)amino)-3-methyl-1-oxobutan-2-yl)-carbamate (9a)

According to **GP1**, (*tert*-butoxycarbonyl)-L-valine (434 mg, 2.0 mmol, 1.0 equiv.) was reacted with NMM (550 µL, 5.0 mmol), IBCF (259 µL, 2.0 mmol) and 3,4-dichloroaniline (324 mg, 2.0 mmol). After purification by column chromatography (SiO_2, hexanes/EtOAc 9:1), the corresponding *tert*-butyl (*S*)-(1-((3,4-dichlorophenyl)amino)-3-methyl-1-oxobutan-2-yl)-carbamate **9a** (523.8 mg, 1.44 mmol, 72%) was obtained as white amorphous solid.

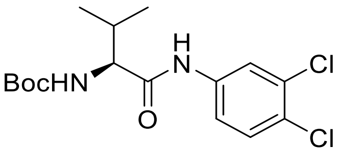

^{1}H NMR (500 MHz, $CDCl_3$) δ: 8.85 (br s, 1H), 7.70 (br s, 1H), 7.2 – 7.3 (m, 2H), 5.27 (br d, 1H, *J* = 8.2 Hz), 4.06 (br t, 1H, *J* = 7.6 Hz), 2.15 (br d, 1H, *J* = 6.1 Hz), 1.47 (s, 9H), 1.03 (dd, 6H, *J* = 2.7, 6.7 Hz). ^{13}C NMR (126 MHz, $CDCl_3$,) δ: 170.7, 137.2, 132.5, 130.2, 121.2, 118.6, 61.1, 30.5, 28.3, 19.3, 18.4. HRMS (ESI+) calculated for $C_{16}H_{23}Cl_2N_2O_3$ $[M+H]^+$ 361.1080, found 361.1080.

Tert-butyl (_R_)-(1-((3,4-dichlorophenyl)amino)-3-methyl-1-oxobutan-2-yl)-carbamate (10a)

According to **GP1**, (*tert*-butoxycarbonyl)-D-valine (434 mg, 2.0 mmol) was reacted with NMM (550 µL, 5.0 mmol), IBCF (259 µL, 2.0 mmol) and 3,4-dichloroaniline (324 mg, 2.0 mmol). After purification by column chromatography (SiO_2, hexanes/EtOAc 9:1), the corresponding *tert*-butyl (*S*)-(1-((3,4-dichlorophenyl)amino)-3-methyl-1-oxobutan-2-yl)carbamate **10a** (345.5 mg, 0.96 mmol, 48%) was obtained as white amorphous solid.

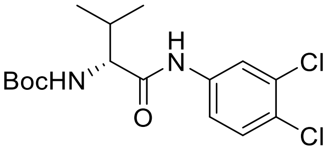

^{1}H NMR (500 MHz, $CDCl_3$) δ: 8.81 (br s, 1H), 7.73 (br s, 1H), 7.21 _ 7.26 (m, 2H), 5.31 (br d, *J* = 6.6 Hz, 1H), 4.05 (br t, *J* = 7.8 Hz, 1H), 2.10 – 2.22 (m, 1H), 1.46 (s, 9H), 1.00 – 1.04 (m, 6H). ^{13}C NMR (126 MHz, $CDCl_3$) δ: 170.7, 156.7, 137.1, 132.6, 130.2, 121.3, 118.8, 61.1, 30.5, 28.3, 19.3, 18.4. HRMS (ESI+) calculated for $C_{16}H_{23}Cl_2N_2O_3$ $[M+H]^+$ 361.1080, found 361.1082.

Tert-butyl (_R_)-(1-((3,4-dichlorophenyl)amino)-4-methyl-1-oxopentan-3-yl)carbamate (11a)

According to **GP1**, (*R*)-3-((*tert*-butoxycarbonyl)amino)-4-methylpentanoic acid (263 mg, 1.14 mmol) was reacted with NMM (313 µL, 2.85 mmol), IBCF (148 µL, 1.14 mmol) and 3,4-dichloroaniline (184 mg, 1.14 mmol). After purification by column chromatography (SiO_2, $CHCl_3$/MeOH 9:1), the corresponding *tert*-butyl (*R*)-(1-((3,4-dichlorophenyl)amino)-4-methyl-1-oxopentan-3-yl)carbamate **11a** (339.6 mg, 0.91 mmol, 80%) was obtained as white amorphous solid.

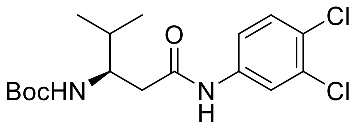

^{1}H NMR (500 MHz, $CDCl_3$) δ: 8.82 (br s, 1H), 7.86 (br s, 1H), 7.38 – 7.44 (m, 1H), 7.28 – 7.37 (m, 1H), 4.85 (br d, *J* = 9.2 Hz, 1H), 3.74 – 3.83 (m, 1H), 2.62 – 2.71 (m, 1H), 2.49 – 2.62 (m, 1H), 1.80 – 1.90 (m, 1H), 1.44 (s, 9H), 0.97 (t, *J* = 6.9 Hz, 6H). ^{13}C NMR (126 MHz, $CDCl_3$) δ: 169.6, 157.0, 137.8, 132.6, 130.3, 127.0, 121.4, 119.0, 80.5, 53.2, 42.2, 32.5, 28.3, 19.2, 18.1. HRMS (ESI+) calculated for $C_{17}H_{25}Cl_2N_2O_3$ $[M+H]^+$ 375.1237, found 375.1235.

Tert-butyl (_S_)-(1-cyclopropyl-2-((3,4-dichlorophenyl)amino)-2-oxoethyl)carbamate (12a)

According to **GP1**, (*S*)-2-((*tert*-butoxycarbonyl)amino)-2-cyclopropylacetic acid (215 mg, 1.00 mmol) was reacted with NMM (121 mL, 1.10 mmol), IBCF (130 µL, 1.00 mmol) and 3,4-dichloroaniline (162 mg, 1.00 mmol). Automated Flash purification (SiO_2, hexanes/EtOAc 7:3) afforded the title **12a** compound as a colorless solid (298.0 mg, 0.830 mmol, 83%).

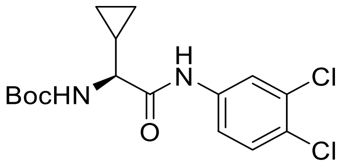

^{1}H NMR (500 MHz, $CDCl_3$) δ = 8.97 (bs, 1 H), 7.67 (s, 1 H), 7.21 – 7.19 (m, 2 H), 5.44 – 5.38 (m, 1 H), 3.60 – 3.53 (m, 1 H), 1.48 (s, 9 H), 1.22 – 1.14 (m, 1 H), 0.68 – 0.65 (m, 1 H), 0.64 – 0.63 (m, 1 H), 0.62 – 0.58 (m, 1 H), 0.44 – 0.40 (m, 1 H). ^{13}C NMR (126 MHz, $CDCl_3$) δ = 170.3, 156.5, 137.4, 132.5, 130.1, 127.0, 121.1, 118.5, 80.9, 59.6, 28.3, 13.4. LCMS: t_R = 5.02, m/z = 304 $[M+H-tBu]^+$

Tert-butyl (_S_)-(1-cyclobutyl-2-((3,4-dichlorophenyl)amino)-2-oxoethyl)carbamate (13a)

According to **GP1**, (_S_)-2-((_tert_-butoxycarbonyl)amino)-2-cyclobutylacetic acid (115 mg, 0.500 mmol) was reacted with NMM (61 mL, 0.550 mmol), IBCF (65 µL, 0.500 mmol) and 3,4-dichloroaniline (81 mg, 0.500 mmol). Automated Flash purification (SiO_2, hexanes/EtOAc 7:3) afforded the title compound **13a** as a colorless solid (171.0 mg, 0.458 mmol, 92%).

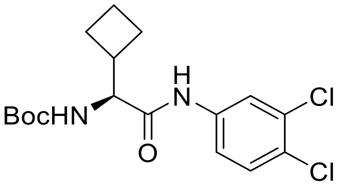

^{1}H NMR (500 MHz, $CDCl_3$) δ = 8.81 (bs, 1 H), 7.72 (s, 1 H), 7.27 – 7.22 (m, 2 H), 5.10 – 5.03 (m, 1 H), 4.21 – 4.14 (m, 1 H), 2.76 – 2.67 (m, 1 H), 2.13 – 2.07 (m, 2 H), 1.97 – 1.93 (m, 2 H), 1.92 – 1.85 (m, 2 H), 1.47 (s, 9 H). ^{13}C NMR (126 MHz, $CDCl_3$) δ = 169.8, 156.5, 137.1, 132.3, 129.9, 126.9, 120.9, 118.4, 80.7, 69.6, 59.5, 36.2, 28.0, 25.0, 17.8. LCMS: t_R = 5.20, m/z = 317 $[M+H\text{-}tBu]^+$

Tert-butyl (_S_)-(1-cyclohexyl-2-((3,4-dichlorophenyl)amino)-2-oxoethyl)-carbamate (14a)

According to **GP1**, (_S_)-2-((_tert_-butoxycarbonyl)amino)-2-cyclohexylacetic acid (500 mg, 1.94 mmol) was reacted with NMM (533 µL, 4.85 mmol), IBCF (251 µL, 1.94 mmol) and 3,4-dichloroaniline (314 mg, 1.94 mmol). After purification by column chromatography (SiO_2, hexanes/EtOAc 9:1), the corresponding _tert_-butyl (_S_)-(1-cyclohexyl-2-((3,4-dichlorophenyl)amino)-2-oxoethyl)carbamate **14a** (336.5 mg, 0.84 mmol, 43%) was obtained as white amorphous solid.

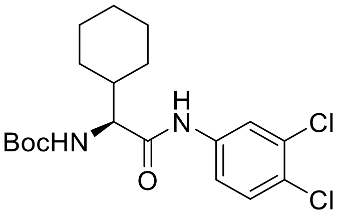

^{1}H NMR (500 MHz, $CDCl_3$) δ: 9.10 (br s, 1H), 7.70 (d, J = 2.0 Hz, 1H), 7.10 – 7.25 (m, 2H), 5.39 (d, J = 8.4 Hz, 1H), 4.04 – 4.15 (m, 1H), 1.90 (br d, J = 11.9 Hz, 1H), 1.70 – 1.80 (m, 3H), 1.68 (br s, 2H), 1.46 (s, 9H), 1.17-1.28 (m, 3H), 1.10 – 1.17 (m, 1H), 0.96 – 1.08 (m, 1H). ^{13}C NMR (126 MHz, $CDCl_3$) δ: 170.8, 156.8, 137.2, 132.5, 130.1, 127.1, 121.1, 118.5, 80.6, 60.6, 40.0, 29.6, 29.1, 28.3, 26.0, 25.8, 25.8. HRMS (ESI+) calculated for $C_{19}H_{27}Cl_2N_2O_3$ $[M+H]^+$ 401.1393, found 401.1390.

***Tert*-butyl ((2*S*,3*S*)-1-((3,4-dichlorophenyl)amino)-3-methyl-1-oxopentan-2-yl)carbamate (15a)**

According to **GP1**, (*tert*-butoxycarbonyl)-L-isoleucine (481 mg, 2.00 mmol) was reacted with NMM (550 µL, 5.0 mmol), IBCF (259 µL, 2.00 mmol) and 3,4-dichloroaniline (324 mg, 2.00 mmol). After purification by column chromatography (SiO_2, hexanes/EtOAc 9:1), the corresponding *tert*-butyl ((2*S*,3*S*)-1-((3,4-dichlorophenyl)amino)-3-methyl-1-oxopentan-2-yl)carbamate **15a** (475.5 mg, 1.26 mmol, 63%) was obtained as white amorphous solid.

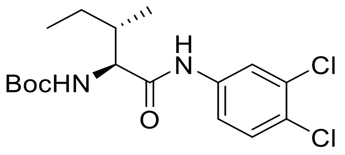

^{1}H NMR (500 MHz, $CDCl_3$) δ: 8.87 (br s, 1H), 7.67 – 7.74 (m, 1H), 7.16 – 7.26 (m, 2H), 5.25 (br d, *J* = 8.5 Hz, 1H), 4.04 – 4.16 (m, 1H), 1.86 – 1.97 (m, 1H), 1.63 (br s, 1H), 1.46 (s, 9H), 1.12 _ 1.29 (m, 1H), 1.00 (d, *J* = 6.9 Hz, 3H), 0.93 (t, *J* = 7.4 Hz, 3H). ^{13}C NMR (126 MHz, $CDCl_3$) δ: 170.8, 156.7, 137.2, 132.5, 130.2, 127.2, 121.2, 118.6, 80.7, 36.7, 28.3, 25.0, 15.5, 10.9. HRMS (ESI+) calculated for $C_{17}H_{25}Cl_2N_2O_3$ $[M+H]^+$ 375.1237, found 375.1240.

***Tert*-butyl (*S*)-(3-cyclopropyl-1-((3,4-dichlorophenyl)amino)-1-oxopropan-2-yl)carbamate (16a)**

According to **GP1**, (*S*)-2-((*tert*-butoxycarbonyl)amino)-3-cyclopropylpropanoic acid (500 mg, 2.18 mmol) was reacted with NMM (599 µL, 5.45 mmol), IBCF (283 µL, 2.18 mmol) and 3,4-dichloroaniline (353 mg, 2.18 mmol). After purification by column chromatography (SiO_2, hexanes/EtOAc 9:1), the corresponding *tert*-butyl (*S*)-(3-cyclopropyl-1-((3,4-dichlorophenyl)amino)-1-oxopropan-2-yl)carbamate **16a** (814.0 mg, 2.18 mmol, quant.) was obtained as white amorphous solid.

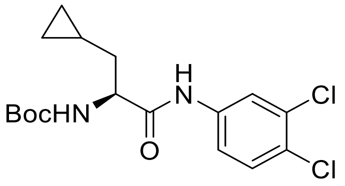

^{1}H NMR (500 MHz, $CDCl_3$) δ: 8.74 (br s, 1H), 7.71 – 7.76 (m, 1H), 7.27 – 7.32 (m, 2H), 5.21 (br d, *J* = 6.9 Hz, 1H), 4.30 (br s, 1H), 1.65 – 1.79 (m, 2H), 1.47 (s, 9H), 0.72 – 0.85 (m, 1H), 0.46 – 0.58 (m, 2H), 0.08 – 0.18 ppm (m, 2H). ^{13}C NMR (126 MHz, $CDCl_3$) δ: 170.7, 156.5,

137.3, 132.6, 130.3, 127.3, 121.3, 118.8, 80.9, 56.0, 36.5, 28.3, 7.4, 4.5, 4.4. HRMS (ESI+) calculated for $C_{17}H_{23}Cl_2N_2O_3$ $[M+H]^+$ 373.1080, found 373.1082.

Tert-butyl (_S_)-(1-((3,4-dichlorophenyl)amino)-4-methyl-1-oxopentan-2-yl)-carbamate (17a)

According to **GP1**, (_tert_-butoxycarbonyl)-L-leucine (463 mg, 2.00 mmol) was reacted with NMM (550 µL, 5.0 mmol), IBCF (259 µL, 2.0 mmol) and 3,4-dichloroaniline (324 mg, 2.00 mmol). After purification by column chromatography (SiO_2, hexanes/EtOAc 9:1), the corresponding _tert_-butyl (_S_)-(1-((3,4-dichlorophenyl)amino)-4-methyl-1-oxopentan-2-yl)-carbamate **17a** (768.2 mg, 2.0 mmol, quant.) was obtained as white amorphous solid.

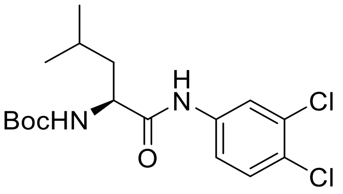

^{1}H NMR (500 MHz, $CDCl_3$) δ: 9.20 (br s, 1H), 7.69 (s, 1H), 7.19 (br s, 2H), 5.25 (br d, _J_ = 6.3 Hz, 1H), 4.25-4.39 (m, 1H), 2.05 (s, 1H), 1.75 (tq, _J_ = 13.4, 6.6 Hz, 1H), 1.58 – 1.69 (m, 2H), 1.46 (s, 9H), 0.95 (dd, _J_ = 16.3, 6.6 Hz, 6H). ^{13}C NMR (126 MHz, $CDCl_3$) δ: 171.6, 156.7, 137.5, 132.4, 130.1, 127.0, 121.1, 118.6, 80.9, 53.9, 40.7, 28.3, 24.7, 23.0, 21.5. HRMS (ESI+) calculated for $C_{17}H_{25}Cl_2N_2O_3$ $[M+H]^+$ 375.1237, found 375.1240.

Tert-butyl (S)-(1-((3,4-dichlorophenyl)amino)-3-methoxy-1-oxopropan-2-yl)carbamate (18a)

According to **GP1**, (_S_)-2-((_tert_-Butoxycarbonyl)amino)-3-methoxypropanoic acid (206 mg, 0.94 mmol) was reacted with NMM (261 µL, 2.35 mmol), IBCF (122 µL, 0.94 mmol) and 3,4-dichloroaniline (152 mg, 0.94 mmol). After purification by column chromatography (SiO_2, PB/EtOAc 9:1 to 3:1), the corresponding _tert_-butyl (S)-(1-((3,4-dichlorophenyl)amino)-3-methoxy-1-oxopropan-2-yl)carbamate **18a** (136.0 mg, 0.37 mmol, 39%) was obtained as white amorphous solid.

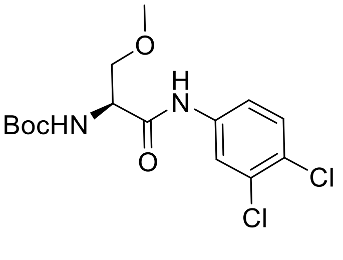

^{1}H NMR (500 MHz, CDCl3) δ = 8.50 (s, 1H), 7.77 (d, *J*=2.3, 1H), 7.39 – 7.31 (m, 2H), 5.51 – 5.36 (m, 1H), 4.12 (q, *J*=7.2, 1H), 3.88 (dd, *J*=9.3, 4.0, 1H), 3.55 (dd, *J*=9.3, 6.6, 1H), 3.43 (s, 3H), 1.48 (s, 9H). ^{13}C NMR (126 MHz, CD_2Cl_2) δ = 168.93, 137.11, 132.94, 130.63, 127.76, 121.68, 119.21, 71.69, 60.55, 59.43, 28.42, 21.20, 14.34. HRMS (ESI-) calculated for $C_{15}H_{19}Cl_2N_2O_4$ $[M-H]^-$ 361.0727, found 361.0728.

Tert-butyl (_S_)-(3-cyclohexyl-1-((3,4-dichlorophenyl)amino)-1-oxopropan-2-yl)carbamate (19a)

According to **GP1**, (*S*)-2-((*tert*-butoxycarbonyl)amino)-3-cyclohexylpropanoic acid (200 mg, 0.74 mmol) was reacted with NMM (202 µL, 1.84 mmol), IBCF (96 µL, 0.74 mmol) and 3,4-dichloroaniline (119 mg, 0.74 mmol). After purification by column chromatography (SiO_2, hexanes/EtOAc 9:1), the corresponding *tert*-butyl (*S*)-(3-cyclohexyl-1-((3,4-dichlorophenyl)amino)-1-oxopropan-2-yl)carbamate **19a** (267.7 mg, 0.65 mmol, 88%) was obtained as white amorphous solid.

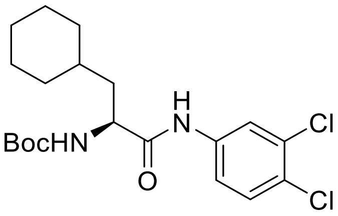

^{1}H NMR (500 MHz, $CDCl_3$) δ: 8.97 (br s, 1H), 7.72 (d, *J* = 2.0 Hz, 1H), 7.18 – 7.26 (m, 2H), 5.09 (d, *J* = 7.9 Hz, 1H), 4.32 (br s, 1H), 1.74 – 1.81 (m, 1H), 1.71 (br d, *J* = 10.2 Hz, 4H), 1.56 (ddd, *J* = 14.2, 9.3, 5.4 Hz, 1H), 1.47 (s, 9H), 1.34 – 1.45 (m, 1H), 1.10 – 1.30 (m, 4H), 0.84 – 1.03 (m, 2H). ^{13}C NMR (126 MHz, $CDCl_3$) δ: 171.4, 156.7, 137.4, 132.5, 130.2, 127.1, 121.2, 118.7, 80.9, 53.2, 39.1, 34.1, 33.7, 32.4, 28.3, 26.3, 26.2, 26.0. HRMS (ESI+) calculated for $C_{20}H_{29}Cl_2N_2O_3$ $[M+H]^+$ 415.1550, found 415.1557.

Tert-butyl (S)-(1-((3,4-dichlorophenyl)amino)-1-oxo-3-(tetrahydro-2H-pyran-4-yl)propan-2-yl)carbamate (20a)

Compound **20a** was prepared according to **GP1a**, using (*S*)-2-((tert-butoxycarbonyl)amino)-3-(tetrahydro-2H-pyran-4-yl)propanoic acid (50.0 mg, 0.18 mmol), 3,4-dichloroaniline (29.3 mg, 0.18 mmol), EDC·HCl (140.3 mg, 0.73 mmol), HOBt·H_2O (112.1 mg, 0.73 mmol), and DIPEA (159.3 uL, 0.91 mmol) in DCM (1.5 mL). After flash column chromatography (SiO_2, hexanes/EtOAc gradient 100–0 %) (*S*)-(1-((3,4-dichlorophenyl)amino)-1-oxo-3-(pyridin-2-yl)propan-2-yl)carbamate **20a** (47.0 mg, 0.11 mmol, 62%) was obtained as white amorphous solid.

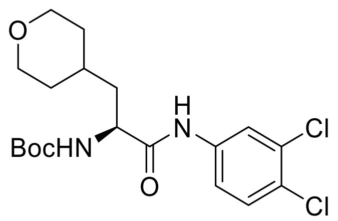

^{1}H NMR (500 MHz, $CDCl_3$) δ: 9.03 (s, 1H), 7.61 (d, *J* = 2.3 Hz, 1H), 7.22 – 7.06 (m, 2H), 5.27 (d, *J* = 8.3 Hz, 1H), 4.31 (s, 1H), 3.96 – 3.77 (m, 2H), 3.28 (dd, *J* = 13.6, 11.8, 2.0 Hz, 2H), 1.80 – 1.64 (m, 1H), 1.65 – 1.48 (m, 4H), 1.39 (s, 9H), 1.34 – 1.16 (m, 2H). ^{13}C NMR (126 MHz, $CDCl_3$) δ 170.2, 155.8, 136.2, 131.6, 129.2, 126.3, 120.2, 117.6, 80.1, 66.8, 66.7, 37.9, 32.2, 31.3, 30.6, 27.3. HRMS (ESI+) calculated for $C_{19}H_{27}Cl_2N_2O_4$ $[M+H]^+$ 417.1342, found 417.1331.

***Tert*-butyl (*S*)-2-((3,4-dichlorophenyl)carbamoyl)pyrrolidine-1-carboxylate (21a)**

According to **GP1**, (*tert*-butoxycarbonyl)-L-proline (430 mg, 2.0 mmol) was reacted with NMM (550 µL, 5.0 mmol), IBCF (259 µL, 2.0 mmol) and 3,4-dichloroaniline (324 mg, 2.0 mmol). After purification by column chromatography (SiO_2, hexanes/EtOAc 9:1), the corresponding *tert*-butyl (*S*)-2-((3,4-dichlorophenyl)carbamoyl)pyrrolidine-1-carboxylate **21a** (659.3 mg, 1.84 mmol, 92%) was obtained as white amorphous solid.

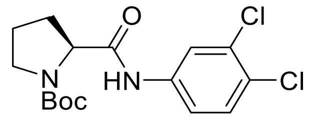

^{1}H NMR (500 MHz, $CDCl_3$) δ: 9.80 (br s, 1H), 7.81 (d, *J* = 2.3 Hz, 1H), 7.28 – 7.38 (m, 2H), 4.47 (br s, 1H), 3.44 (br s, 1H), 3.35 (br s, 1H), 2.53 (br s, 1H), 1.91 – 2.00 (m, 2H), 1.88 (br s, 1H), 1.51 (br s, 9H). ^{13}C NMR (126 MHz, $CDCl_3$) δ: 170.9, 156.9, 138.0, 130.3, 121.2, 118.7, 81.2, 60.4, 47.3, 28.4, 26.8, 24.6. HRMS (ESI+) calculated for $C_{16}H_{21}Cl_2N_2O_3$ $[M+H]^+$ 359.0924, found 359.0930.

***Tert*-butyl (*S*)-(2-((3,4-dichlorophenyl)amino)-2-oxo-1-phenylethyl)carbamate (22a)**

According to **GP1**, (*S*)-2-((*tert*-butoxycarbonyl)amino)-3-cyclopropylpropanoic acid (500 mg, 1.99 mmol) was reacted with NMM (547 µL, 5.00 mmol), IBCF (259 µL, 1.99 mmol) and 3,4-dichloroaniline (322 mg, 1.99 mmol). After purification by column chromatography (SiO_2, hexanes/EtOAc 9:1), the corresponding *tert*-butyl (*S*)-(2-((3,4-dichlorophenyl)amino)-2-oxo-1-phenylethyl)carbamate **22a** (680.9 mg, 1.72 mmol, 87%) was obtained as white amorphous solid.

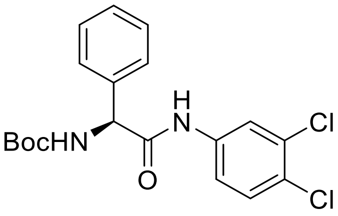

^{1}H NMR (500 MHz, $CDCl_3$) δ: 8.02 (br s, 1H), 7.68 (d, *J* = 2.3 Hz, 1H), 7.34 – 7.46 (m, 5H), 7.29 – 7.33 (m, 1H), 7.23 – 7.26 (m, 1H), 5.66 (br s, 1H), 5.33 (br s, 1H), 1.46 (s, 9H). ^{13}C NMR (126 MHz, $CDCl_3$) δ: 179.6, 155.7, 136.8, 130.4, 129.3, 128.9, 127.4, 121.5, 119.0, 80.9, 59.6, 28.3. HRMS (ESI+) calculated for $C_{19}H_{21}Cl_2N_2O_3$ $[M+H]^+$ 395.0924, found 395.0922.

***Tert*-butyl (*S*)-(1-((3,4-dichlorophenyl)amino)-1-oxo-3-phenylpropan-2-yl)-carbamate (23a)**

According to **GP1**, (*tert*-butoxycarbonyl)-L-phenylalanine (530 mg, 2.00 mmol) was reacted with NMM (550 µL, 5.0 mmol), IBCF (259 µL, 2.0 mmol) and 3,4-dichloroaniline (324 mg, 2.00 mmol). After purification by column chromatography (SiO_2, hexanes/EtOAc 9:1), the corresponding *tert*-butyl (*S*)-(1-((3,4-dichlorophenyl)amino)-1-oxo-3-phenylpropan-2-yl)carbamate **23a** (764.2 mg, 1.87 mmol, 94%) was obtained as white amorphous solid.

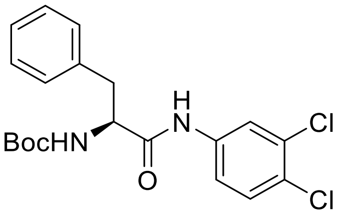

^{1}H NMR (500 MHz, $CDCl_3$) δ: 8.60 (br s, 1H), 7.62 (d, *J* = 2.0 Hz, 1H), 7.20 – 7.34 (m, 6H), 7.12 (dd, *J* = 8.7, 2.4 Hz, 1H), 5.34 (br d, *J* = 7.8 Hz, 1H), 4.55 (br s, 1H), 3.11 – 3.20 (m, 1H), 3.01 – 3.11 (m, 1H), 1.41 ppm (s, 9H). ^{13}C NMR (126 MHz, $CDCl_3$) δ: 170.2, 137.0, 136.3, 132.6, 130.3, 129.2, 128.8, 127.5, 127.2, 121.5, 118.9, 38.3, 28.3. HRMS (ESI+) calculated for $C_{20}H_{23}Cl_2N_2O_3$ $[M+H]^+$ 409.1080, found 409.1076.

***Tert*-butyl (*S*)-(1-((3,4-dichlorophenyl)amino)-1-oxo-3-(pyridin-2-yl)propan-2-yl)carbamate (24a)**

Compound **24a** was prepared according to **GP1a**, using (*S*)-2-((*tert*-butoxycarbonyl)amino)-3-(pyridin-2-yl)propanoic acid (50.0 mg, 0.19 mmol), 3,4-dichloroaniline (30 mg, 0.19 mmol), EDC·HCl (143.7 mg, 0.75 mmol), HOBt·H_2O (114.8 mg, 0.75 mmol), and DIPEA (163.5 uL, 0.94 mmol) in DCM (1.5 mL). After flash column chromatography (SiO_2, hexanes/EtOAc

gradient 100–0 %) (*S*)-(1-((3,4-dichlorophenyl)amino)-1-oxo-3-(pyridin-2-yl)propan-2-yl)carbamate **24a** (71.4 mg, 0.14 mmol, 93%) was obtained as white amorphous solid.

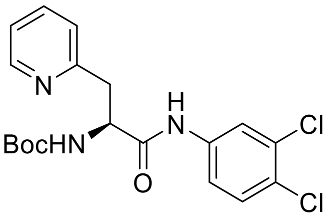

^{1}H NMR (500 MHz, $CDCl_3$) δ: 10.21 (s, 1H), 8.52 (dd, *J* = 5.0, 1.7 Hz, 1H), 7.72 (s, 1H), 7.63 (td, *J* = 7.7, 1.8 Hz, 1H), 7.29 (s, 2H), 7.28 – 7.15 (m, 2H), 6.39 (d, *J* = 6.9 Hz, 1H), 4.71 (s, 1H), 3.30 (d, *J* = 5.7 Hz, 2H), 1.43 (s, 9H). ^{13}C NMR (126 MHz, $CDCl_3$) δ 170.1, 157.8, 155.9, 148.4, 137.6, 137.4, 132.6, 130.4, 127.1, 124.5, 122.1, 121.4, 119.0, 80.3, 54.1, 39.8, 28.3. HRMS (ESI+) calculated for $C_{19}H_{22}Cl_2N_3O_3$ $[M+H]^+$ 410.1033, found 410.1028.

***Tert*-butyl (*S*)-(1-((3,4-dichlorophenyl)amino)-1-oxo-3-(pyridin-3-yl)propan-2-yl)carbamate (25a)**

Compound **25a** was prepared according to **GP1a**, using (*S*)-2-((*tert*-butoxycarbonyl)amino)-3-(pyridin-3-yl)propanoic acid (50.0 mg, 0.19 mmol), 3,4-dichloroaniline (30 mg, 0.19 mmol), EDC·HCl (143.7 mg, 0.75 mmol), HOBt·H_2O (114.8 mg, 0.75 mmol), and DIPEA (163.5 uL, 0.94 mmol) in DCM (1.5 mL). After flash column chromatography (SiO_2, hexanes/EtOAc gradient 100–0 %) (*S*)-(1-((3,4-dichlorophenyl)amino)-1-oxo-3-(pyridin-3-yl)propan-2-yl)carbamate **25a** (57.8 mg, 0.14 mmol, 75%) was obtained as white amorphous solid.

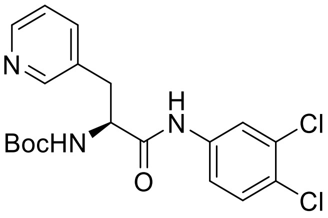

^{1}H NMR (500 MHz, $CDCl_3$) δ: 9.45 (s, 1H), 8.50 – 8.34 (m, 2H), 7.66 (s, 1H), 7.54 (d, *J* = 7.8 Hz, 1H), 7.32 – 7.14 (m, 3H), 5.67 (d, *J* = 8.4 Hz, 1H), 4.56 (q, *J* = 8.4, 7.7 Hz, 1H), 3.08 (ddd, *J* = 66.2, 13.9, 7.2 Hz, 2H), 1.38 (s, 9H). ^{13}C NMR (126 MHz, $CDCl_3$) δ: 169.9, 156.1, 150.2, 148.2, 137.2, 137.1, 132.7, 132.4, 130.4, 127.7, 123.7, 121.5, 119.0, 81.0, 56.2, 35.8, 28.3. HRMS (ESI+) calculated for $C_{19}H_{22}Cl_2N_3O_3$ $[M+H]^+$ 410.1033, found 410.1026.

(1-((2-((3,4-Dichlorophenyl)amino)-2-oxoethyl)amino)-4-methyl-1-oxopentan-2-yl)-phosphonic acid (7)

According to **GP2**, *tert*-butyl (2-((3,4-dichlorophenyl)amino)-2-oxoethyl)carbamate **7a** (102.5 mg, 0.32 mmol) was dissolved in DCM (3.2 mL) and treated at 0 °C with HCl (0.80 mL, 4 M in dioxane). The resulting crystalline hydrochloride was reacted with 2-(diethoxyphosphoryl)-4-methylpentanoic acid **6** (88.8 mg, 0.35 mmol), TBTU (113.02 mg, 0.35 mmol) and NMM (88 µL, 0.80 mmol) in DMF (3.2 mL) to afford the diethyl phosphonate dipeptide (126.0 mg, 0.30 mmol, 93%) without further purification for the next step. The diethyl phosphonate dipeptide (126.0 mg, 0.30 mmol) was deprotected by bromotrimethylsilane (0.26 mL, 1.95 mmol) in DCM (2.8 mL). Then MeOH was added and stirred at room temperature for 30 minutes to cleave the previously formed TMS ester. The solvents were removed under reduced pressure and the crude product was purified *via* a Dionex Ultimate 3000 (Thermo Scientific) with a Nucleodur C18 Graphity column (250mm x 16 mm, particle size 5µm) using UV-detection to afford dipeptide **7** (23.0 mg, 0.058 mmol, 21%) as a colorless solid.

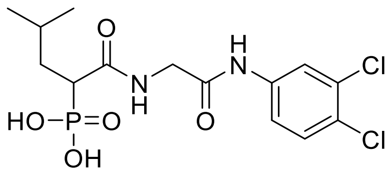

^{1}H NMR (500 MHz, MeOH-d_4) δ = 8.01 (d, *J* = 2.4 Hz, 1H), 7.60 (dd, *J* = 8.8, 2.5 Hz, 1H), 7.46 – 7.40 (m, 1H), 4.22 (d, *J* = 17.2 Hz , 1H), 3.83 (d, *J* = 17.2 Hz, 1H), 3.03 (ddd, *J* = 23.2, 11.4, 2.9 Hz, 1H), 2.08 (tdd, *J* = 9.0, 7.1, 3.6 Hz, 1H), 1.62 (dtd, *J* = 13.2, 6.6, 4.4 Hz, 1H), 1.58 – 1.49 (m, 1H), 0.94 (dd, *J* = 6.5, 3.8 Hz, 7H). ^{13}C NMR (126 MHz, MeOH-d_4) δ = 170.5, 139.5, 133.1, 131.4, 128.1, 123.1, 121.3, 47.5, 46.5, 44.4, 36.3 (d, *J* = 4.7 Hz), 28.10 (d, *J* = 14.5 Hz), 23.5, 21.7. ^{31}P NMR (202 MHz, MeOH-d_4) δ = 22.0. HRMS (ESI+) calculated for $C_{14}H_{20}Cl_2N_2O_5P$ $[M+H]^+$ 397.0481, found 397.0475.

(1-(((*S*)-1-((3,4-Dichlorophenyl)amino)-1-oxopropan-2-yl)amino)-4-methyl-1-oxopentan-2-yl)phosphonic acid (8)

According to **GP2**, *tert*-butyl (*S*)-(1-((3,4-dichlorophenyl)amino)-1-oxopropan-2-yl)carbamate **8a** (100.0 mg, 0.30 mmol) was dissolved in DCM (3.0 mL) and treated at 0 °C with HCl (0.75 mL, 4 M in dioxane). The resulting crystalline hydrochloride was reacted with 2-(diethoxyphosphoryl)-4-methylpentanoic acid **6** (83.3 mg, 0.33 mmol), TBTU (106.0 mg, 0.33 mmol) and NMM (83 µL, 0.75 mmol) in DMF (3.0 mL) to afford the diethyl phosphonate dipeptide (59.3 mg, 0.12 mmol, 40%) without further purification for the next step. The diethyl phosphonate dipeptide (59.3 mg, 0.12 mmol) was deprotected by bromotrimethylsilane (0.11 mL, 0.84 mmol) in DCM (1.2 mL). Then MeOH was added and stirred at room temperature for 30 minutes to cleave the previously formed TMS ester. The solvents were removed under

reduced pressure and the crude product was purified *via* a Waters Autopurifier System (APS) with a Phenomenex Gemini C18 column (250 × 4.6 mm, particle size 5 µm) using mass trigger detection to afford dipeptide **8** (17.7 mg, 40.0 µmol, 36%) as a white amorphous solid.

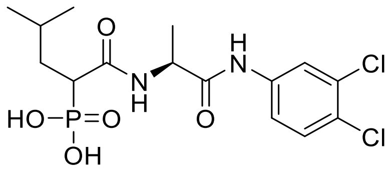

Mixture of diastereomers. Major diastereomer: ^{1}H NMR (500 MHz, MeOH-d_4) δ: 8.03 (d, *J* = 2.3 Hz, 1H), 7.62 (dd, *J* = 8.7, 2.4 Hz, 1H), 7.40 (d, *J* = 9.0 Hz, 1H), 4.49 (q, *J* = 7.2 Hz, 1H), 3.07 (ddd, *J* = 23.8, 11.3, 3.2 Hz, 1H), 1.98 – 2.11 (m, 1H), 1.48 – 1.63 (m, 2H), 1.46 (d, *J* = 7.5 Hz, 3H), 0.90 – 0.96 ppm (m, 6H). ^{13}C NMR (126 MHz, MeOH-d_4) δ: 174.1, 172.1, 139.7, 133.2, 131.4, 128.2, 123.6, 122.8, 121.8, 51.4, 47.4, 46.4, 36.2 (d, *J* = 4.6 Hz), 28.5 (d, *J* = 14.7 Hz), 23.6, 22.0, 17.9. ^{31}P NMR (202 MHz, MeOH-d_4) δ: 22.0. *Minor diastereomer (selected signals):* ^{1}H NMR (500 MHz, MeOH-d_4) δ: 7.92 (d, *J* = 2.3 Hz, 1H), 7.47 (dd, *J* = 8.9, 2.1 Hz, 1H), 4.45 (q, *J* = 7.8 Hz, 1H), 3.00 (ddd, *J* = 19.7, 11.7, 3.1 Hz, 1H), 1.44 (d, *J* = 7.2 Hz, 1H), 0.90 – 0.96 ppm (m, 6H). ^{13}C NMR (126 MHz, MeOH-d_4,) δ: 173.6, 172.2, 139.9, 133.4, 131.7, 128.0, 120.9, 51.7, 47.2, 46.2, 37.3 (d, *J* = 4.6 Hz), 28.4 (d, *J* = 14.7 Hz), 23.8, 21.9, 17.7. ^{31}P NMR (202 MHz, MeOH-d$_4$) δ: 21.9. HRMS (ESI+) calculated for $C_{15}H_{22}Cl_2N_2O_5P$ $[M+H]^+$ 411.0638, found 411.0634.

(1-(((*S*)-1-((3,4-Dichlorophenyl)amino)-3-methyl-1-oxobutan-2-yl)amino)-4-methyl-1-oxopentan-2-yl)phosphonic acid (9).

According **GP2***,* *tert*-butyl (*S*)-(1-((3,4-dichlorophenyl)amino)-3-methyl-1-oxobutan-2-yl)carbamate **9a** (100.0 mg, 0.28 mmol) was dissolved in DCM (2.8 mL) and treated at 0 °C with HCl (0.69 mL, 4 M in dioxane). The resulting crystalline hydrochloride was reacted with 2-(diethoxyphosphoryl)-4-methylpentanoic acid **6** (77.7 mg, 0.31 mmol), TBTU (98.9 mg, 0.31 mmol) and NMM (75 µL, 0.69 mmol) in DMF (2.8 mL) to afford the diethyl phosphonate dipeptide (136.7 mg, 0.28 mmol, quant.) without further purification for the next step. The diethyl phosphonate dipeptide (136.7 mg, 0.28 mmol) was deprotected by bromotrimethylsilane (0.26 mL, 1.93 mmol) in DCM (2.8 mL). Then MeOH was added and stirred at room temperature for 30 minutes to cleave the previously formed TMS ester. The solvents were removed under reduced pressure and the crude product was purified *via* a Waters Autopurifier System (APS) with a Phenomenex Gemini C18 column (250 × 4.6 mm, particle size 5 µm) using mass trigger detection to afford dipeptide **9** (51.7 mg, 0.12 mmol, 43%) as a white amorphous solid.

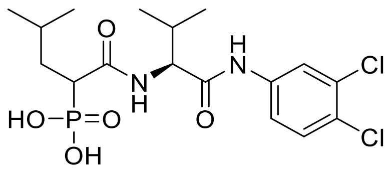

Mixture of diastereomers. Major diastereomer: ^{1}H NMR (500 MHz, MeOH-*d*$_4$) δ: 8.04 (d, *J* = 2.4 Hz, 1H), 7.62 (dd, *J* = 2.4, 8.9 Hz, 1H), 7.42 (d, *J* = 8.9 Hz, 1H), 4.45 (d, *J* = 5.0 Hz, 1H), 3.24 (ddd, *J* = 2.7, 11.6, 23.3 Hz, 1H), 2.42 (qd, *J* = 6.9, 12.1 Hz, 1H), 1.47 – 1.61 (m, 3H), 0.9 – 1.1 (m, 4H), 0.99 (d, *J* = 7.02 Hz, 1H), 0.98 (d, *J* = 6.87 Hz, 3H), 0.95 (d, *J* = 6.56 Hz, 6H). ^{13}C NMR (126 MHz, MeOH-*d*$_4$) δ: 172.7, 172.5 (d, *J* = 4.6 Hz), 139.6, 133.2, 131.5, 128.2, 123.5, 121.6, 60.7, 47.1, 46.0, 36.3 (d, *J* = 4.6 Hz), 31.2, 28.4 (d, *J* = 15.6 Hz), 23.7, 21.8, 19.9, 17.8 ^{31}P NMR (202 MHz, MeOH-d$_4$) δ: 22.7. *Minor diastereomer (selected signals):* ^{1}H NMR (500 MHz, MeOH-*d*$_4$) δ: 7.92 (dd, *J* = 0.61, 1.83 Hz, 1H), 7.44-7.46 (m, 1H), 4.27 (d, *J* = 7.48 Hz, 1H), 3.24 (ddd, *J* = 2.7, 11.9, 22.4 Hz, 1H), 1.95-2.20 (m, 2H), 1.28-1.35 (m, 2H), 1.05 (d, *J* = 6.71 Hz, 3H), 1.00 (d, *J* = 6.71 Hz, 3H), 0.92 (d, *J* = 6.10 Hz, 3H), 0.90 (d, *J* = 6.26 Hz, 3H). ^{13}C NMR (126 MHz, MeOH-*d*$_4$) δ: 172.6, 172.0 (d, *J* = 4.6 Hz), 139.7, 133.5, 131.7, 128.1, 122.8, 120.8, 61.5, 46.8, 45.8, 37.3 (d, *J* = 4.6 Hz), 32.3, 28.1 (d, *J* = 15.6 Hz), 23.8, 21.9, 19.1. ^{31}P NMR (202 MHz, MeOH-*d*$_4$) δ: 22.4. HRMS (ESI+) calculated for $C_{17}H_{26}Cl_2N_2O_5P$ $[M+H]^+$ 439.0956, found 439.0953.

(1-(((*R*)-1-((3,4-Dichlorophenyl)amino)-3-methyl-1-oxobutan-2-yl)amino)-4-methyl-1-oxopentan-2-yl)phosphonic acid (10)

According to **GP2**, *tert*-butyl (*S*)-(1-((3,4-dichlorophenyl)amino)-3-methyl-1-oxobutan-2-yl)carbamate **10a** (100.0 mg, 0.28 mmol) was dissolved in DCM (2.8 mL) and treated at 0 °C with HCl (0.70 mL, 4 M in dioxane). The resulting crystalline hydrochloride was reacted with 2-(diethoxyphosphoryl)-4-methylpentanoic acid **6** (77.7 mg, 0.31 mmol), TBTU (98.9 mg, 0.31 mmol) and NMM (80 µL, 0.70 mmol) in DMF (2.8 mL) to afford the diethyl phosphonate dipeptide (123.4 mg, 0.25 mmol, 89%) without further purification for the next step. The diethyl phosphonate dipeptide (123.4mg, 0.25 mmol) was deprotected by bromotrimethylsilane (0.23 mL, 1.74 mmol) in DCM (2,5 mL). Then MeOH was added and stirred at room temperature for 30 minutes to cleave the previously formed TMS ester. The solvents were removed under reduced pressure and the crude product was purified *via* a Dionex Ultimate 3000 (Thermo Scientific) with a Nucleodur C18 Graphity column (250mm x 16 mm, particle size 5µm) using UV-detection to afford dipeptide **10** (38.0 mg, 0.086 mmol, 35%) as a colorless solid.

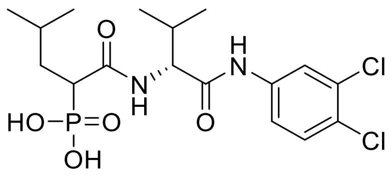

Mixture of diastereomeres. Major diastereomer: ^{1}H NMR (500 MHz, MeOH-d_4) δ: 8.02 (d, *J* = 2.4 Hz, 1H), 7.60 (dd, *J* = 8.8, 2.5 Hz, 1H), 7.45 – 7.39 (m, 1H), 4.45 (d, *J* = 5.2 Hz, 1H), 3.24 (ddd, *J* = 23.5, 11.5, 2.8 Hz, 1H), 2.41 (dd, *J* = 7.0, 5.2 Hz, 1H), 2.12 – 2.05 (m, 1H), 1.54 (tddd, *J* = 19.8, 10.6, 8.0, 5.4 Hz, 2H), 0.99 (dd, *J* = 12.3, 6.9 Hz, 6H), 0.94 (d, *J* = 6.5 Hz, 6H). ^{13}C NMR (126 MHz, MeOH-d_4) δ: 172.5, 139.5, 133.1, 131.3, 128.1, 123.3, 121.5, 60.6, 46.9, 45.9, 36.1 (d, *J* = 4.7 Hz), 31.1, 28.2 (d, *J* = 15.1 Hz), 23.6, 21.7, 19.8, 17.6. ^{31}P NMR (202 MHz, MeOH-d_4) δ: 22.7. *Minor diastereomer (selected signals):* ^{1}H NMR (500 MHz, MeOH-d_4) δ: 7.91 (dd, *J* = 1.9, 0.9 Hz, 1H), 4.27 (d, *J* = 7.6 Hz, 1H), 3.05 (ddd, *J* = 22.6, 11.7, 2.7 Hz, 1H), 2.04 – 1.96 (m, 1H). ^{13}C NMR (126 MHz, MeOH-d_4) δ: 172.4, 139.5, 133.3, 131.6, 127.9, 122.6, 120.7, 61.3, 46.7, 45.7, 37.1, 32.1, 28.0 (d, *J* = 15.0 Hz), 23.6, 21.8, 18.9. ^{31}P NMR (202 MHz, MeOH-d_4) δ: 22.4. HRMS (ESI+) calculated for $C_{17}H_{26}Cl_2N_2O_5P$ $[M+H]^+$ 439.0951, found 439.0946.

(1-(((*R*)-1-((3,4-Dichlorophenyl)amino)-4-methyl-1-oxopentan-3-yl)amino)-4-methyl-1-oxopentan-2-yl)phosphonic acid (11)

According to **GP2**, *tert*-butyl (*R*)-(1-((3,4-dichlorophenyl)amino)-4-methyl-1-oxopentan-3-yl)carbamate **11a** (100.0 mg, 0.27 mmol) was dissolved in DCM (2.7 mL) and treated at 0 °C with HCl (0.66 mL, 4 M in dioxane). The resulting crystalline hydrochloride was reacted with 2-(diethoxyphosphoryl)-4-methylpentanoic acid **6** (74.9 mg, 0.30 mmol), TBTU (95.4 mg, 0.30 mmol) and NMM (74 µL, 0.68 mmol) in DMF (2.7 mL) to afford the diethyl phosphonate dipeptide (102.6 mg, 0.20 mmol, 75%) without further purification for the next step. The diethyl phosphonate dipeptide (102.6 mg, 0.20 mmol) was deprotected by bromotrimethylsilane (0.18 mL, 1.41 mmol) in DCM (2.0 mL). Then MeOH was added and stirred at room temperature for 30 minutes to cleave the previously formed TMS ester. The solvents were removed under reduced pressure and the crude product was purified *via* a Dionex Ultimate 3000 (Thermo Scientific) with a Nucleodur C18 Graphity column (250mm x 16 mm, particle size 5µm) using UV-detection to afford dipeptide **11** (47.0 mg, 0.10 mmol, 50%) as a colorless solid.

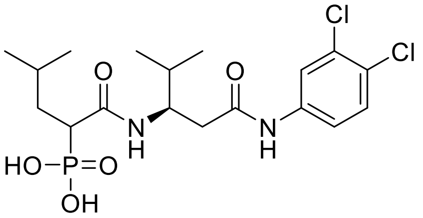

Mixture of diastereomeres. Major diastereomer: ^{1}H NMR (500 MHz, MeOH-d_4) δ: 7.95 (d, *J* = 2.4 Hz, 1H), 7.46 (d, *J* = 2.4 Hz, 1H), 7.42 (d, *J* = 2.0 Hz, 1H), 4.12 (td, *J* = 7.3, 4.9 Hz, 1H), 2.97 – 2.81 (m, 2H), 2.63 (dd, *J* = 8.5, 5.0 Hz, 1H), 2.56 (d, *J* = 7.7 Hz, 1H), 1.87 (dd, *J* = 13.4, 6.7 Hz, 1H), 1.51 (dt, *J* = 18.4, 8.6 Hz, 2H), 1.00 (d, *J* = 6.7 Hz, 6H), 0.86 (dd, *J* = 12.0, 6.1 Hz, 6H). ^{13}C NMR (126 MHz, MeOH-d_4) δ: 172.3, 139.9, 133.2, 131.4, 127.7, 122.8, 120.9, 54.0, 47.4, 46.4, 40.8, 37.1, 32.7, 27.8 (d, *J* = 14.8 Hz), 23.7, 21.4, 20.0, 18.9. ^{31}P NMR (202 MHz, MeOH-d_4) δ: 22.5. *Minor diastereomer (selected signals):* ^{1}H NMR (500 MHz, MeOH-d_4) δ = 7.97 (d, *J* = 2.2 Hz, 1H), 7.48 (d, *J* = 2.4 Hz, 1H), 4.34 – 4.24 (m, 1H), 2.69 – 2.65 (m, 1H), 2.53 (d, *J* = 7.7 Hz, 1H), 2.40 (dd, *J* = 14.3, 10.3 Hz, 1H), 1.98 (d, *J* = 10.8 Hz, 2H), 1.37 (s, 2H), 0.73 (dd, *J* = 6.1, 4.4 Hz, 6H). ^{13}C NMR (126 MHz, MeOH-d_4) δ: 172.2, 140.0, 133.2, 127.5, 122.5, 120.4, 53.7, 46.8, 45.8, 41.2, 37.0, 33.8, 27.3 (d, *J* = 15.0 Hz), 23.9, 21.4, 19.7, 18.7. HRMS (ESI+) calculated for $C_{18}H_{28}Cl_2N_2O_5P$ $[M+H]^+$ 453.1107, found 453.1103.

(1-(((*S*)-1-cyclopropyl-2-((3,4-dichlorophenyl)amino)-2-oxoethyl)amino)-4-methyl-1-oxopentan-2-yl)phosphonic acid (12)

According to **GP2**, *tert*-butyl (*S*)-(1-cyclopropyl-2-((3,4-dichlorophenyl)amino)-2-oxoethyl)carbamate **12a** (115 mg, 0.320 mmol) was dissolved in DCM (3.2 mL) and HCl (4 M in dioxane, 0.80 mL, 3.20 mmol) was added at 0 °C. The resulting crystalline hydrochloride was reacted with 2-(diethoxyphosphoryl)-4-methylpentanoic acid **6** (81.0 mg, 0.32 mmol), TBTU (113 mg, 0.352 mmol) and NMM (157 µL, 0.80 mmol) in DMF (3.2 mL) to afford diethyl (1-(((*S*)-1-cyclopropyl-2-((3,4-dichlorophenyl)amino)-2-oxoethyl)amino)-4-methyl-1-oxopentan-2-yl)phosphonate (165 mg, 0.335 mmol) which was used without further purification and subsequently deprotected with bromotrimethylsilane (0.44 mL, 3.35 mmol) in DCM (3.4 mL). Upon completion of the reaction, MeOH was added and the solvents were removed under reduced pressure and the crude product was purified *via* a Dionex Ultimate 3000 (Thermo Scientific) with a Nucleodur C18 Graphity column (250mm x 16 mm, particle size 5µm) using UV-detection to afford dipeptide **12** (40.0 mg, 0.091 mmol, 34%) as a colorless solid.

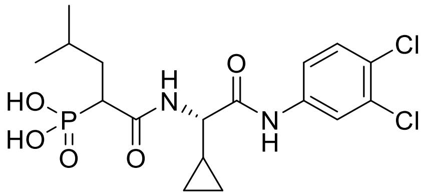

Mixture of diastereomeres. Major diastereomer: ^{1}H NMR (500 MHz, MeOH-d_4) δ: 7.99 – 7.98 (m, 1 H), 7.57 – 7.54 (m, 1 H), 7.40 – 7.37 (m, 1 H), 3.81 (d, J = 9.00 Hz, 1 H), 3.17 – 3.10 (m, 1 H), 2.07 – 2.02 (m, 1 H). 1.58 – 1.50 (m, 2 H), 1.24 – 1.20 (m, 1 H), 0.94 (d, J = 6.41 Hz, 6 H), 0.62 – 0.58 (m, 3 H), 0.37 – 0.33 (m, 1 H). ^{13}C NMR (126 MHz, MeOH-d_4) δ: 127.8, 171.9, 139.6, 133.2, 131.4, 128.2, 123.3, 122.7, 121.5 120.8, 60.2, 47.1, 46.1, 37.3, 36.3, 28.5, 28.3, 23.6, 21.9, 14.4. ^{31}P NMR (203 MHz, MeOH-d_4) δ: 22.3. *Minor diastereomer (selected signals):* ^{1}H NMR (500 MHz, MeOH-d_4) δ: 7.90 – 7.89 (m, 1 H), 3.73 (d, J = 9.00 Hz, 1 H), 3.07 – 3.03 (m, 1 H), 0.91 (d, J = 6.41 Hz, 6 H), 0.45 – 0.41 (m, 1 H). ^{13}C NMR (126 MHz, MeOH-d_4) δ: 127.6, 139.8, 133.4, 131.6, 127.9, 60.6, 46.8, 45.7, 28.0, 27.9, 23.9, 14.2. HRMS (ESI+) calculated for $C_{17}H_{24}Cl_2N_2O_5P$ $[M+H]^+$ 437.0794, found 437.0797.

(1-(((*S*)-1-cyclobutyl-2-((3,4-dichlorophenyl)amino)-2-oxoethyl)amino)-4-methyl-1-oxopentan-2-yl)phosphonic acid (13)

According to **GP2**, *tert*-butyl (*S*)-(1-cyclobutyl-2-((3,4-dichlorophenyl)amino)-2-oxoethyl)carbamate **13a** (75 mg, 0.202 mmol) was dissolved in DCM (3.2 mL) and HCl (4 M in dioxane, 0.51 mL, 2.0 mmol) was added at 0 °C. mmol). The resulting crystalline hydrochloride was reacted with 2-(diethoxyphosphoryl)-4-methylpentanoic acid **6** (51.0 mg, 0.202 mmol), TBTU (71 mg, 0.222 mmol) and NMM (53 µL, 0.8505 mmol) in DMF (2 mL) to afford diethyl (1-(((*S*)-1-cyclobutyl-2-((3,4-dichlorophenyl)amino)-2-oxoethyl)amino)-4-methyl-1-oxopentan-2-yl)phosphonate (80.0 mg, 0.158 mmol) which was used without further purification and subsequently deprotected with bromotrimethylsilane (0.15 mL, 1.14 mmol) in DCM (1 mL). Upon completion of the reaction, MeOH was added and the solvents were removed under reduced pressure and the crude product was purified *via* a Dionex Ultimate 3000 (Thermo Scientific) with a Nucleodur C18 Graphity column (250mm x 16 mm, particle size 5µm) using UV-detection to afford dipeptide **13** (17.7 mg, 0.038 mmol, 38% yield) as a colorless solid.

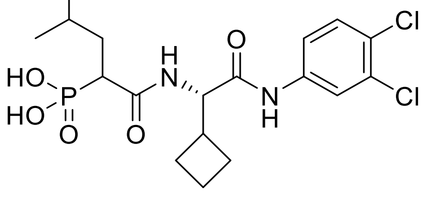

Mixture of diastereomeres. Major diastereomer: ^{1}H NMR (500 MHz, MeOH-d_4) δ: 7.89 (bs, 1 H), 7.45 – 7.41 (m, 2 H), 4.40 (d, *J* = 9.16 Hz, 1 H), 3.07 – 3.00 (m, 1 H), 2.80 – 2.75 (m, 1 H), 2.12 – 2.09 (m, 1 H), 2.05 – 1.98 (m, 4 H), 1.94 – 1.89 (m, 3 H), 1.59 – 1.52 (m, 2 H), 0.92 (d, *J* = 6.10 Hz, 3 H), 0.91 (d, *J* = 6.10 Hz, 3 H). ^{13}C NMR (126 MHz, MeOH-d_4) δ: 171.9, 139.5, 133.3, 131.5, 127.9, 122.6, 120.7, 60.3, 46.8, 45.7, 38.5, 37.1, 27.9, 26.4, 25.9, 23.6, 21.7, 18.9. ^{31}P NMR (203 MHz, MeOH-d_4) δ: 22.2 HRMS (ESI+) calculated for $C_{18}H_{26}Cl_2N_2O_5P$ $[M+H]^+$ 451.0951, found 451.0952.

(1-(((*S*)-1-Cyclohexyl-2-((3,4-dichlorophenyl)amino)-2-oxoethyl)amino)-4-methyl-1-oxopentan-2-yl)phosphonic acid (14)

According to **GP2***,* *tert*-butyl (*S*)-(1-cyclohexyl-2-((3,4-dichlorophenyl)amino)-2-oxoethyl)carbamate **14a** (100.0 mg, 0.25 mmol) was dissolved in DCM (2.5 mL) and treated at 0 °C with HCl (0.62 mL, 4 M in dioxane). The resulting crystalline hydrochloride was reacted with 2-(diethoxyphosphoryl)-4-methylpentanoic acid **6** (69.1 mg, 0.27 mmol), TBTU (86.7 mg, 0.27 mmol) and NMM (69 µL, 0.62 mmol) in DMF (2.5 mL) to afford the diethyl phosphonate dipeptide (60.6 mg, 0.11 mmol, 44%) without further purification for the next step. The diethyl phosphonate dipeptide (60.6 mg, 0.11 mmol) was deprotected by bromotrimethylsilane (0.10 mL, 0.77 mmol) in DCM (1.1 mL). Then MeOH was added and stirred at room temperature for 30 minutes to cleave the previously formed TMS ester. The solvents were removed under reduced pressure and the crude product was purified *via* a Waters Autopurifier System (APS) with a Phenomenex Gemini C18 column (250 × 4.6 mm, particle size 5 µm) using mass trigger detection to afford dipeptide **14** (10.8 mg, 22.0 µmol, 21%) as a white amorphous solid.

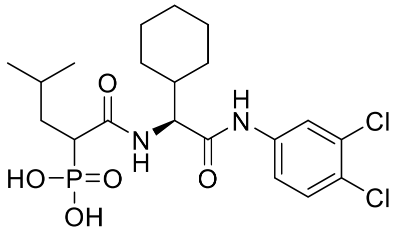

Mixture of diastereomers. Major diastereomer: ^{1}H NMR (500 MHz, MeOH-d_4,) δ: 8.02 (d, *J* = 2.4 Hz, 1H), 7.59 (dd, *J* = 8.7, 2.4 Hz, 1H), 7.41 (d, *J* = 8.9 Hz, 1H), 4.41 (d, *J* = 5.5 Hz, 1H), 3.14 – 3.26 (m, 1H), 1.99 – 2.11 (m, 1H), 1.79 (br d, *J* = 12.7 Hz, 2H), 1.68 (br d, *J* = 9.9 Hz, 2H), 1.48 – 1.64 (m, 2H), 1.16 – 1.38 (m, 8H), 0.94 (d, *J* = 6.4 Hz, 6H). ^{13}C NMR (126 MHz, MeOH-d_4,) δ: 172.7, 139.6, 133.2, 131.4, 128.2, 123.5, 121.7, 60.5, 46.2, 41.1, 36.4 (d, *J* = 3.7 Hz), 31.1, 29.1, 28.3, 27.5, 27.4, 27.3, 23.7, 21.9. ^{31}P NMR (202 MHz, MeOH-d_4) δ: 22.1. *Minor diastereomer (selected signals):* ^{1}H NMR (500 MHz, MeOH-d_4) δ: 7.91 (d, *J* = 2.4 Hz, 1H), 7.43-7.46 (m, 1H), 4.28 (d, *J* = 7.6 Hz, 1H), 2.97 – 3.07 (m, 1H), 1.99 – 2.11 (m, 1H), 1.91 –

1.99 (m, 1H), 1.82 – 1.87 (m, 1H), 1.79 (br d, *J* = 12.7 Hz, 2H), 1.68 (br d, *J* = 9.9 Hz, 2H), 1.48 – 1.64 (m, 2H). ^{13}C NMR (126 MHz, MeOH-*d*$_4$,) δ: 172.6, 131.7, 122.8, 120.9, 117.3, 116.1, 60.9, 47.2, 41.6, 31.0, 30.2, 28.5, 23.8. HRMS (ESI+) calculated for $C_{20}H_{30}Cl_2N_2O_5P$ $[M+H]^+$ 479.1264, found 479.1270.

(1-(((2*S*,3*S*)-1-((3,4-Dichlorophenyl)amino)-3-methyl-1-oxopentan-2-yl)amino)-4-methyl-1-oxopentan-2-yl)phosphonic acid (15)

According to **GP2**, *tert*-butyl ((2*S*,3*S*)-1-((3,4-dichlorophenyl)amino)-3-methyl-1-oxopentan-2-yl)carbamate **15a** (100.0 mg, 0.27 mmol) was dissolved in DCM (2.7 mL) and treated at 0 °C with HCl (0.66 mL, 4 M in dioxane). The resulting crystalline hydrochloride was reacted with 2-(diethoxyphosphoryl)-4-methylpentanoic acid **6** (73.9 mg, 0.29 mmol), TBTU (94.1 mg, 0.29 mmol) and NMM (73 µL, 0.67 mmol) in DMF (2.7 mL) to afford the diethyl phosphonate dipeptide (63.1 mg, 0.12 mmol, 44%) without further purification for the next step. The diethyl phosphonate dipeptide (63.1 mg, 0.12 mmol) was deprotected by bromotrimethylsilane (0.11 mL, 0.84 mmol) in DCM (1.2 mL). Then MeOH was added and stirred at room temperature for 30 minutes to cleave the previously formed TMS ester. The solvents were removed under reduced pressure and the crude product was purified *via* a Waters Autopurifier System (APS) with a Phenomenex Gemini C18 column (250 × 4.6 mm, particle size 5 µm) using mass trigger detection to afford dipeptide **15** (6.8 mg, 15.0 µmol, 13%) as a white amorphous solid.

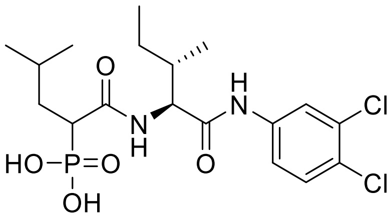

Mixture of diastereomers. Major diastereomer: ^{1}H NMR (500 MHz, MeOH-*d*$_4$) δ: 8.03 (d, *J* = 2.4 Hz, 1H), 7.65 (dd, *J* = 8.8, 2.4 Hz, 1H), 7.41 (d, *J* = 8.9 Hz, 1H), 4.48 (d, *J* = 5.2 Hz, 1H), 3.14 – 3.26 (m, 1H), 1.54 – 1.63 (m, 2H), 1.46 – 1.54 (m, 2H), 1.20 – 1.43 (m, 2H), 0.99 (d, *J* = 6.9 Hz, 3H), 0.87 – 0.97 ppm (m, 9H). ^{13}C NMR (126 MHz, MeOH-*d*$_4$) δ: 172.8, 139.6, 133.2, 131.7, 128.2, 123.6, 121.8, 60.3, 47.2, 36.3 (d, *J* = 3.7 Hz), 28.4 (d, *J* = 15.3 Hz), 25.8, 24.4, 23.7, 21.9, 16.5, 12.2. ^{31}P NMR (202 MHz, MeOH-d$_4$) δ: 21.9. *Minor diastereomer (selected signals):* ^{1}H NMR (500 MHz, MeOH-*d*$_4$) δ: 8.06 (d, *J* = 2.6 Hz, 1H), 7.93 (dd, *J* = 8.0, 2.4 Hz, 1H), 7.61 (dd, *J* = 8.9, 2.4 Hz, 1H), 3.14 – 3.26 (m, 1H), 1.54 – 1.63 (m, 2H), 1.46 – 1.54 (m, 2H), 1.20 – 1.43 (m, 2H), 0.87 – 0.97 (m, 9H). ^{13}C NMR (126 MHz, MeOH-*d*$_4$) δ: 133.4, 131.4, 117.3, 116.0, 37.9, 30.8. HRMS (ESI+) calculated for $C_{18}H_{28}Cl_2N_2O_5P$ $[M+H]^+$ 453.1107, found 453.1104.

(1-(((*S*)-3-Cyclopropyl-1-((3,4-dichlorophenyl)amino)-1-oxopropan-2-yl)amino)-4-methyl-1-oxopentan-2-yl)phosphonic acid (16)

According to **GP2**, *tert*-butyl (*S*)-(3-cyclopropyl-1-((3,4-dichlorophenyl)amino)-1-oxopropan-2-yl)carbamate **16a** (101.5 mg, 0.27 mmol) was dissolved in DCM (2.7 mL) and treated at 0 °C with HCl (0.67 mL, 4 M in dioxane). The resulting crystalline hydrochloride was reacted with 2-(diethoxyphosphoryl)-4-methylpentanoic acid **6** (74.9 mg, 0.30 mmol), TBTU (95.4 mg, 0.30 mmol) and NMM (74 µL, 0.68 mmol) in DMF (2.7 mL) to afford the diethyl phosphonate dipeptide (137.9 mg, 0.27 mmol, quant.) without further purification for the next step. The diethyl phosphonate dipeptide (137.9 mg, 0.27 mmol) was deprotected by bromotrimethylsilane (0.25 mL, 1.90 mmol) in DCM (2.7 mL). Then MeOH was added and stirred at room temperature for 30 minutes to cleave the previously formed TMS ester. The solvents were removed under reduced pressure and the crude product was purified *via* a Dionex Ultimate 3000 (Thermo Scientific) with a Nucleodur C18 Graphity column (250mm x 16 mm, particle size 5µm) using UV-detection to afford dipeptide **16** (17.0 mg, 0.038 mmol, 14%) as a colorless solid.

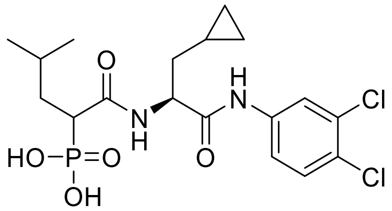

Mixture of diastereomeres. Major diastereomer: ^{1}H NMR (500 MHz, MeOH-d_4) δ: 8.03 (d, *J* = 2.5 Hz, 1H), 7.62 (dd, *J* = 8.7, 2.4 Hz, 1H), 7.42 (dd, *J* = 12.5, 8.8 Hz, 1H), 4.55 (t, *J* = 7.0 Hz, 1H), 3.12 (ddd, *J* = 23.3, 11.4, 2.7 Hz, 1H), 1.76 (t, *J* = 7.1 Hz, 2H), 1.66 – 1.44 (m, 2H), 0.93 (td, *J* = 5.8, 5.3, 3.1 Hz, 8H), 0.47 (dtt, *J* = 26.0, 8.7, 4.6 Hz, 2H), 0.17 (ddq, *J* = 37.4, 9.4, 4.9 Hz, 2H). ^{13}C NMR (126 MHz, MeOH-d_4) δ: 173.1, 139.6, 133.1, 131.3, 128.1, 123.4, 121.6, 56.1, 47.3, 46.3, 37.5, 36.1, 28.3 (d, *J*= 14.8 Hz), 23.5, 21.8, 8.9, 5.6, 4.4. ^{31}P NMR (202 MHz, MeOH-d_4) δ: 22.21 *Minor diastereomer (selected signals):* ^{1}H NMR (500 MHz, MeOH-d_4) δ: 7.93 (d, *J* = 2.4 Hz, 1H), 4.48 (t, *J* = 7.1 Hz, 1H), 3.00 (dd, *J* = 22.1, 11.0 Hz, 1H), 2.18 – 1.98 (m, 3H), 1.86 (dt, *J* = 14.0, 6.9 Hz, 1H), 0.84 (q, *J* = 6.8, 6.1 Hz, 3H). ^{13}C NMR (126 MHz, MeOH-d_4) δ: 172.9, 139.7, 133.3, 131.5, 122.7, 120.8, 56.5, 37.8, 27.9 (d, *J* = 14.9 Hz), 23.7, 21.7, 8.5, 5.1, 4.9. ^{31}P NMR (202 MHz, MeOH-d_4) δ: 22.0. HRMS (ESI+) calculated for $C_{18}H_{26}Cl_2N_2O_5P$ $[M+H]^+$ 451.0951, found 451.0943.

(1-(((*S*)-1-((3,4-Dichlorophenyl)amino)-4-methyl-1-oxopentan-2-yl)amino)-4-methyl-1-oxopentan-2-yl)phosphonic acid (17)

According to **GP2**, *tert*-butyl (*S*)-(1-((3,4-dichlorophenyl)amino)-4-methyl-1-oxopentan-2-yl)-carbamate **17a** (100.0 mg, 0.27 mmol) was dissolved in DCM (2.7 mL) and treated at 0 °C with HCl (0.66 mL, 4 M in dioxane). The resulting crystalline hydrochloride was reacted with 2-(diethoxyphosphoryl)-4-methylpentanoic acid **6** (73.9 mg, 0.29 mmol), TBTU (94.1 mg, 0.29 mmol) and NMM (73 µL, 0.67 mmol) in DMF (2.7 mL) to afford the diethyl phosphonate dipeptide (76.4 mg, 0.15 mmol, 56%) without further purification for the next step. The diethyl phosphonate dipeptide (76.4 mg, 0.15 mmol) was deprotected by bromotrimethylsilane (0.14 mL, 1.05 mmol) in DCM (1.5 mL). Then MeOH was added and stirred at room temperature for 30 minutes to cleave the previously formed TMS ester. The solvents were removed under reduced pressure and the crude product was purified *via* a Waters Autopurifier System (APS) with a Phenomenex Gemini C18 column (250 × 4.6 mm, particle size 5 µm) using mass trigger detection to afford dipeptide **17** (9.1 mg, 20.0 µmol, 13%) as a white amorphous solid.

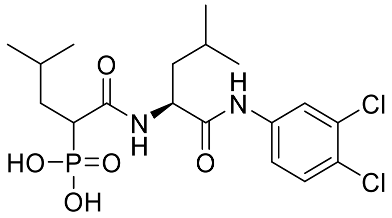

Mixture of diastereomers. Major diastereomer: ^{1}H NMR (500 MHz, MeOH-*d*$_4$) δ: 8.05 (d, *J* = 2.4 Hz, 1H), 7.65 (dd, *J* = 8.9, 2.4 Hz, 1H), 7.40 (d, *J* = 8.9 Hz, 1H), 4.54 (dd, *J* = 11.4, 3.6 Hz, 1H), 3.01 – 3.13 (m, 1H), 2.06-2.15 (m, 1H), 1.77 – 1.84 (m, 1H), 1.74 (dtd, *J* = 16.3, 6.4, 3.7 Hz, 1H), 1.64 – 1.70 (m, 1H), 1.48-1.61 (m, 2H), 0.99 (d, *J* = 6.4 Hz, 3H), 0.86 – 0.97 ppm (m, 9H).

^{13}C NMR (126 MHz, MeOH-*d*$_4$) δ: 174.0, 139.7, 133.1, 131.3, 128.2, 123.8, 122.0, 53.9, 41.4, 36.1 (d, *J* = 3.7 Hz), 28.5 (d, *J* = 14.7 Hz), 26.3, 24.4, 23.8, 23.6, 21.9, 21.3. ^{31}P NMR (202 MHz, MeOH-d$_4$) δ: 21.6. *Minor diastereomer (selected signals):* ^{1}H NMR (500 MHz, MeOH-*d*$_4$) δ: 7.94 (d, *J* = 2.4 Hz, 1H), 7.50 (dd, *J* = 8.9, 2.7 Hz, 1H), 7.42 (d, *J* = 8.9 Hz, 1H), 4.47 (br dd, *J* = 10.0, 5.0 Hz, 1H), 2.93 – 3.01 (m, 1H), 1.99 – 2.05 (m, 1H), 0.86 – 0.97 ppm (m, 9H). ^{13}C NMR (126 MHz, MeOH-*d*$_{4,}$) δ: 131.6, 123.0, 121.1, 28.6, 26.3, 21.9. HRMS (ESI+) calculated for $C_{18}H_{28}Cl_2N_2O_5P$ $[M+H]^+$ 453.1107, found 453.1110.

((*R*)-1-(((S)-1-((3,4-Dichlorophenyl)amino)-3-methoxy-1-oxopropan-2-yl)amino)-4-methyl-1-oxopentan-2-yl)phosphonic acid ((*R*)-18)

((*S*)-1-(((S)-1-((3,4-Dichlorophenyl)amino)-3-methoxy-1-oxopropan-2-yl)amino)-4-methyl-1-oxopentan-2-yl)phosphonic acid ((*S*)-18)

According to **GP2**, *tert*-butyl (*S*)-(1-((3,4-dichlorophenyl)amino)-3-methoxy-1-oxopropan-2-yl)carbamate **18a** (132 mg, 0.36 mmol) was dissolved in DCM (3.6 mL) and treated at 0 °C with HCl (0.90 mL, 4 M in dioxane). The resulting crystalline hydrochloride was reacted with 2-(diethoxyphosphoryl)-4-methylpentanoic acid **6** (101 mg, 0.40 mmol), TBTU (128 mg, 0.40 mmol) and NMM (100 µL, 0.90 mmol) in DMF (3.6 mL). After purification by column chromatography (SiO_2, PB/EtOAc 5:1 to 2:1), a separation of the diastereomeres (70 mg, 0.14 mmol, 39%) and (109 mg, 0.22 mmol, 61%) was achieved. The diastereomers were deprotected separately.

(*R/S*)-Diethyl phosphonate dipeptide (70 mg, 0.14 mmol) was deprotected by bromotrimethylsilane (0.13 mL, 0.98 mmol) in DCM (1.4 mL). Then MeOH was added and stirred at room temperature for 30 minutes to cleave the previously formed TMS ester. The solvents were removed under reduced pressure and the crude product was purified *via* a Dionex Ultimate 3000 (Thermo Scientific) with a Nucleodur C18 Graphity column (250mm x 16 mm, particle size 5µm) using UV-detection to afford dipeptide **(*R*)-18** (9.8 mg, 0.022 mmol, 16%) as a colorless solid.

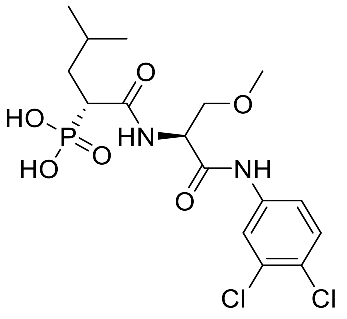

(*R*)-18: ^{1}H NMR (500 MHz, MeOH-*d*$_4$) δ: 8.05 (d, *J* = 2.4 Hz, 1H), 7.65 (dd, *J* = 8.8 Hz, 2.4, 1H), 7.41 (d, *J* = 8.8 Hz, 1H), 4.65 (dd, *J* = 5.0, 3.5 Hz, 1H), 3.91 (dd, *J* = 9.7, 5.0 Hz, 1H), 3.66 (dd, *J* = 9.7, 3.6 Hz, 1H), 3.37 (s, 3H), 3.23 (ddd, *J* = 23.8, 11.2, 2.7 Hz, 1H), 2.08 (dtd, *J* = 16.1, 7.1, 3.5 Hz, 1H), 1.65 – 1.47 (m, 2H), 0.94 (dd, *J* = 6.5, 1.3 Hz, 6H). ^{13}C NMR (126 MHz, MeOH-*d*$_4$) δ: 171.2, 139.4, 133.0, 131.2, 128.1, 123.5, 121.7, 72.9, 59.3, 55.8, 46.9, 45.9, 35.9 (d, *J* = 4.8 Hz), 28.3 (d, *J* = 14.7 Hz), 23.4, 22.0. ^{31}P NMR (202 MHz, MeOH-*d*$_4$) δ: 22.4. HRMS (ESI+) calculated for $C_{16}H_{24}Cl_2N_2O_6P$ $[M+H]^+$ 441.0744, found 441.0737.

The (*S*/*S*)-diethyl phosphonate dipeptide (107 mg, 0.21 mmol) was deprotected by bromotrimethylsilane (0.19 mL, 1.47 mmol) in DCM (2.1 mL). Then MeOH was added and stirred at room temperature for 30 minutes to cleave the previously formed TMS ester. The solvents were removed under reduced pressure and the crude product was purified *via* a Dionex Ultimate 3000 (Thermo Scientific) with a Nucleodur C18 Graphity column (250mm x 16 mm, particle size 5µm) using UV-detection to afford dipeptide **(*S*)-18** (22.8 mg, 0.052 mmol, 25%) as a colorless solid.

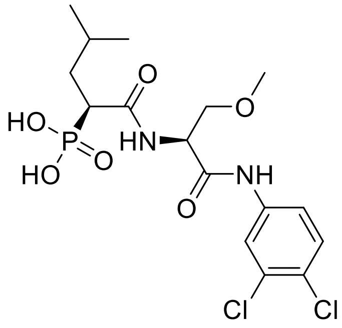

(*S*)-18: ^{1}H NMR (500 MHz, MeOH-d_4) δ: 7.94 (d, *J* = 2.4 Hz, 1H), 7.42 (dd, *J* = 13.4, 8.8 Hz, 2H), 4.69 – 4.60 (m, 1H), 3.77 (dd, *J* = 9.7, 5.5 Hz, 1H), 3.73 – 3.63 (m, 1H), 3.37 (s, 3H), 3.04 (ddd, *J* = 22.2, 11.5, 3.1 Hz, 1H), 2.00 (dtd, *J* = 12.2, 8.4, 4.0 Hz, 1H), 1.65 – 1.58 (m, 2H), 0.97 – 0.92 (m, 6H). ^{13}C NMR (126 MHz, MeOH-d_4) δ: 170.7, 139.5, 133.3, 131.5, 128.0, 122.7, 120.8, 72.7, 59.4, 55.7, 47.2, 46.21 37.2 (d, *J* = 4.5 Hz), 27.9 (d, *J* = 14.9 Hz), 23.6, 21.7. ^{31}P NMR (202 MHz, MeOH-d_4) δ: 22.0.

(1-(((*S*)-3-Cyclohexyl-1-((3,4-dichlorophenyl)amino)-1-oxopropan-2-yl)amino)-4-methyl-1-oxopentan-2-yl)phosphonic acid (19)

According to **GP2***, tert*-butyl (*S*)-(3-cyclohexyl-1-((3,4-dichlorophenyl)amino)-1-oxopropan-2-yl)carbamate **19a** (100.0 mg, 0.24 mmol) was dissolved in DCM (2.4 mL) and treated at 0 °C with HCl (0.60 mL, 4 M in dioxane). The resulting crystalline hydrochloride was reacted with 2-(diethoxyphosphoryl)-4-methylpentanoic acid **6** (66.6 mg, 0.26 mmol), TBTU (84.8 mg, 0.26 mmol) and NMM (67 µL, 0.61 mmol) in DMF (2.4 mL) to afford the diethyl phosphonate dipeptide (112.5 mg, 0.19 mmol, 79%) without further purification for the next step. Diethyl phosphonate dipeptide (112.5 mg, 0.19 mmol) was deprotected by bromotrimethylsilane (0.17 mL, 1.32 mmol) in DCM (1.9 mL). Then MeOH was added and stirred at room temperature for 30 minutes to cleave the previously formed TMS ester. The solvents were removed under reduced pressure and the crude product was purified *via* a Waters Autopurifier System (APS)

with a Phenomenex Gemini C18 column (250 × 4.6 mm, particle size 5 μm) using mass trigger detection to afford dipeptide **19** (92.4 mg, 0.19 mmol, quant.) as a white amorphous solid.

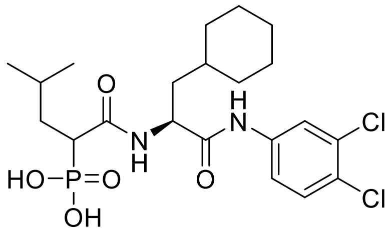

Mixture of diastereomers. Major diastereomer: ^{1}H NMR (500 MHz, MeOH-*d*$_4$) δ: 7.93 (d, *J* = 2.4 Hz, 1H), 7.47 – 7.51 (m, 1H), 7.38-7.44 (m, 1H), 4.51 (dd, *J* = 9.8, 5.6 Hz, 1H), 3.00 (ddd, *J* = 22.3, 12.5, 2.7 Hz, 1H), 1.97 – 2.06 (m, 1H), 1.78 – 1.94 (m, 2H), 1.53 – 1.76 (m, 11H), 1.45 – 1.52 (m, 1H), 1.15 – 1.34 (m, 1H), 0.89 – 0.98 (m, 6H). ^{13}C NMR (126 MHz, MeOH-*d*$_4$) δ: 173.7, 139.8, 133.4, 131.6, 128.0, 123.7, 122.9, 121.0, 53.7, 47.5, 40.1, 37.3 (d, *J* = 4.6 Hz), 35.4, 35.2, 33.3, 28.1 (d, *J* = 15.6 Hz), 27.7, 27.3, 24.4, 23.9, 21.8. ^{31}P NMR (202 MHz, MeOH-*d*$_4$) δ: 22.0. *Minor diastereomer (selected signals):* ^{1}H NMR (500 MHz, MeOH-*d*$_4$) δ: 8.04 (d, *J* = 2.4 Hz, 1H), 7.63 (dd, *J* = 8.9, 2.4 Hz, 1H), 4.60 (dd, *J* = 11.8, 3.7 Hz, 1H), 3.10 (ddd, *J* = 23.8, 11.3, 2.6 Hz, 1H), 2.07 – 2.16 (m, 1H), 1.15 – 1.34 (m, 5H), 0.98 – 1.08 (m, 2H), 0.89 – 0.98 (m, 6H). ^{13}C NMR (126 MHz, MeOH-*d*$_4$) δ: 174.0, 139.7, 133.2, 131.4, 128.2, 121.9, 52.9, 46.5, 39.7, 35.9 (d, *J* = 4.6 Hz) 35.8, 35.3, 32.7, 28.5 (d, *J* = 15.6 Hz), 27.8, 27.6, 27.4, 23.7, 21.9. ^{31}P NMR (202 MHz, MeOH-*d*$_4$) δ: 22.2. HRMS (ESI+) calculated for $C_{21}H_{32}Cl_2N_2O_5P$ $[M+H]^+$ 493.1420, found 493.1425.

(1-(((*S*)-1-((3,4-Dichlorophenyl)amino)-1-oxo-3-(tetrahydro-2H-pyran-4-yl)propan-2-yl)amino)-4-methyl-1-oxopentan-2-yl)phosphonic acid (20)

According to **GP2a***,* *tert*-butyl (*S*)-(1-((3,4-dichlorophenyl)amino)-1-oxo-3-(tetrahydro-2H-pyran-4-yl)propan-2-yl)carbamate **20a** (55.0 mg, 0.13 mmol) was dissolved in DCM (1 mL) and treated at 0 °C with HCl (0.5 mL, 4 M in dioxane). The resulting hydrochloride salt was reacted with 2-(diethoxyphosphoryl)-4-methylpentanoic acid **6** (50.7 mg, 0.20 mmol), EDC·HCl (51.4 mg, 0.27 mmol), HOBt·H_2O (41.0 mg, 0.27 mmol), and DIPEA (56.0 uL, 0.31 mmol) in DMF (2.0 mL) to afford the diethyl phosphonate dipeptide, which was treated with bromotrimethylsilane (0.48 mL, 3.64 mmol) in DCM (1.0 mL). After completion of reaction MeOH was added and stirred at room temperature for 30 minutes to cleave the TMS ester. The volatiles were removed under reduced pressure and the crude product was purified *via* preparative HPLC to obtain mixture of diastereomers (7.2 mg, 0.014 mmol, 11%) as a colorless oil.

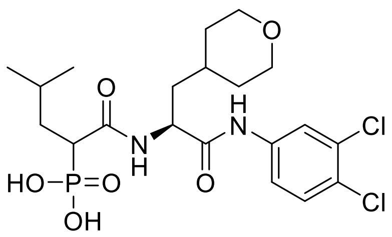

^{1}H NMR (500 MHz, DMSO-d_6) δ: 10.34 (s, 1H), 8.71 (d, *J* = 8.5 Hz, 1H), 8.18 (d, *J* = 2.4 Hz, 1H), 7.83 (dd, *J* = 8.9, 2.5 Hz, 1H), 7.56 (d, *J* = 8.9 Hz, 1H), 4.48 (ddd, *J* = 11.9, 8.7, 3.7 Hz, 1H), 4.06 – 3.90 (m, 2H), 3.90 – 3.74 (m, 2H), 3.20 (t, *J* = 11.4 Hz, 1H), 3.14 – 3.04 (m, 1H), 2.02 – 1.89 (m, 1H), 1.83 (dq, *J* = 11.1, 5.9, 4.5 Hz, 1H), 1.68 – 1.41 (m, 5H), 1.35 – 1.12 (m, 2H), 0.86 (dd, *J* = 6.4, 3.1 Hz, 6H). ^{13}C NMR (126 MHz, DMSO-d_6) δ: 171.7, 167.1, 139.0, 130.8, 130.5, 125.0, 121.0, 119.8, 67.09 (d, *J* = 27.5 Hz), 61.4 (d, *J* = 5.4 Hz), 50.2, 37.4, 34.1, 33.1, 31.3 (d, *J* = 32.8 Hz), 26.8 (d, *J* = 15.2 Hz), 22.8, 21.2, 16.4 (d, *J* = 5.5 Hz). ^{31}P NMR (202 MHz, DMSO) δ: 22.1. HRMS (ESI+) calculated for $C_{20}H_{30}Cl_2N_2O_6P$ $[M+H]^+$ 495.1213, found 495.1206.

(1-((*S*)-2-((3,4-Dichlorophenyl)carbamoyl)pyrrolidin-1-yl)-4-methyl-1-oxopentan-2-yl)-phosphonic acid (21)

According to **GP2**, *tert*-butyl (*S*)-2-((3,4-dichlorophenyl)carbamoyl)pyrrolidine-1-carboxylate **21a** (101.5 mg, 0.28 mmol) was dissolved in DCM (2.8 mL) and treated at 0 °C with HCl (0.70 mL, 4 M in dioxane). The resulting crystalline hydrochloride was reacted with 2-(diethoxyphosphoryl)-4-methylpentanoic acid **6** (77.7 mg, 0.31 mmol), TBTU (98.9 mg, 0.31 mmol) and NMM (80 µL, 0.70 mmol) in DMF (2.8 mL) to afford the diethyl phosphonate dipeptide (122.7 mg, 0.25 mmol, 89%) without further purification for the next step. Diethyl phosphonate dipeptide (122.7 mg, 0.25 mmol) was deprotected by bromotrimethylsilane (0.23 mL, 1.78 mmol) in DCM (2.5 mL). Then MeOH was added and stirred at room temperature for 30 minutes to cleave the previously formed TMS ester. The solvents were removed under reduced pressure and the crude product was purified *via* a Dionex Ultimate 3000 (Thermo Scientific) with a Nucleodur C18 Graphity column (250mm x 16 mm, particle size 5µm) using UV-detection to afford dipeptide **21** (60.0 mg, 0.14 mmol, 55% yield) as a colorless solid.

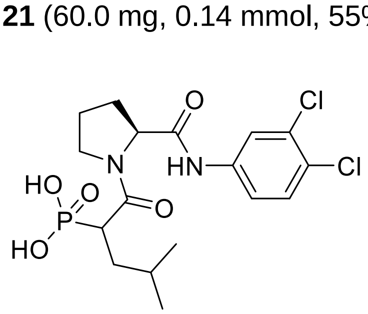

Mixture of diastereomeres. Major diastereomer: ^{1}H NMR (500 MHz, MeOH-d_4) δ: 8.03 (d, J = 2.4 Hz, 1H), 7.88 (s, J = 1.3 Hz, 1H), 7.42 (d, J = 1.5 Hz, 1H), 4.54 (dd, J = 8.7, 4.7 Hz, 1H), 3.98 (dt, J = 9.6, 6.5 Hz, 1H), 3.78 – 3.64 (m, 2H), 2.39 – 1.88 (m, 6H), 1.70 – 1.45 (m, 1H), 0.92 (d, J = 6.3 Hz, 6H). ^{13}C NMR (126 MHz, MeOH-d_4) δ: 172.8, 171.5, 139.9, 133.3, 131.5, 127.7, 122.4, 120.5, 62.6, 44.7, 43.6, 37.7 (d, J = 4.3 Hz), 30.8, 27.5 (d, J = 14.9 Hz), 25.8, 23.8, 22.0. ^{31}P NMR (202 MHz, MeOH-d_4) δ: 21.4. *Minor diastereomer (selected signals):* ^{1}H NMR (500 MHz, MeOH-d_4) δ: 7.64 (d, J = 2.5 Hz, 1H), 7.62 (d, J = 2.4 Hz, 1H), 7.39 (s, 1H), 4.62 (dd, J = 8.8, 3.0 Hz, 1H), 4.09 (ddd, J = 9.6, 7.9, 3.6 Hz, 1H), 0.95 (d, J = 6.5 Hz, 6H). ^{13}C NMR (126 MHz, MeOH-d_4) δ: 173.3, 139.4, 133.0, 131.2, 128.1, 123.5, 121.7, 45.4, 44.4, 36.5 (d, J = 4.8 Hz), 31.0, 28.3 (d, J = 14.4 Hz), 25.4, 23.4, 22.1. ^{31}P NMR (202 MHz, MeOH-d_4) δ: 20.8. HRMS (ESI+) calculated for $C_{17}H_{24}Cl_2N_2O_5P$ [M+H]$^+$ 437.0794, found 437.0789.

(1-(((*S*)-2-((3,4-Dichlorophenyl)amino)-2-oxo-1-phenylethyl)amino)-4-methyl-1-oxopentan-2-yl)phosphonic acid (22)

According to **GP2**, *tert*-butyl (*S*)-(2-((3,4-dichlorophenyl)amino)-2-oxo-1-phenylethyl)carbamate **22a** (102.9 mg, 0.26 mmol) was dissolved in DCM (2.6 mL) and treated at 0 °C with HCl (0.64 mL, 4 M in dioxane). The resulting crystalline hydrochloride was reacted with 2-(diethoxyphosphoryl)-4-methylpentanoic acid **6** (72.1 mg, 0.29 mmol), TBTU (91.8 mg, 0.29 mmol) and NMM (71 µL, 0.65 mmol) in DMF (2.7 mL) to afford the diethyl phosphonate dipeptide (135.6 mg, 0.26 mmol, quant.) without further purification for the next step. Diethyl phosphonate dipeptide (135.6 mg, 0.26 mmol) was deprotected by bromotrimethylsilane (0.24 mL, 1.78 mmol) in DCM (2.6 mL). Then MeOH was added and stirred at room temperature for 30 minutes to cleave the previously formed TMS ester. The solvents were removed under reduced pressure and the crude product was purified *via* a Dionex Ultimate 3000 (Thermo Scientific) with a Nucleodur C18 Graphity column (250mm x 16 mm, particle size 5µm) using UV-detection to afford dipeptide **22** (48.3 mg, 0.10 mmol, 38% yield) as a colorless solid

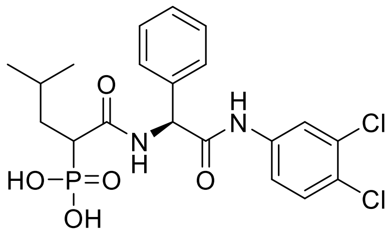

Mixture of diastereomeres. Major diastereomer: ^{1}H NMR (500 MHz, MeOH-d_4) δ: 8.00 (d, J = 2.4 Hz, 1H), 7.59 (dd, J = 8.8, 2.5 Hz, 1H), 7.47 – 7.34 (m, 6H), 5.55 (s, 1H), 3.22 – 3.12 (m, 1H), 2.06 (s, 1H), 1.59 – 1.46 (m, 2H), 0.88 (t, J = 6.6 Hz, 6H). ^{13}C NMR (126 MHz, MeOH-d_4) δ: = 171.5, 139.5, 138.3, 133.2, 131.4, 130.0, 129.6, 129.1, 128.3, 123.3, 121.5, 60.2, 46.9,

36.3, 28.2 (d, *J* = 14.7 Hz), 23.5, 21.8. ^{31}P NMR (202 MHz, MeOH-*d*$_4$) δ: 22.3. HRMS (ESI+) calculated for $C_{20}H_{24}Cl_2N_2O_5P$ $[M+H]^+$ 473.0794, found 473.0788.

(1-(((*S*)-1-((3,4-Dichlorophenyl)amino)-1-oxo-3-phenylpropan-2-yl)amino)-4-methyl-1-oxopentan-2-yl)phosphonic acid (23)

According to **GP2**, *tert*-butyl (*S*)-(1-((3,4-dichlorophenyl)amino)-1-oxo-3-phenylpropan-2-yl)carbamate **23a** (100.0 mg, 0.24 mmol) was dissolved in DCM (2.4 mL) and treated at 0 °C with HCl (0.61 mL, 4 M in dioxane). The resulting crystalline hydrochloride was reacted with 2-(diethoxyphosphoryl)-4-methylpentanoic acid **6** (67.8 mg, 0.27 mmol), TBTU (86.2 mg, 0.27 mmol) and NMM (67 µL, 0.61 mmol) in DMF (2.4 mL) to afford the diethyl phosphonate dipeptide (130.4 mg, 0.24 mmol, quant.) without further purification for the next step. The diethyl phosphonate dipeptide (116.4 mg, 0.21 mmol) was deprotected by bromotrimethylsilane (0.19 mL, 1.47 mmol) in DCM (2.1 mL). Then MeOH was added and stirred at room temperature for 30 minutes to cleave the previously formed TMS ester. The solvents were removed under reduced pressure and the crude product was purified *via* a Waters Autopurifier System (APS) with a Phenomenex Gemini C18 column (250 × 4.6 mm, particle size 5 µm) using mass trigger detection to afford dipeptide **23** (44.1 mg, 90.0 µmol, 43%) as a white amorphous solid.

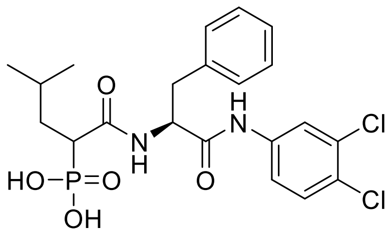

Mixture of diastereomers. Major diastereomer: ^{1}H NMR (500 MHz, MeOH-*d*$_4$) δ: 7.86 (d, *J* = 1.4 Hz, 1H), 7.38 – 7.41 (m, 1H), 7.24 – 7.29 (m, 5H), 7.16 (d, *J* = 8.7 Hz, 1H), 6.83 (d, *J* = 2.6 Hz, 1H), 4.63 – 4.68 (m, 1H), 3.24 (dd, *J* = 14.2, 7.0 Hz, 1H), 3.09 (dd, *J* = 13.6, 8.4 Hz, 1H), 2.89 – 2.98 (m, 1H), 1.88 – 1.98 (m, 2H), 1.49 (dtd, *J* = 13.5, 9.9, 3.3 Hz, 1H), 0.84 (dd, *J* = 6.6, 1.5 Hz, 6H). ^{13}C NMR (126 MHz, MeOH-*d*$_4$,) δ: 172.2, 139.6, 138.5, 133.3, 131.8, 131.5, 130.5, 129.7, 128.0, 123.1, 121.2, 117.5, 57.6, 47.6, 38.5, 37.3 (d, *J* = 4.6 Hz), 27.8 (d, *J* = 15.3 Hz), 24.6, 23.9, 21.6. ^{31}P NMR (202 MHz, MeOH-d$_4$) δ: 21.9. *Minor diastereomer (selected signals):* ^{1}H NMR (500 MHz, MeOH-*d*$_4$) δ: 8.05 (d, *J* = 2.4 Hz, 1H), 7.64 (dd, *J* = 8.8, 2.5 Hz, 1H), 7.38 – 7.41 (m, 1H), 6.60 (dd, *J* = 8.6, 2.7 Hz, 1H), 3.48 (dd, *J* = 14.2, 4.1 Hz, 1H), 2.83 (dd, *J* = 14.1, 11.2 Hz, 1H), 1.28 – 1.44 (m, 2H), 0.73 (d, *J* = 6.7 Hz, 3H), 0.70 (d, *J* = 6.6 Hz, 3H). ^{13}C NMR (126 MHz, MeOH-*d*$_4$,) δ: 172.5, 139.1, 133.2, 131.6, 131.4, 130.3, 129.6, 127.8, 123.6, 121.8, 116.3, 56.5, 46.6, 38.4, 36.0 (d, *J* = 4.6 Hz), 27.8 (d, *J* = 15.3 Hz), 24.3,

23.7, 21.8. ^{31}P NMR (202 MHz, MeOH-d$_4$) δ: 22.4. HRMS (ESI+) calculated for $C_{21}H_{26}Cl_2N_2O_5P$ $[M+H]^+$ 487.0951, found 487.0955.

((*R*)-1-(((*S*)-1-((3,4-Dichlorophenyl)amino)-1-oxo-3-(pyridin-2-yl)propan-2-yl)amino)-4-methyl-1-oxopentan-2-yl)phosphonic acid ((*R*)-24)

((*S*)-1-(((*S*)-1-((3,4-Dichlorophenyl)amino)-1-oxo-3-(pyridin-2-yl)propan-2-yl)amino)-4-methyl-1-oxopentan-2-yl)phosphonic acid ((*S*)-24)

According to **GP2a**, tert-butyl (*S*)-(1-((3,4-dichlorophenyl)amino)-1-oxo-3-(pyridin-2-yl)propan-2-yl)carbamate **24a** (60.0 mg, 0.15 mmol) was dissolved in DCM (1 mL) and treated at 0 °C with HCl (0.5 mL, 4 M in dioxane). The resulting hydrochloride salt (approx. 0.13 mmol, 1 equiv.) was reacted with 2-(diethoxyphosphoryl)-4-methylpentanoic acid **6** (48.8 mg, 0.193 mmol), EDC·HCl (49.4 mg, 0.26 mmol), HOBt·H_2O (39.5 mg, 0.26 mmol), and DIPEA (53.9 uL, 0.31 mmol) in DMF (2.0 mL) to afford the diethyl phosphonate dipeptide, which was treated with bromotrimethylsilane (0.12 mL, 0.91 mmol) in DCM (1.0 mL). After completion of reaction MeOH was added and stirred at room temperature for 30 minutes to cleave the TMS ester. The volatiles were removed under reduced pressure and the crude product was purified *via* preparative HPLC to obtain two dipeptide diastereomeres **(*R*)-24** (10.0 mg, 0.020 mmol, 15%) and **(*S*)-24** (15.0 mg, 0.031 mmol, 20%) as amorphous solids.

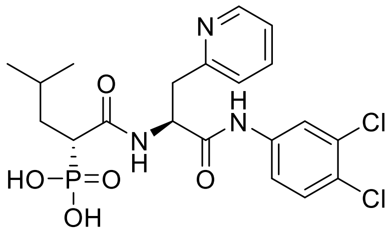

(*R*)-24: ^{1}H NMR (500 MHz, DMSO-*d*$_6$) δ: 10.31 (s, 1H), 8.62 (d, *J* = 9.0 Hz, 1H), 8.33 (d, *J* = 4.8 Hz, 1H), 8.06 (d, *J* = 2.4 Hz, 1H), 7.74 (dd, *J* = 8.9, 2.4 Hz, 1H), 7.60 – 7.53 (m, 1H), 7.41 (d, *J* = 8.8 Hz, 1H), 7.19 (d, *J* = 7.8 Hz, 1H), 7.10 (dd, *J* = 7.5, 4.9 Hz, 1H), 4.79 (ddd, *J* = 12.0, 8.9, 3.5 Hz, 1H), 3.38 (dd, *J* = 13.8, 3.5 Hz, 1H), 2.84 (dd, *J* = 13.9, 11.6 Hz, 1H), 2.67 (ddd, *J* = 23.3, 11.6, 2.5 Hz, 1H), 1.62 (ddd, *J* = 13.5, 11.2, 4.9 Hz, 1H), 1.12 – 1.01 (m, 1H), 0.86 – 0.77 (m, 1H), 0.45 (d, *J* = 6.9 Hz, 6H). ^{13}C NMR (126 MHz, DMSO-*d*$_6$) δ: 171.3, 169.4 (d, *J* = 5.1 Hz), 158.0, 148.9, 139.4, 137.2, 131.2, 130.8, 125.5, 124.6, 122.2, 121.7, 120.5, 53.6, 45.8 (d, *J* = 122.3 Hz), 39.1, 34.6 (d, *J* = 4.8 Hz), 26.8 (d, *J* = 15.4 Hz), 23.2, 21.6. ^{31}P NMR (202 MHz, DMSO) δ: 21.3. HRMS (ESI+) calculated for $C_{20}H_{25}Cl_2N_3O_5P$ $[M+H]^+$ 488.0903, found 488.0899.

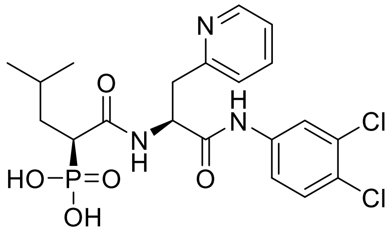

(*S*)-24: ^{1}H NMR (500 MHz, DMSO-d_6) δ: 10.33 (s, 1H), 8.49 (d, *J* = 4.4 Hz, 1H), 8.35 (d, *J* = 8.0 Hz, 1H), 8.08 (d, *J* = 2.4 Hz, 1H), 7.82 – 7.64 (m, 2H), 7.53 (d, *J* = 8.8 Hz, 1H), 7.34 (d, *J* = 7.8 Hz, 1H), 7.28 (dd, *J* = 7.6, 5.0 Hz, 1H), 4.77 (td, *J* = 8.6, 4.9 Hz, 1H), 3.43 – 3.22 (m, 2H), 2.76 (ddd, *J* = 22.2, 12.1, 3.4 Hz, 1H), 1.91 – 1.75 (m, 1H), 1.39 – 1.26 (m, 1H), 1.22 – 1.09 (m, 1H), 0.70 (dd, *J* = 30.3, 6.5 Hz, 6H). ^{13}C NMR (126 MHz, DMSO-d_6) δ: 170.4, 169,4 157.4, 148.2, 139.1, 137.3, 130.7, 130.4, 124.8, 124.4, 122.0, 121.0, 119.8, 54.1, 46.2 (d, *J* = 121.6 Hz), 38.0, 35.3 (d, *J* = 3.4 Hz), 26.0 (d, *J* = 14.7 Hz), 23.3, 21.0. ^{31}P NMR (202 MHz, DMSO) δ: 21.8. HRMS (ESI+) calculated for $C_{20}H_{25}Cl_2N_3O_5P$ $[M+H]^+$ 488.0903, found 488.0901.

((*R*)-1-(((*S*)-1-((3,4-Dichlorophenyl)amino)-1-oxo-3-(pyridin-3-yl)propan-2-yl)amino)-4-methyl-1-oxopentan-2-yl)phosphonic acid ((*R*)-25)

((*S*)-1-(((*S*)-1-((3,4-Dichlorophenyl)amino)-1-oxo-3-(pyridin-3-yl)propan-2-yl)amino)-4-methyl-1-oxopentan-2-yl)phosphonic acid ((*S*)-25)

According to **GP2a***, tert*-butyl (*S*)-(1-((3,4-dichlorophenyl)amino)-1-oxo-3-(pyridin-3-yl)propan-2-yl)carbamate **25a** (55.0 mg, 0.13 mmol) was dissolved in DCM (1 mL) and treated at 0 °C with HCl (0.5 mL, 4 M in dioxane). The resulting hydrochloride salt was reacted with 2-(diethoxyphosphoryl)-4-methylpentanoic acid **6** (47.6 mg, 0.0.19 mmol), EDC·HCl (48.2 mg, 0.25 mmol), HOBt·H_2O (38.5 mg, 0.25 mmol), and DIPEA (52.6 uL, 0.30 mmol) in DMF (2.0 mL) to afford the diethyl phosphonate dipeptide, which was treated with bromotrimethylsilane (0.12 mL, 0.91 mmol) in DCM (1.0 mL). After completion of reaction MeOH was added and stirred at room temperature for 30 minutes to cleave the TMS ester. The volatiles were removed under reduced pressure and the crude product was purified *via* preparative HPLC to obtain two dipeptide diastereomeres **(*R*)-25** (10 mg, 0.020 mmol, 15%) and **(*S*)-25** (7.0 mg, 0.014 mmol, 11%) as colorless solids.

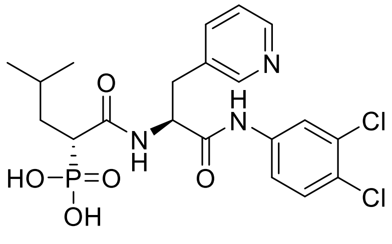

(*R*)-25: ^{1}H NMR (500 MHz, DMSO-d_6) δ: 10.49 (s, 1H), 8.83 (d, *J* = 9.2 Hz, 1H), 8.50 (s, 1H), 8.42 (d, *J* = 4.9 Hz, 1H), 8.21 (d, *J* = 2.4 Hz, 1H), 7.90 (dd, *J* = 8.9, 2.5 Hz, 1H), 7.76 (d, *J* = 7.9 Hz, 1H), 7.58 (d, *J* = 8.9 Hz, 1H), 7.31 (dd, *J* = 7.8, 4.8 Hz, 1H), 4.74 (ddd, *J* = 12.6, 9.2, 3.6 Hz, 1H), 3.45 (dd, *J* = 14.0, 3.7 Hz, 1H), 2.89 – 2.72 (m, 2H), 1.81 – 1.69 (m, 1H), 1.25 – 1.09 (m, 1H), 0.95 – 0.82 (m, 1H), 0.59 (dd, *J* = 8.3, 6.6 Hz, 6H). ^{13}C NMR (126 MHz, DMSO-d_6) δ: 170.6, 169.0 (d, *J* = 4.9 Hz), 149.6, 146.9, 139.0, 137.5, 134.1, 130.8, 130.5, 125.2, 123.4, 121.3, 120.1, 53.8, 45.8, 44.8, 34.1 (d, *J* = 4.3 Hz), 33.7, 26.3 (d, *J* = 15.6 Hz), 22.9, 21.1. ^{31}P NMR (202 MHz, DMSO) δ: 21.5. HRMS (ESI+) calculated for $C_{20}H_{25}Cl_2N_3O_5P$ $[M+H]^+$ 488.0903, found 488.0898.

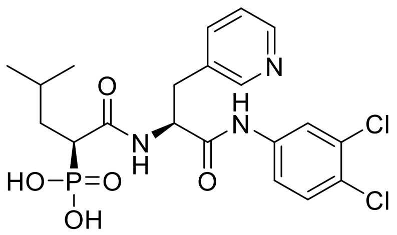

(*S*)-25: ^{1}H NMR (500 MHz, DMSO-d_6) δ: 10.36 (s, 1H), 8.46 (s, 1H), 8.42 (d, *J* = 4.7 Hz, 1H), 8.35 (d, *J* = 8.0 Hz, 1H), 8.07 (s, 1H), 7.76 – 7.67 (m, 2H), 7.56 (d, *J* = 9.0 Hz, 1H), 7.31 (dd, *J* = 8.0, 4.9 Hz, 1H), 4.65 – 4.54 (m, 1H), 3.32 – 3.06 (m, 2H), 2.76 (ddd, *J* = 22.5, 12.0, 3.3 Hz, 1H), 1.94 – 1.77 (m, 1H), 1.30 (ddd, *J* = 30.0, 15.4, 8.5 Hz, 1H), 1.15 (d, *J* = 16.3 Hz, 1H), 0.72 (dd, *J* = 37.6, 6.5 Hz, 6H). ^{13}C NMR (126 MHz, DMSO-d_6) δ: 170.2, 169.4 (d, *J* = 2.7 Hz), 149.8, 147.0, 137.2, 133.8, 130.8, 130.5, 124.9, 123.4, 120.9, 119.8, 54.9, 46.4, 45.5, 35.3 (q, *J* = 16.8, 4.1 Hz), 33.4, 26.0 (d, *J* = 14.8 Hz), 23.3, 21.0. ^{31}P NMR (202 MHz, DMSO-d_6) δ: 20.7. HRMS (ESI+) calculated for $C_{20}H_{25}Cl_2N_3O_5P$ $[M+H]^+$ 488.0903, found 488.0901.

Tert-butyl (2-(diethoxyphosphoryl)-4-methylpentanoyl)-L-valinate (26a)

A solution of 2-(diethoxyphosphoryl)-4-methylpentanoic acid **6** (1.00 g, 3.96 mmol) in DMF (20 mL) was cooled down to 0 °C, before TBTU (1.40 g, 4.36 mmol), NMM (1,09 mL, 9.90 mmol) and HCl·Val-OtBu (0.91 g, 4.36 mmol) were added. The reaction mixture was warmed up to room temperature, and after complete conversion (TLC) diluted with ethyl acetate and washed successively with water (twice), $KHSO_4$ (1N) solution, saturated aqueous $NaHCO_3$ solution and brine. After drying over Na_2SO_4 and removing the solvent under reduced pressure, the desired compound **26a** (1.61 g, 3.96 mmol, quant.) was obtained as a colorless resin. The crude product was used without further purification for the next step.

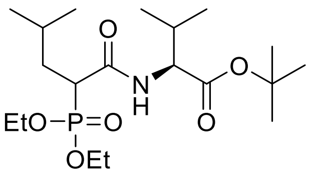

Mixture of diastereomeres. Major diastereomer: ^{1}H NMR (500 MHz, $CDCl_3$) δ: 6.62 – 6.75 (m, 1H), 4.39 – 4.48 (m, 1H), 4.11 – 4.18 (m, 4H), 2.81 – 2.89 (m, 1H), 2.80 – 2.85 (m, 1H), 2.09 – 2.23 (m, 1H), 1.91 – 2.05 (m, 1H), 1.62 – 1.77 (m, 1H), 1.51 – 1.62 (m, 1H), 1.45 – 1.50 (m, 9H), 1.32 (td, J = 7.1, 1.7 Hz, 5H), 0.86 – 1.00 (m, 12H). ^{13}C NMR (126 MHz, $CDCl_3$) δ: 170.6, 167.3 (d, J=2.76 Hz), 81.7, 62.7 (d, J = 7.35 Hz), 62.5 (d, J = 7.35 Hz), 57.8, 45.0, 44.0, 35.7 (d, J = 5.52 Hz), 31.4, 28.0, 26.6, 26.5, 23.1, 21.2, 18.9, 17.6, 16.3 (br d, J = 2.76 Hz), 16.3 (d, J = 1.84 Hz). *Minor diastereomer (selected signals):* ^{13}C NMR (126 MHz, $CDCl_3$) δ: 170.6, 167.4 (d, J = 2.76 Hz), 57.7, 45.3, 44.2, 35.9 (d, J = 4.60 Hz), 28.0, 26.5, 26.4, 21.1, 18.9, 17.5. HRMS (ESI+) calculated for $C_{19}H_{39}NO_6P$ $[M+H]^+$ 408.2510, found 408.2508.

(2-(diethoxyphosphoryl)-4-methylpentanoyl)-L-valine (26)

Tert-butyl (2-(diethoxyphosphoryl)-4-methylpentanoyl)-L-valinate **26a** (1.30 g, 3.19 mmol) was dissolved in DCM (3.2 mL) and TFA (1.80 g, 15.90 mmol, 1.23 mL) was added. The reaction was allowed to stir at room temperature overnight, progress was monitored by LC-MS. Another 1.23 mL of TFA were added and the reaction mixture was allowed to stir at room temperature for another night. Upon completion, the solvents were removed under reduced pressure, the desired compound **26** was obtained as a brown oil (1.12 g, 3.19 mmol, quant.). The crude product was used without further purification for the next step.

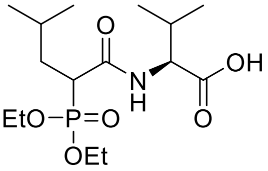

Mixture of diastereomeres. Major diastereomer: ^{1}H NMR (500 MHz, $CDCl_3$): δ = 7.44 – 7.77 (m, 1H), 7.08 – 7.14 (m, 1H), 4.53 – 4.64 (m, 1H), 4.08 – 4.24 (m, 4H), 3.03 – 3.14 (m, 1H), 2.20 – 2.32 (m, 1H), 1.96 – 2.08 (m, 1H), 1.57 – 1.69 (m, 1H), 1.45 – 1.56 (m, 1H), 1.30 – 1.37 (m, 6H), 0.85 – 1.02 (m, 12H). ^{13}C NMR (126 MHz, $CDCl_3$): δ = 174.5, 167.7, 159.1, 63.5, 57.5, 44.8, 43.7, 35.4, 31.0, 26.5, 23.0, 21.0, 18.9, 17.4, 16.2. ^{31}P NMR (202 MHz, $CDCl_3$): δ: 26.0. *Minor diastereomer (selected signals):* ^{13}C NMR (126 MHz, $CDCl_3$): δ = 174.4, 167.9, 57.5, 44.9, 43.9, 30.9, 23.1, 21.0, 19.0. ^{31}P NMR (202 MHz, $CDCl_3$): δ = 25.7. HRMS (ESI+) calculated for $C_{15}H_{31}NO_6P$ $[M+H]^+$ 352.1884, found 352.1871.

(1-(((*S*)-1-((4-chlorophenyl)amino)-3-methyl-1-oxobutan-2-yl)amino)-4-methyl-1-oxopentan-2-yl)phosphonic acid (27)

According to **GP3**, (2-(diethoxyphosphoryl)-4-methylpentanoyl)-L-valine **26** (96 mg, 0.27 mmol) was reacted with 4-chloroaniline (38mg, 0.30 mmol), NMM (76 µL, 0.68 mmol) and TBTU (96 mg, 0.30 mmol) in DCM (2.7 mL) to afford the diethyl phosphonate dipeptide (74 mg, 0.16 mmol, 53% yield) which was used without further purification in the next step. Diethyl phosphonate dipeptide (74 mg, 0.16 mmol) was deprotected by bromotrimethylsilane (0.17 mL, 1.12 mmol) in DCM (1.6 mL). Then MeOH was added and stirred at room temperature for 30 minutes to cleave the previously formed TMS ester. The solvents were removed under reduced pressure and the crude product was purified *via* a Dionex Ultimate 3000 (Thermo Scientific) with a Nucleodur C18 Graphity column (250mm x 16 mm, particle size 5µm) using UV-detection to afford dipeptide **27** (20.0 mg, 0.05 mmol, 31%) as a colorless solid.

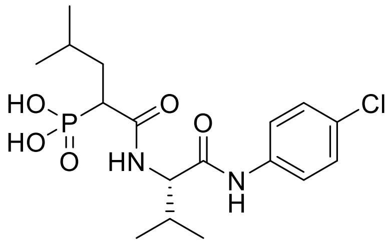

Mixture of diastereomeres. Major diastereomer: ^{1}H NMR (500 MHz, MeOH-d_4) δ: 7.68 – 7.63 (m, 2H), 7.30 – 7.27 (m, 2H), 4.45 (d, *J* = 5.2 Hz, 1H), 3.24 (ddd, *J* = 23.5, 11.5, 2.8 Hz, 1H), 2.40 (dd, *J* = 6.9, 5.2 Hz, 1H), 2.09 (dddd, *J* = 13.5, 11.5, 7.2, 4.5 Hz, 1H), 1.63 – 1.47 (m, 2H), 1.00 (dd, *J* = 11.3, 6.9 Hz, 6H), 0.95 (d, *J* = 6.4 Hz, 6H). ^{13}C NMR (126 MHz, MeOH-d_4) δ: 172.4, 138.2, 129.6, 123.4, 60.6, 46.9, 45.9, 36.2 (d, *J* = 4.7 Hz), 31.1, 28.2 (d, *J* = 15.1 Hz), 23.6, 21.7, 19.8, 18.9, 17.7. ^{31}P NMR (202 MHz, MeOH-d_4) δ: 22.6. *Minor diastereomer (selected signals):* ^{1}H NMR (500 MHz, MeOH-d_4) δ: 7.58 – 7.54 (m, 2H), 3.05 (ddd, *J* = 22.6, 11.6, 2.7 Hz, 1H), 2.15 (dd, *J* = 14.5, 7.5 Hz, 1H), 2.04 – 1.96 (m, 1H), 1.08 – 1.02 (m, 6H), 0.90 (dd, *J* = 11.4, 6.1 Hz, 6H). ^{13}C NMR (126 MHz, MeOH-d_4) δ: 130.4, 122.7, 61.3, 46.7, 45.7, 37.1, 32.2, 28.0 (d, *J* = 15.1 Hz). ^{31}P NMR (202 MHz, MeOH-d_4) δ: 22.5. HRMS (ESI-) calculated for $C_{17}H_{25}ClN_2O_5P$ $[M-H]^-$ 403.1195, found 403.1197.

(1-(((*S*)-1-((3-chlorophenyl)amino)-3-methyl-1-oxobutan-2-yl)amino)-4-methyl-1-oxopentan-2-yl)phosphonic acid (28)

According to **GP3**, (2-(diethoxyphosphoryl)-4-methylpentanoyl)-L-valine **26** (129.0 mg, 0.37 mmol) was reacted with 3-chloroaniline (42 µL, 0.40 mmol), NMM (102 µL, 0.92 mmol) and

TBTU (128 mg, 0.40 mmol) in DCM (3.7 mL) to afford the diethyl phosphonate dipeptide (105 mg, 0.23 mmol, 62% yield) which was used without further purification in the next step. Diethyl phosphonate dipeptide (93 mg, 0.20 mmol) was deprotected by bromotrimethylsilane (0.18 mL, 1.40 mmol) in DCM (2.0 mL). Then MeOH was added and stirred at room temperature for 30 minutes to cleave the previously formed TMS ester. The solvents were removed under reduced pressure and the crude product was purified *via* a Dionex Ultimate 3000 (Thermo Scientific) with a Nucleodur C18 Graphity column (250mm x 16 mm, particle size 5µm) using UV-detection to afford dipeptide **28** (35.0 mg, 0.086 mmol, 43%) as a colorless solid.

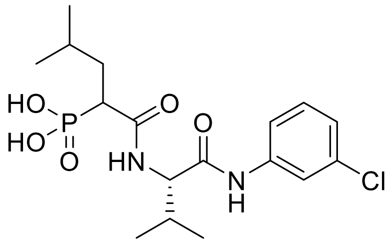

Mixture of diastereomeres. Major diastereomer: ^{1}H NMR (500 MHz, MeOH-d_4) δ: 7.76 (dd, J = 8.1, 1.6 hz, 1H), 7.60 (dd, J = 8.0, 1.6 Hz, 1H), 7.45 (dt, J = 8.0, 1.8 Hz, 2H), 4.50 (d, J = 5.8 Hz, 1H), 3.24 – 3.15 (m, 1H), 2.36 (dq, J = 13.5, 6.8 Hz, 1H), 2.13 – 2.00 (m, 1H), 1.58 (dddd, J = 26.0, 13.2, 6.9, 2.6 Hz, 2H), 1.08 (dd, J = 6.8, 2.9 Hz, 6H), 0.95 (dd, J = 6.5, 1.3 Hz, 6H). ^{13}C NMR (126 MHz, MeOH-d_4) δ: 172.8, 172.2, 135.5, 130.7, 130.2, 128.5, 128.3, 60.6, 46.8, 45.8, 36.6, 31.4, 28.2, 28.1, 23.7 (d, J = 6.3 Hz), 21.8, 18.1. ^{31}P NMR (202 MHz, MeOH-d_4) δ: 22.3. *Minor diastereomer (selected signals):* ^{1}H NMR (500 MHz, MeOH-d_4) δ: 7.30 (tdd, J = 7.8, 4.5, 1.5 Hz, 2H), 7.20 (dtd, J = 11.9, 7.7, 1.6 Hz, 2H), 4.40 (d, J = 7.4 Hz, 1H), 3.07 (ddd, J = 22.5, 11.4, 2.8 Hz, 1H), 2.29 – 2.17 (m, 1H), 1.06 (dd, J = 6.9, 1.5 Hz, 6H), 0.91 (t, J = 6.4 Hz, 6H). ^{13}C NMR (126 MHz, MeOH-d_4) δ: 172.5, 130.7, 128.4, 127.8, 126.9, 61.1, 46.7, 45.6, 37.1, 31.9, 28.0, 27.9, 21.7, 19.9 (d, J = 3.2 Hz), 18.8. ^{31}P NMR (202 MHz, MeOH-d_4) δ: 22.6. HRMS (ESI+) calculated for $C_{17}H_{27}ClN_2O_5P$ $[M+H]^+$ 405.1341, found 405.1335.

(4-methyl-1-(((*S*)-3-methyl-1-oxo-1-((4-(trifluoromethyl)phenyl)amino)butan-2-yl)amino)-1-oxopentan-2-yl)phosphonic acid (29)

According to **GP3**, (2-(diethoxyphosphoryl)-4-methylpentanoyl)-L-valine **26** (97.0 mg, 0.28 mmol) was reacted with 4-(trifluoromethyl)-aniline (39µL mg, 0.31 mmol), NMM (78 µL, 0.70 mmol) and TBTU (100 mg, 0.31 mmol) in DCM (2.8 mL) to afford the diethyl phosphonate dipeptide (138 mg, 0.28 mmol, quant.) which was used without further purification in the next step. Diethyl phosphonate dipeptide (138 mg, 0.28 mmol) was deprotected by bromotrimethylsilane (0.26 mL, 1.96 mmol) in DCM (2.8 mL). Then MeOH was added and

stirred at room temperature for 30 minutes to cleave the previously formed TMS ester. The solvents were removed under reduced pressure and the crude product was purified *via* a Dionex Ultimate 3000 (Thermo Scientific) with a Nucleodur C18 Graphity column (250mm x 16 mm, particle size 5µm) using UV-detection to afford dipeptide **29** (33.0 mg, 0.075 mmol, 27%) as a colorless solid.

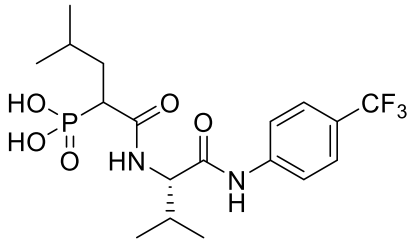

Mixture of diastereomeres. Major diastereomer: ^{1}H NMR (500 MHz, MeOH-d_4) δ: 7.83 (d, *J* = 8.5 Hz, 2H), 7.52 (d, *J* = 8.7 Hz, 2H), 4.42 (d, *J* = 5.2 Hz, 1H), 3.18 (ddd, *J* = 23.5, 11.6, 2.7 Hz, 1H), 2.35 (ddt, *J* = 13.8, 11.8, 6.8 Hz, 1H), 2.08 – 1.98 (m, 1H), 1.59 – 1.40 (m, 2H), 0.94 (dd, *J* = 11.7, 7.0 Hz, 6H), 0.89 (d, *J* = 6.4 Hz, 6H). ^{13}C NMR (126 MHz, MeOH-d_4) δ: 199.6, 172.7, 144.3, 134.0, 130.5, 121.1, 60.7, 47.0, 46.0, 36.3 (d, *J* = 4.7 Hz), 31.2, 28.2 (d, *J* = 15.1 Hz), 26.5, 23.6, 21.7, 19.8, 17.7. ^{31}P NMR (202 MHz, MeOH-d_4) δ: 22.4. ^{19}F NMR (470 MHz, MeOH-d_4) δ: -63.6. *Minor diastereomer (selected signals):* ^{1}H NMR (500 MHz, MeOH-d_4) δ: 7.72 (d, *J* = 8.5 Hz, 2H), 4.25 (d, *J* = 7.5 Hz, 1H), 3.06 – 2.95 (m, 1H), 2.11 (dt, *J* = 13.8, 6.8 Hz, 1H), 1.98 – 1.90 (m, 1H). ^{13}C NMR (126 MHz, MeOH-d_4) δ: 172.5, 130.7, 120.4, 61.3, 37.2, 32.1, 27.9, 21.8, 18.8. ^{31}P NMR (202 MHz, MeOH-d_4) δ: 22.3. ^{19}F NMR (470 MHz, MeOH-d_4) δ: -63.6. HRMS (ESI-) calculated for $C_{18}H_{25}F_3N_2O_5P$ [M-H]$^-$ 437.1459, found 437.1461.

(1-(((*S*)-1-((4-acetylphenyl)amino)-3-methyl-1-oxobutan-2-yl)amino)-4-methyl-1-oxopentan-2-yl)phosphonic acid (30)

According to **GP3**, (2-(diethoxyphosphoryl)-4-methylpentanoyl)-L-valine **26** (114.0 mg, 0.32 mmol) was reacted with 4-aminoacetophenone (47 mg, 0.35 mmol), NMM (89 µL, 0.80 mmol) and TBTU (112 mg, 0.35 mmol) in DCM (3.2 mL) to afford the diethyl phosphonate dipeptide (139 mg, 0.30 mmol, 94% yield) which was used without further purification in the next step. Diethyl phosphonate dipeptide (139 mg, 0.30 mmol) was deprotected by bromotrimethylsilane (0.28 mL, 2.1 mmol) in DCM (3.0 mL). Then MeOH was added and stirred at room temperature for 30 minutes to cleave the previously formed TMS ester. The solvents were removed under reduced pressure and the crude product was purified *via* a Dionex Ultimate 3000 (Thermo Scientific) with a Nucleodur C18 Graphity column (250mm x 16 mm, particle size 5µm) using UV-detection to afford dipeptide **30** (34 mg, 0.082 mmol, 27%) as a pale pink solid.

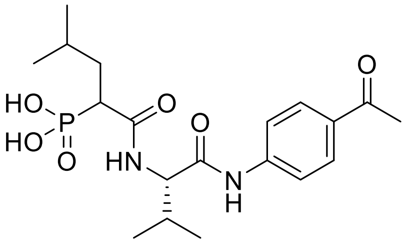

Mixture of diastereomeres. Major diastereomer: ^{1}H NMR (500 MHz, MeOH-d_4) δ: 7.98 – 7.93 (m, 2H), 7.87 – 7.82 (m, 2H), 4.48 (d, *J* = 5.3 Hz, 1H), 3.22 (ddd, *J* = 23.3, 11.5, 2.7 Hz, 1H), 2.57 (s, 3H), 2.46 – 2.35 (m, 1H), 2.09 (dddd, *J* = 13.4, 11.6, 6.9, 4.1 Hz, 1H), 1.64 – 1.47 (m, 2H), 1.01 (dd, *J* = 11.5, 6.9 Hz, 6H), 0.95 (d, *J* = 6.4, 6H). ^{13}C NMR (126 MHz, MeOH-d_4) δ: 198.1, 171.3, 142.9, 132.6, 129.1, 119.7, 59.3, 45.6, 44.6, 34.9 (d, *J* = 4.7 Hz), 29.8, 26.8 (d, *J* = 15.1 Hz), 25.1, 22.2, 20.3, 18.4, 16.3. ^{31}P NMR (202 MHz, MeOH-d_4) δ: 22.2. *Minor diastereomer (selected signals):* ^{1}H NMR (500 MHz, MeOH-d_4) δ: 7.76 – 7.71 (m, 2H), 4.33 (d, *J* = 7.4 Hz, 1H), 3.05 (ddd, *J* = 22.4, 11.6, 2.8 Hz, 1H), 2.21 – 2.14 (m, 1H). ^{13}C NMR (126 MHz, MeOH-d_4) δ: 129.3, 119.0, 60.0, 30.7, 26.6 (d, *J* = 15.1 Hz), 22.2, 20.4, 17.4. ^{31}P NMR (202 MHz, MeOH-d_4) δ: 22.2. HRMS (ESI+) calculated for $C_{19}H_{30}N_2O_6P$ $[M+H]^+$ 413.1836, found 413.1829.

(4-Methyl-1-(((*S*)-3-methyl-1-oxo-1-((4-(2,2,2-trifluoroacetyl)phenyl)amino)butan-2-yl)amino)-1-oxopentan-2-yl)phosphonic acid (31)

According to **GP3**, (2-(diethoxyphosphoryl)-4-methylpentanoyl)-L-valine **26** (70.0 mg, 0.200 mmol) was reacted with 1-(4-aminophenyl)-2,2,2-trifluoroethan-1-one (38 mg, 0.200 mmol), NMM (55 µL, 0.500 mmol) and IBCF (27 µL, 0.200 mmol) in DCM (3.1 mL) to afford the diethyl phosphonate dipeptide (101 mg, 0.193 mmol) which was used without further purification in the deprotection with bromotrimethylsilane (0.26 mL, 1.93 mmol) in DCM (2 mL). Upon completion of the reaction, MeOH was added and the solvents were removed under reduced pressure and the crude product was purified *via* a Dionex Ultimate 3000 (Thermo Scientific) with a Nucleodur C18 Graphity column (250mm x 16 mm, particle size 5µm) using UV-detection to afford dipeptide **31** (5.2 mg, 0.011 mmol, 6%) as a colorless solid.

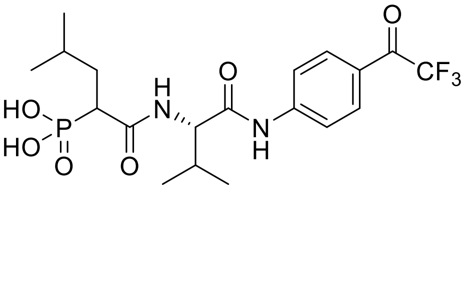

Mixture of diastereomeres. Major diastereomer: ^{1}H NMR (500 MHz, MeOH-d_4) δ: 7.71 – 7.69 (m, 2 H), 7.54 – 7.52 (m, 2 H), 4.47 (d, J = 5.34 Hz, 1 H), 2.44 – 2.37 (m, 1 H), 2.18 – 2.10 (m, 1 H), 2.09 – 1.99 (m, 1 H), 1.62 – 1.49 (m, 3 H), 1.03 (d, *J* = 7.02 Hz, 3 H), 1.01 (d, J = 7.02 Hz, 3 H), 0.95 (d, *J* = 6.56 Hz, 6 H). 13C NMR (126 MHz, MeOH-d_4): 172.6, 172.4, 172.0, 140.6, 131.9, 129.9, 121.6, 120.8, 72.3, 61.5, 60.8, 50.0, 47.0, 46.0, 40.6, 36.4, 32.4, 31.4, 28.3, 23.8, 21.8, 19.9, 17.9. 31P NMR (203 MHz, MeOH-d_4) δ: 22.7. ^{19}F NMR (470 MHz, MeOH-d_4) δ: –84.7. *Minor diastereomer (selected signals):* ^{1}H NMR (500 MHz, MeOH-d_4) δ: 7.62 – 7.60 (m, 2 H), 4.32 (d, *J* = 7.63 Hz, 1 H), 0.92 (d, *J* = 6.10 Hz, 3 H), 0.90 (d, *J* = 6.10 Hz, 3 H). ^{13}C NMR (126 MHz, MeOH-d_4): 172.5, 140.7, 130.0, 46.9, 45.9, 19.1. HRMS (ESI+) calculated for $C_{19}H_{27}F_3N_2O_6P$ $[M+H]^+$ 467.1553, found 467.1552.

(4-Methyl-1-(((*S*)-3-methyl-1-oxo-1-(p-tolylamino)butan-2-yl)amino)-1-oxopentan-2-yl)phosphonic acid (32)

According to **GP3**, (2-(diethoxyphosphoryl)-4-methylpentanoyl)-L-valine **26** (101.0 mg, 0.29 mmol) was reacted with p-methylaniline (34 mg, 0.32 mmol), NMM (81 µL, 0.73 mmol) and TBTU (103 mg, 0.32 mmol) in DCM (2.9 mL) to afford the diethyl phosphonate dipeptide (116 mg, 0.26 mmol, 90% yield), which was used without further purification in the next step. Diethyl phosphonate dipeptide (116 mg, 0.26 mmol) was deprotected by bromotrimethylsilane (0.24 mL, 1.82 mmol) in DCM (2.6 mL). Then MeOH was added and stirred at room temperature for 30 minutes to cleave the previously formed TMS ester. The solvents were removed under reduced pressure and the crude product was purified *via* a Dionex Ultimate 3000 (Thermo Scientific) with a Nucleodur C18 Graphity column (250mm x 16 mm, particle size 5µm) using UV-detection to afford dipeptide **32** (29.0 mg, 0.075 mmol, 29%) as a pale pink solid.

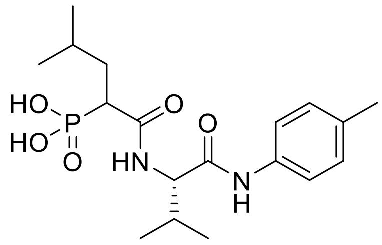

Mixture of diastereomeres. Major diastereomer: ^{1}H NMR (500 MHz, MeOH-d_4) δ: 7.52 – 7.45 (m, 2H), 7.11 (dd, *J* = 8.3, 6.5 Hz, 2H), 4.44 (d, *J* = 5.4 Hz, 1H), 3.27 – 3.15 (m, 1H), 2.39 (dd, *J* = 6.7, 5.2 Hz, 1H), 2.29 (s, 3H), 2.13 – 2.04 (m, 1H), 1.64 – 1.47 (m, 2H), 1.00 (dd, *J* = 9.8, 6.8 Hz, 6H), 0.95 (d, *J* = 6.4 Hz, 6H). ^{13}C NMR (126 MHz, MeOH-d_4) δ: 172.2, 136.6, 135.3, 130.1, 122.2, 60.6, 46.9, 45.9, 36.3 (d, *J* = 4.5 Hz), 31.2, 28.2 (d, *J* = 15.1 Hz), 23.6, 21.7, 20.9, 19.8, 18.9, 17.7. ^{31}P NMR (202 MHz, MeOH-d_4) δ: 22.3. *Minor diastereomer (selected signals):*

^{1}H NMR (500 MHz, MeOH-d_4) δ: 7.44 – 7.37 (m, 2H), 4.29 (d, *J* = 7.6 Hz, 1H), 3.03 (dd, *J* = 22.2, 11.4 Hz, 1H), 2.21 – 2.13 (m, 1H). ^{13}C NMR (126 MHz, MeOH-d_4) δ: 172.0, 136.7, 135.2, 130.3, 121.6, 61.2, 45.9, 37.3, 32.2, 28.0 (d, *J* = 14.8 Hz), 21.8. ^{31}P NMR (202 MHz, MeOH-d_4) δ: 22.2 ppm. HRMS (ESI+) calculated for $C_{18}H_{30}N_2O_5P$ $[M+H]^+$ 385,1887, found 358.1880.

(1-(((*S*)-1-((4-Methoxyphenyl)amino)-3-methyl-1-oxobutan-2-yl)amino)-4-methyl-1-oxopentan-2-yl)phosphonic acid (33)

According to **GP3**, (2-(diethoxyphosphoryl)-4-methylpentanoyl)-L-valine **26** (100.0 mg, 0.28 mmol) was reacted with 4-methoxyaniline (38 mg, 0.31 mmol), NMM (78 µL, 0.70 mmol) and TBTU (100 mg, 0.31 mmol) in DCM (2.8 mL) to afford the diethyl phosphonate dipeptide (128 mg, 0.28 mmol, quant.), which was used without further purification in the next step. Diethyl phosphonate dipeptide (128 mg, 0.28 mmol) was deprotected by bromotrimethylsilane (0.26 mL, 1.96 mmol) in DCM (2.8 mL). Then MeOH was added and stirred at room temperature for 30 minutes to cleave the previously formed TMS ester. The solvents were removed under reduced pressure and the crude product was purified *via* a Dionex Ultimate 3000 (Thermo Scientific) with a Nucleodur C18 Graphity column (250mm x 16 mm, particle size 5µm) using UV-detection to afford dipeptide **33** (55.0 mg, 0.14 mmol, 50%) as a colorless solid.

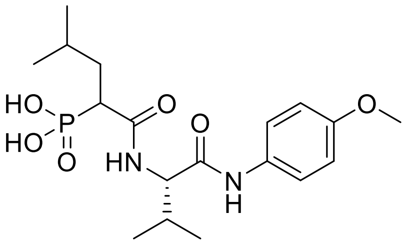

Mixture of diastereomeres. Major diastereomer: ^{1}H NMR (500 MHz, MeOH-d_4) δ: 7.55 – 7.49 (m, 2H), 6.92 – 6.85 (m, 2H), 4.45 (d, *J* = 5.3 hz, 1H), 3.79 (s, 3H), 3.23 (dtd, *J* = 22.4, 11.2, 2.3 Hz, 1H), 2.41 (dd, *J* = 6.9, 5.4 Hz, 1H), 2.14 – 2.07 (m, 1H), 1.66 – 1.51 (m, 2H), 1.03 (dd, *J* = 9.6, 6.9 Hz, 6H), 0.97 (d, *J* = 6.5 Hz, 6H). ^{13}C NMR (126 MHz, MeOH-d_4) δ: 170.7, 156.8, 130.8, 122.5, 113.4, 59.1, 54.4, 45.5, 44.5, 34.9 (d, *J* = 4.6 Hz), 29.8, 26.8 (d, *J* = 15.1 Hz), 22.2, 20.3, 18.4, 16.3. ^{31}P NMR (202 MHz, MeOH-d_4) δ: 22.5. *Minor diastereomer (selected signals):* ^{1}H NMR (500 MHz, MeOH-d_4) δ: 7.46 – 7.39 (m, 2H), 4.27 (d, *J* = 7.7 Hz, 1H), 3.03 (ddd, *J* = 22.4, 11.6, 2.8 Hz, 1H), 2.16 (d, *J* = 7.0 Hz, 1H). ^{13}C NMR (126 MHz, MeOH-d_4) δ: 132.3, 123.3, 115.0, 61.2, 45.8, 37.2, 32.2, 28.0 (d, *J* = 15.1 Hz), 23.6, 21.8, 19.0. ^{31}P NMR (202 MHz, MeOH-d_4) δ: 22.4. HRMS (ESI+) calculated for $C_{18}H_{30}N_2O_6P$ $[M+H]^+$ 401.1836, found 401.1830.

(1-(((*S*)-1-((4-isopropoxyphenyl)amino)-3-methyl-1-oxobutan-2-yl)amino)-4-methyl-1-oxopentan-2-yl)phosphonic acid (34)

According to **GP3**, (2-(diethoxyphosphoryl)-4-methylpentanoyl)-L-valine **26** (106.0 mg, 0.30 mmol) was reacted with 4-isopropoxyaniline (50 mg, 0.33 mmol), NMM (83 µL, 0.75 mmol) and TBTU (106 mg, 0.33 mmol) in DCM (3.0 mL) to afford the diethyl phosphonate dipeptide (143 mg, 0.29 mmol, 97% yield) which was used without further purification in the next step. Diethyl phosphonate dipeptide (132 mg, 0.27 mmol) was deprotected by bromotrimethylsilane (0.25 mL, 1.89 mmol) in DCM (2.7 mL). Then MeOH was added and stirred at room temperature for 30 minutes to cleave the previously formed TMS ester. The solvents were removed under reduced pressure and the crude product was purified *via* a Dionex Ultimate 3000 (Thermo Scientific) with a Nucleodur C18 Graphity column (250mm x 16 mm, particle size 5µm) using UV-detection to afford dipeptide **34** (57.0 mg, 0.13 mmol, 48%) as a colorless solid.

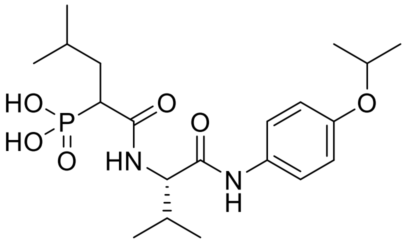

Mixture of diastereomeres. Major diastereomer: ^{1}H NMR (500 MHz, MeOH-d_4) δ: 7.50 – 7.46 (m, 2H), 6.85 (m, 2H), 4.55 (qd, *J* = 6.1, 3.0 Hz, 1H), 4.43 (d, *J* = 5.3 Hz, 1H), 3.22 (ddd, *J* = 23.4, 11.5, 2.8 Hz, 1H), 2.39 (ddt, *J* = 13.7, 11.5, 6.8 Hz, 1H), 2.08 (dtd, *J* = 13.4, 7.2, 3.5 Hz, 1H), 1.66 – 1.46 (m, 2H), 1.29 (dd, *J* = 6.0, 2.6 Hz, 6H), 1.01 (dd, *J* = 9.6, 6.9 Hz, 6H), 0.95 (d, *J* = 6.4 Hz, 6H). ^{13}C NMR (126 MHz, MeOH-d_4) δ: 172.1, 156.3, 132.2, 123.9, 117.1, 71.3, 60.5, 46.9, 45.9, 40.4, 37.3, 36.3 (d, *J* = 4.6 Hz), 32.2, 31.2, 28.2 (d, *J* = 15.2 Hz), 23.6, 22.3, 21.7, 19.8, 17.7. ^{31}P NMR (202 MHz, MeOH-d_4) δ: 22.4. *Minor diastereomer (selected signals):* ^{1}H NMR (500 MHz, MeOH-d_4) δ: 7.41 (d, *J* = 9.0 Hz, 2H), 4.27 (d, *J* = 7.6 Hz, 1H), 3.03 (ddd, *J* = 22.4, 11.5, 2.8 Hz, 1H), 2.16 (dt, *J* = 13.9, 6.9 Hz, 1H), 2.05 – 1.96 (m, 1H), 1.54 – 1.46 (m, 2H), 0.91 (dd, *J* = 8.7, 6.1 Hz, 6H). ^{13}C NMR (126 MHz, MeOH-d_4) δ: 123.33 117.22 71.3, 61.2, 28.0 (d, *J* = 14.9 Hz), 21.8, 18.9. ^{31}P NMR (202 MHz, MeOH-d_4) δ: 22.3. HRMS (ESI+) calculated for $C_{20}H_{34}N_2O_6P$ [M+H]$^+$ 429.2149, found 429.2141.

(4-methyl-1-(((*S*)-3-methyl-1-oxo-1-((4-phenoxyphenyl)amino)butan-2-yl)amino)-1-oxopentan-2-yl)phosphonic acid (35)

According to **GP3**, (2-(diethoxyphosphoryl)-4-methylpentanoyl)-L-valine **26** (110.0 mg, 0.31 mmol) was reacted with 4-phenoxyaniline (63.0 mg, 0.34 mmol), NMM (86 µL, 0.77 mmol) and TBTU (109.0 mg, 0.33 mmol) in DCM (3.1 mL) to afford the diethyl phosphonate dipeptide (158.0 mg, 0.30 mmol, 97% yield) which was used without further purification in the next step. Diethyl phosphonate dipeptide (158 mg, 0.30 mmol) was deprotected by bromotrimethylsilane (0.28 mL, 2.1 mmol) in DCM (3.0 mL). Then MeOH was added and stirred at room temperature for 30 minutes to cleave the previously formed TMS ester. The solvents were removed under reduced pressure and the crude product was purified *via* a Dionex Ultimate 3000 (Thermo Scientific) with a Nucleodur C18 Graphity column (250mm x 16 mm, particle size 5µm) using UV-detection to afford dipeptide **35** (48.0 mg, 0.10 mmol, 33%) as a colorless solid.

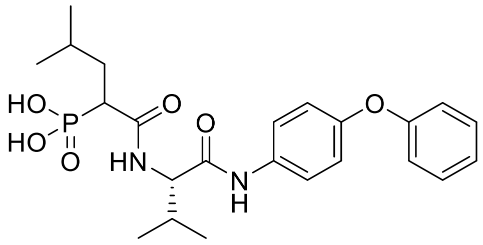

Mixture of diastereomeres. Major diastereomer: ^{1}H NMR (500 MHz, MeOH-d_4) δ: 7.65 – 7.59 (m, 2H), 7.33 (ddd, *J* = 8.5, 7.4, 2.4 Hz, 3H), 6.98 – 6.89 (m, 6H), 4.45 (d, *J* = 5.2 Hz, 1H), 3.22 (ddd, *J* = 23.4, 11.5, 2.7 Hz, 1H), 2.46 – 2.37 (m, 1H), 2.14 – 2.05 (m, 1H), 1.64 – 1.55 (m, 2H), 1.01 (dd, *J*=9.5, 7.0, 6H), 0.95 (d, *J* = 6.4 Hz, 6H). ^{13}C NMR (126 MHz, MeOH-d_4) δ: 172.3, 159.1, 155.1, 134.9, 130.9, 130.8, 124.2, 123.9, 123.2, 120.1, 119.4, 60.5, 47.0, 46.0, 40.5, 36.3 (d, *J* = 4.8 Hz), 32.1, 31.1, 28.2 (d, *J* = 15.1 Hz), 23.6, 21.7, 19.8, 17.7. ^{31}P NMR (202 MHz, MeOH-d_4) δ: 22.3. *Minor diastereomer (selected signals):* ^{1}H NMR (500 MHz, MeOH-d_4) δ: 7.57 – 7.51 (m, 2H), 7.10 – 7.05 (m, 5H), 4.29 (d, *J* = 7.4 Hz, 1H), 2.18 (d, *J* = 6.9 Hz, 1H), 2.01 (dd, *J* = 10.0, 9.4 Hz, 1H). ^{13}C NMR (126 MHz, MeOH-d_4) δ: 172.5, 159.1, 155.1, 134.9, 131.1, 124.9, 124.2, 121.0, 120.0, 119.4, 61.2, 37.3, 28.0 (d, *J* = 15.0 Hz), 18.9. ^{31}P NMR (202 MHz, MeOH-d_4) δ: 22.1. HRMS (ESI+) calculated for $C_{23}H_{32}N_2O_6P$ [M+H]$^+$ 463.1992, found 463.1985.

((*R*)-1-(((*S*)-1-((4-(1H-Imidazol-1-yl)phenyl)amino)-3-methyl-1-oxobutan-2-yl)amino)-4-methyl-1-oxopentan-2-yl)phosphonic acid ((*R*)-36)

((*S*)-1-(((*S*)-1-((4-(1H-Imidazol-1-yl)phenyl)amino)-3-methyl-1-oxobutan-2-yl)amino)-4-methyl-1-oxopentan-2-yl)phosphonic acid ((*S*)-36)

According to **GP3**, (2-(diethoxyphosphoryl)-4-methylpentanoyl)-L-valine **26** (113.0 mg, 0.32 mmol) was reacted with 4-(1H-imidazol-1-yl)aniline (56mg, 0.35 mmol), NMM (89 µL, 0.80 mmol) and TBTU (112.0 mg, 0.35 mmol) in DCM (3.2 mL) to afford the diethyl phosphonate dipeptide (158 mg, 0.32 mmol, quant.) which was used without further purification in the next step. Diethyl phosphonate dipeptide (158.0 mg, 0.32 mmol) was deprotected by bromotrimethylsilane (0.30 mL, 2.24 mmol) in DCM (3.2 mL). Then MeOH was added and stirred at room temperature for 30 minutes to cleave the previously formed TMS ester. The solvents were removed under reduced pressure and the crude product was purified *via* a Dionex Ultimate 3000 (Thermo Scientific) with a Nucleodur C18 Graphity column (250mm x 16 mm, particle size 5µm) using UV-detection to afford the dipeptide diastereomeres **(*R*)-36** (11.0 mg, 0.025 mmol, 8%) and **(*S*)-36** (53.0 mg, 0.12 mmol, 37%) as colorless solids.

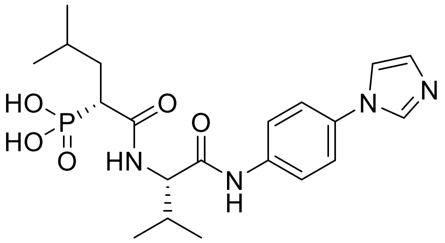

(*R*)-36: ^{1}H NMR (500 MHz, MeOH-d_4) δ: 9.05 (s, 1H), 7.90 – 7.85 (m, 3H), 7.60 – 7.53 (m, 3H), 4.35 (d, *J* = 5.7 Hz, 1H), 3.35 (s, 1H), 2.88 (ddd, *J* = 19.9, 11.7, 3.5 Hz, 1H), 2.34 (dd, *J* = 13.6, 6.8 Hz, 1H), 2.01 – 1.89 (m, 1H), 1.83 – 1.54 (m, 1H), 1.03 (dd, *J* = 12.2, 6.8 Hz, 6H), 0.95 (dd, *J* = 7.8, 6.4 Hz, 6H). ^{13}C NMR (126 MHz, MeOH-d_4) δ: 174.8, 172.8, 140.8, 135.7, 132.5, 123.6, 123.4, 122.7, 121.8, 60.8, 49.9, 39.1, 31.5, 28.2 (d, *J* = 13.8 Hz), 23.8, 21.7, 20.0, 18.1. ^{31}P NMR (202 MHz, MeOH-d_4) δ: 18.5.

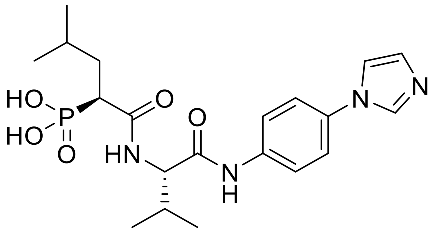

(*S*)-36: ^{1}H NMR (500 MHz, MeOH-d_4) δ: 8.93 (dt, *J* = 14.6, 1.5 Hz, 1H), 7.97 – 7.91 (m, 2H), 7.77 (q, *J* = 1.7 Hz, 1H), 7.57 (t, *J* = 1.7 Hz, 1H), 7.48 – 7.40 (m, 2H), 4.42 (dd, *J* = 4.8, 2.6 Hz, 1H), 3.35 (s, 1H), 3.14 (ddt, *J* = 22.8, 11.3, 2.5 Hz, 1H), 2.50 – 2.40 (m, 1H), 2.20 – 2.10 (m, 1H), 1.66 – 1.59 (m, 1H), 1.03 (dd, *J* = 15.1, 6.9 Hz, 6H), 0.96 (dd, *J* = 6.2, 3.2 Hz, 6H). ^{13}C NMR (126 MHz, MeOH-d_4) δ: 174.9, 173.3, 140.9, 134.9, 131.9, 123.1, 123.0, 122.7, 121.3 (d, *J* = 7.5 Hz), 61.1, 61.1, 47.4, 36.4, 30.8, 28.6 (d, *J* = 14.2 Hz), 23.6, 22.0, 19.8, 17.6. ^{31}P NMR (202 MHz, MeOH-d_4) δ: 18.3. HRMS (ESI+) calculated for $C_{20}H_{30}N_4O_5P$ [M+H]$^+$ 437.1948, found 437.1940.

(4-Methyl-1-(((*S*)-3-methyl-1-((4-morpholinophenyl)amino)-1-oxobutan-2-yl)amino)-1-oxopentan-2-yl)phosphonic acid (37)

According to **GP3**, (2-(diethoxyphosphoryl)-4-methylpentanoyl)-L-valine **26** (110.0 mg, 0.31 mmol) was reacted with 4-morpholinoaniline (61.0 mg, 0.34 mmol), NMM (86 µL, 0.77 mmol) and TBTU (109.0 mg, 0.34 mmol) in DCM (3.1 mL) to afford the diethyl phosphonate dipeptide (159.0 mg, 0.31 mmol, quant) which was used without further purification in the next step. Diethyl phosphonate dipeptide (157.0 mg, 0.31 mmol) was deprotected by bromotrimethylsilane (0.29 mL, 2.17 mmol) in DCM (3.1 mL). Then MeOH was added and stirred at room temperature for 30 minutes to cleave the previously formed TMS ester. The solvents were removed under reduced pressure and the crude product was purified *via* PREP HPLC to afford dipeptide **37** (55.0 mg, 0.12 mmol, 39%) as a colorless solid.

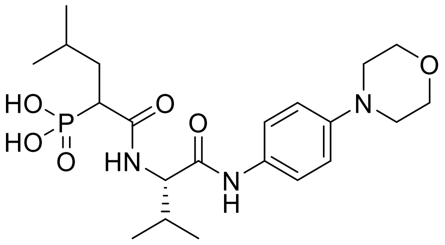

Mixture of diastereomeres. Major diastereomer: ^{1}H NMR (500 MHz, MeOH-d_4) δ: 7.56 – 7.50 (m, 2H), 7.02 – 6.95 (m, 3H), 4.43 (d, *J* = 5.2 Hz, 1H), 3.84 (dt, *J* = 7.8, 3.0 Hz, 4H), 3.21 (ddd, *J* = 23.5, 11.5, 2.6 Hz, 1H), 3.14 (dt, *J* = 7.9, 3.1 Hz, 4H), 2.45 – 2.34 (m, 1H), 2.09 (dddd, *J* = 13.4, 11.6, 7.0, 3.4 Hz, 1H), 1.63 – 1.55 (m, 2H), 1.00 (dd, *J* = 10.3, 6.8 Hz, 6H), 0.95 (d, *J* = 6.3 Hz, 6H). ^{13}C NMR (126 MHz, MeOH-d_4) δ: 172.2, 148.6, 133.0, 123.4, 122.8, 117.8, 67.8, 67.7, 61.1, 60.5, 51.7, 51.5, 47.1, 36.3 (d, *J* = 4.5 Hz), 32.1, 31.2, 28.2 (d, *J* = 14.9 Hz), 23.6, 21.7, 19.8, 17.7. ^{31}P NMR (202 MHz, MeOH-d_4) δ: 22.0. *Minor diastereomer (selected signals*^{1}H NMR (500 MHz, MeOH-d_4) δ: 7.48 – 7.42 (m, 2H), 4.27 (d, *J* = 7.5 Hz, 1H), 3.06 – 2.97 (m, 1H), 2.17 (dt, *J* = 14.2, 7.0 Hz, 1H), 1.99 (dd, *J* = 11.3, 7.7 Hz, 1H). ^{13}C NMR (126

MHz, MeOH-d_4) δ: 172.0, 149.0, 132.8, 123.4, 117.7, 67.7, 59.6, 59.3, 51.6, 46.1, 31.8, 28.0 (d, J = 14.9 Hz), 23.7, 21.8, 18.9. ^{31}P NMR (202 MHz, MeOH-d_4) δ: 22.0. HRMS (ESI+) calculated for $C_{21}H_{35}N_3O_6P$ $[M+H]^+$ 456.2258, found 456.2249.

Tert-butyl _N_-(1-(diethoxyphosphoryl)-3-methylbutyl)carbamate (38)

Followed by reported literature,[2] to a solution of *tert*-butyl carbamate (1.17 g, 10.0 mmol) in anhydrous DCM (20 mL) was added isovaleraldehyde (1.29 mL, 12.0 mmol), and triethylphosphine (1.72 mL, 10.0 mmol). The solution was then treated with copper(II) trifluoromethanesulfonate (360 mg, 1.0 mmol). The mixture was stirred at room temperature for 5 days. The reaction progress was monitored by ^{1}H NMR. After the starting material was completely consumed, the reaction mixture was treated with 1 M HCl and extracted three times with ethyl acetate. The combined organic phases were washed with saturated $NaHCO_3$ and NaCl solutions and dried over Na_2SO_4. After filtration, and removing the solvent, the crude product was purified by column chromatography (SiO_2, PE/EE: 0–100%) to afford title compound **38** (1.29 g, 4.00 mmol, 40% yield) as a colorless solid.

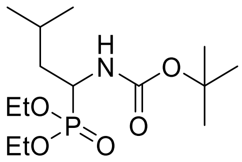

^{1}H NMR (500 MHz, $CDCl_3$) δ: 0.93 (t, J = 6.5 Hz, 6 H), 1.31 (t, J = 7.1 Hz, 6 H), 1.43 (s, 9 H), 1.48 – 1.60 (m, 2 H), 1.68 – 1.77 (m, 1 H), 3.98 – 4.21 (m, 4 H), 4.57 (d, J = 10.5 Hz, 1 H). ^{13}CNMR (126 MHz, $CDCl_3$) δ:16.5, 21.4, 28.4. 38.7, 45.8, 59.8, 62.6, 80.0, 155.3. ^{31}P NMR (202 MHz, $CDCl_3$) δ:26.0.

Methyl-(4-chlorobenzoyl)-L-valine (39a)

To a solution of L-valine methyl ester hydrochloride (335 mg, 2.0 mmol) and triethylamine (0.62 mL, 4.40 mmol) in anhydrous DCM (20 mL), 4-chlorobenzoyl chloride (0.36 mL, 2.80 mmol) was slowly added at 0 °C. The reaction mixture was warmed to room temperature overnight. After the addition of a saturated NH_4Cl solution, the aqueous phase was extracted three times with ethyl acetate. The combined organic phases were washed with a saturated NaCl solution and dried over sodium sulfate. After the solvent was removed under vacuum, the crude product was purified by column chromatography (SiO_2, hexanes/EtOAc gradient 100–85%) to afford compound **39a** (450.0 mg, 1.67 mmol, 83%) as colorless solid.

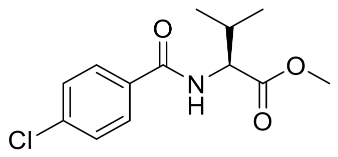

^{1}H NMR (500 MHz, $CDCl_3$) δ: 0.92 (dd, *J* = 10.4, 6.9 Hz, 6H), 2.20 – 2.25 (m, 1H), 3.71 (s, 3H), 4.69 (dd, *J* = 8.6, 4.9 Hz, 1H), 6.56 (d, *J* = 8.7 Hz, 1H), 7.35 (d, *J* = 9.0 Hz, 2H), 7.67 (d, *J* = 8.7 Hz, 2H). ^{13}C NMR (126 MHz, $CDCl_3$) δ: 18.0, 19.0, 27.0, 31.8, 52.3, 57.4, 129.1, 132.5, 138.0, 166.3, 172.6. LCMS: t_R = 4.08 min, m/z = 270 $[M+H]^+$.

Methyl-(4-chlorobenzoyl)-D-valine (40a)

To a solution of D-valine methyl ester hydrochloride (335.0 mg, 2.0 mmol) and triethylamine (0.62 mL, 4.40 mmol,) in anhydrous DCM (20 mL), 4-chlorobenzoyl chloride (0.36 mL, 2.80 mmol) was slowly added at 0 °C. The reaction mixture was warmed to room temperature overnight. After the addition of a saturated NH_4Cl solution, the aqueous phase was extracted three times with ethyl acetate. The combined organic phases were washed with saturated NaCl solution and dried over sodium sulfate. After removal of the solvent under vacuum, the crude product was purified by column chromatography (SiO_2, hexanes/EtOAc gradient 100–85%) to afford compound **39a** (510.0 mg, 1.89 mmol, 89%) as a colorless solid.

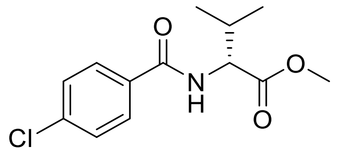

^{1}H NMR (500 MHz, $CDCl_3$) δ: 0.92 (dd, *J* = 10.4, 6.9 Hz, 6H), 2.21 – 2.25 (m, 1H), 3.71 (s, 3H), 4.69 (dd, *J* = 8.6, 4.9 Hz, 1H), 6.56 (d, *J* = 8.7 Hz, 1H), 7.35 (d, *J* = 9.0 Hz, 2H), 7.67 (d, *J* = 8.7 Hz, 2H). ^{13}C NMR (126 MHz, $CDCl_3$) δ: 18.0, 19.0, 27.0, 31.8, 52.3, 57.4, 129.1, 132.5, 138.0, 166.3, 172.6. LCMS: t_R = 4.08 min, m/z = 270 $[M+H]^+$.

(1-((*S*)-2-(4-Chlorobenzamido)-3-methylbutanamido)-3-methylbutyl)phosphonic acid (39)

38 (162.0 mg, 0.50 mmol,) was dissolved in anhydrous DCM (2 mL) and cooled to 0 °C before being treated with HCl (4 M in 1,4-dioxane, 1.50 mL, 6 mmol). The reaction was warmed to room temperature overnight. The reaction progress was monitored by LC-MS. After complete consumption of the starting material, the solvent was removed under vacuum, and the remaining crude product was dissolved in anhydrous DMF (2 mL).

Compound **39a** (81.0 mg, 0.30 mmol) was dissolved in THF (3 mL) and cooled to 0 °C. This solution was treated with LiOH (1 M in water, 0.32 mL, 0.32 mmol) and warmed to room temperature overnight. The reaction progress was monitored by LCMS. After complete conversion, the solvent was removed under vacuum. The crude product was dissolved in anhydrous DMF (2 mL) and cooled to 0 °C. The mixture was then treated with TBTU (110.0 mg, 0.33 mmol) and NMM (81 µL, 0.73 mmol). After stirring the mixture for 30 minutes at room temperature it was cooled to 0 °C and deprotected **38** was added. The mixture was stirred overnight at room temperature. The reaction was quenched by adding a saturated NH_4Cl solution, and the aqueous phase was extracted three times with ethyl acetate. The combined organic phases were washed with saturated NaCl solution and dried over Na_2SO_4. After filtration, and removing the solvent under vacuum, the remaining residue was dissolved in anhydrous DCM (3 mL) and treated with TMSBr (0.50 mL, 3.80 mmol). The reaction progress was monitored by LC-MS. After complete deprotection of the phosphonate, the solution was treated with methanol and stirred for 15 minutes. The solvents were removed under reduced pressure and the crude product was purified *via* a Dionex Ultimate 3000 (Thermo Scientific) with a Nucleodur C18 Graphity column (250mm x 16 mm, particle size 5µm) using UV-detection to afford retro-dipeptide **39** (19.5 mg, 48 µmol, 16%) as a colorless solid.

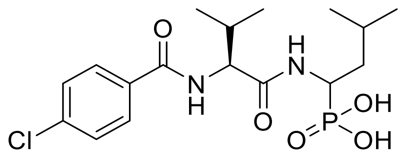

^{1}H NMR (500 MHz, MeOH-*d*$_4$) δ: 0.89 (d, J = 33.0, 6.4 Hz, 6H), 1.04 (dd, *J* = 12.2, 6.7 Hz, 6H), 1.04 (dd, *J* = 12.2 6.7 Hz, 6H), 1.54 – 1.62 (m, 1H), 1.64 – 1.76 (m, H), 2.14 – 2.25 (m, 1 H), 4.24 – 4.45 (m, 1H), 4.32 – 4.44 (m, 1H), 7.74 (d, *J* = 8.4 Hz, 2H), 7.83 (d, *J* = 8.2 Hz, 2H).^{13}C NMR (126 MHz, MeOH-*d*$_4$) δ: 19.2, 19.9, 21.4, 23.8, 32.0, 39.2, 62.2, 129.8, 130.2, 134.2, 138.9, 169.1, 173.4. ^{31}P NMR (202 MHz, MeOH-*d*$_4$) δ: 22.8. HRMS (ESI) calculated for $C_{17}H_{27}ClN_2O_5P$ $[M+H]^+$ 405.1341, found: 405.1305.

(1-((*R*)-2-(4-Chlorobenzamido)-3-methylbutanamido)-3-methylbutyl)phosphonic acid (40)

38 (97.0 mg, 0.30 mmol,) was dissolved in anhydrous DCM (3 mL) and cooled to 0 °C before being treated with HCl (4 M in 1,4-dioxane, 1.0 mL, 4.0 mmol). The reaction was warmed to room temperature overnight. The reaction progress was monitored by LC-MS. After complete consumption of the starting material, the solvent was removed under vacuum, and the remaining crude product was dissolved in anhydrous DMF (2 mL).

40a (81.0 mg, 0.30 mmol) was dissolved in THF (3 mL) and cooled to 0 °C. This solution was treated with LiOH (1M in water, 0.32 mL, 0.32 mmol) and warmed to room temperature overnight. The reaction progress was monitored by LCMS. After complete conversion of the starting material, the solvent was removed under vacuum. The crude product was dissolved in anhydrous DMF (2 mL) and cooled to 0 °C. The mixture was then treated with TBTU (116.0 mg, 0.36 mmol) and NMM (84.0 µL, 0.75 mmol) and stirred for 30 minutes at room temperature. After cooling to 0 °C, the solution containing deprotected compound **38** was added. The mixture was stirred overnight at room temperature and quenched by adding a saturated NH_4Cl solution. The aqueous phase was extracted three times with ethyl acetate. The combined organic phases were washed with saturated NaCl solution and dried over Na_2SO_4. After filtration, and removing the solvent under vacuum, the remaining residue was dissolved in anhydrous DCM (3 mL) and treated with TMSBr (0.45 mL, 3.0 mmol). The reaction progress was monitored by LCMS. After complete deprotection of the phosphonate, the solution was treated with methanol and stirred for 15 minutes. The solvents were removed under reduced pressure and the crude product was purified *via* a Dionex Ultimate 3000 (Thermo Scientific) with a Nucleodur C18 Graphity column (250mm x 16 mm, particle size 5µm) using UV-detection to afford retro-dipeptide **39** (25.0 mg, 62 µmol, 21%) as a colorless solid.

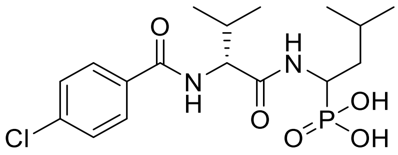

^{1}H NMR (500 MHz, DMSO-d_6) δ: 0.78 (t, J = 7.1, 3H), 0.81 (t, J = 6.7 Hz, 3H), 0.87–0.95 (m, 6H) 1.37 – 1.67 (m, 3H), 2.07 – 2.17 (m, 1H), 4.02 – 4.12 (m, 1H), 4.32 – 4.44 (m, 1H), 7.51 – 7.58 (m, 2H), 7.74 (d, J = 9.6 Hz, 1H), 7.84 – 7.89 (m, 2H), 8.30 (d J = 8.8, 1H).^{13}C NMR (126 MHz, DMSO-d_6) δ: 19.1, 19.9, 21.5, 23.9, 30.8, 45.6, 59.4, 128.8, 129.9, 133.7, 136.5, 165.7, 179.9. ^{31}P NMR (202 MHz, DMSO-d_6) δ: 21.2. LC-MS: t_R = 3.32 min, m/z = 405 $[M+H]^+$.

2. X-ray crystallography

For the crystallization of LasB in complex with the selected inhibitors, the protein was purified from the culture supernatant of PA14 or recombinantly expressed in *E. coli*, as described previously. [3,4] The sequence of the LasB purified from *E. coli* by affinity chromatography corresponds to the LasB sequence of *P. aeruginosa* PAO1, which differs from *P. aeruginosa* PA14 in four positions. It has been shown previously that this LasB variant does not differ in its activity and crystallization properties and that the mutations are not located at the active site.[4] Crystallization trials for compounds **30**, **9**, and **31** were carried out with recombinantly expressed protein and LasB purified from PA supernatant using commercially available screens (NeXtal). LasB was concentrated to ~ 5 mg / mL and incubated with a final concentration of 1 mM compound **30** and **31** in 10 mM Tris pH 8.0 and 2 mM $CaCl_2$ gel filtration buffer for 1 h on ice prior to crystallization. For compound **9**, LasB was concentrated to 15 mg / mL and incubated with 2 mM compound. Screens were prepared in sitting-drop SwissSCI plates using a Gryphon Crystallization robot. After approximately one month of incubation at 291 K, crystals were observed in a variety of conditions. Crystals in complex with compound **30** appeared in a well solution of 0.2 M sodium thiocyanate and 20% (w/v) PEG3350, and LasB co-crystals for compound **31** grew in the condition 0.2 M lithium sulfate, 0.1 M Tris pH 8.5, and 30% (w/v) PEG 4000. Protein crystals for these two compounds were cryo-protected with 32% glycerol, mounted directly from the screen, and were flash-frozen in a cryo-loop. Co-crystals for compound **9** appeared in the optimized condition containing 0.2 M calcium acetate, 0.1 M MES pH 6.5 and 18-26% PEG 8000 and were analogously cryo-protected and flash-frozen. Diffraction data were collected at beamlines ID30B and ID23-1 (ESRF), as well as at beamline P11 at Petra III (DESY)[5] at 100 K. Data were processed using Aimless and Pointless, both implemented in CCP4, and the structure was solved by molecular replacement using PHASER.[6] LasB crystal structures with PDB ID 8CR3, 1EZM, and 6FZX were used as search models.[4,7] The structures were further processed using AutoBuild, manually rebuilt using COOT, and further refined using Phenix.refine.[8]The final refined structures of LasB in complex with compounds **30**, **9**, and **31** were deposited in the Protein Data Bank (PDB) as entries 8R1B, 9FQD, and 9FQY. Images of the co-crystal structures were visualized using PyMOL by Schrödinger (version 2.5.2).

Table S1. Data collection and refinement statistics.

	**LasB+30**	**LasB+9**	**LasB+31**
**PDB code**	8R1B	9FQD	9FQY
**Data collection**			
Space group	$P\ 1\ 2_1\ 1$	$P\ 1\ 2_1\ 1$	$P\ 2_1\ 2_1\ 2_1$
Cell dimensions			
a, *b*, *c* (Å)	39.4, 90.0, 40.8	39.8, 43.7, 81.6	43.6, 51.3, 120.2
α, β, γ (°)	90.0, 114.1, 90.0	90.0, 98.1, 90.0	90.0, 90.0, 90.0
Wavelength (Å)	0.8856	1.0300	0.8856
Resolution (Å)	37.27 – 1.31 (1.36 – 1.31)	38.4 – 1.70 (1.76 – 1.70)	32.0 – 1.60 (1.66 – 1.60)
$CC_{1/2}$	0.997 (0.795)	0.978 (0.731)	0.998 (0.837)
$I / \sigma I$	11.3 (2.1)	13.1 (5.1)	12.3 (2.7)
Completeness (%)	99.6 (97.5)	94.6 (95.3)	99.8 (99.8)
Redundancy	5.9 (5.0)	1.9 (2.0)	7.6 (7.4)
**Refinement**			
Resolution (Å)	37.27 – 1.31	38.4 – 1.70	32.0 – 1.60
No. reflections	61956 (6065)	29200 (2915)	36354 (3564)
R_{work} / R_{free}	0.145 / 0.167	0.206 / 0.244	0.157 / 0.184
No. atoms	2705	2542	2582
Protein	2338	2242	2317
Ligand/ion	78	52	57
Water	316	271	232
B-factors	17.80	13.06	24.34
Protein	15.85	11.97	23.40
Ligand/ion	32.88	12.96	19.78
Water	29.77	22.10	34.35
R.m.s. deviations			
Bond lengths (Å)	0.013	0.007	0.012
Bond angles (°)	1.27	0.92	1.14
MolProbity clash score	3.95	1.76	1.36
Ramachandran (%)			
Favoured	96.96	94.20	96.96
Allowed	2.70	4.78	2.70
Outliers	0.34	1.02	0.34

Statistics for the highest resolution shell are shown in parentheses.

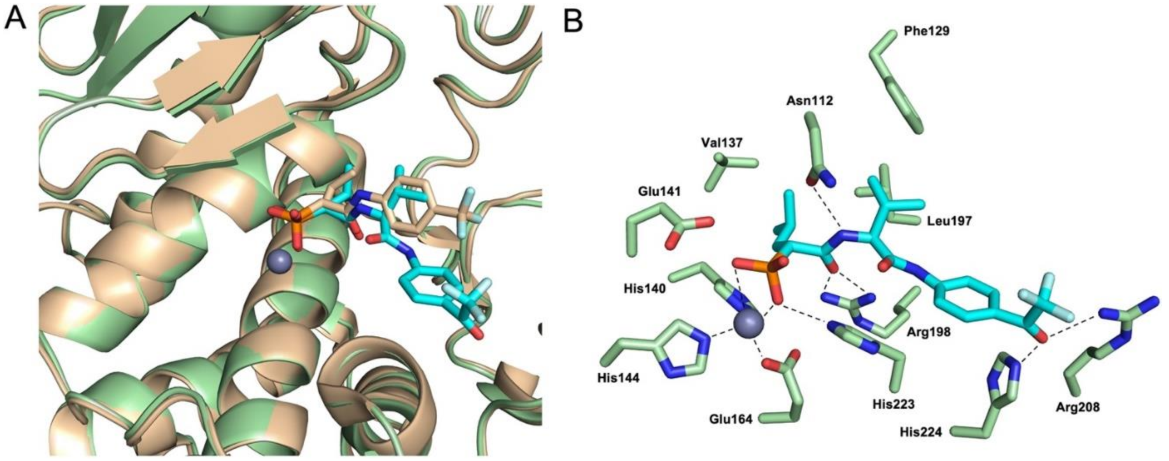

Figure S1. (**A**) Superposition of the crystal structure of LasB (beige) in complex with a phosphonate derivative (beige) (PDB code: 8CC4) and the co-crystal structure of LasB (green) in complex with **31** (cyan). The active-site Zn^{2+} is shown as grey spheres. The valine moiety of **31** perfectly aligns with the aryl substituent of the other phosphonate derivative and occupies the hydrophobic S2' pocket. (**B**) Schematic 2D representation of interactions between LasB and **31**. Additional hydrogen bond interactions with the side chain of His224 and Arg208 compared to the previously published phosphonate derivative are highlighted.

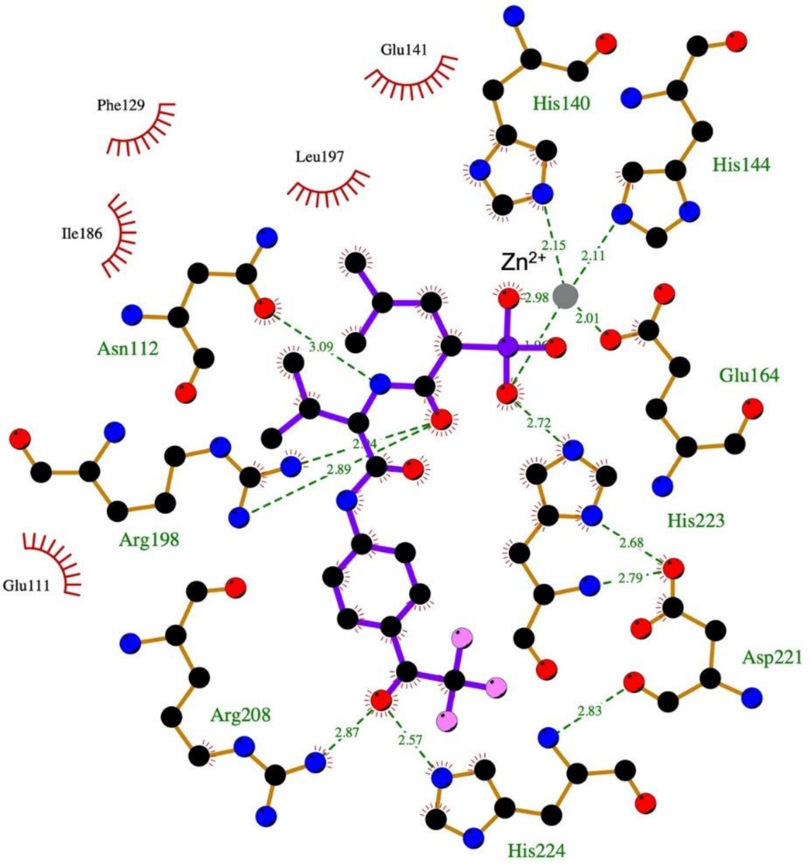

Figure S2. LigPlot+ diagram for the interactions between LasB and **31**.[9] Ligand bonds are represented in purple whereat protein bonds are colored in dark brown. The distance for hydrogen bonds is shown and the interaction is represented as a green, dashed line. Hydrophobic interactions between **31** and LasB are shown as red spoked arcs. The Zn^{2+} cation is represented as a grey sphere.

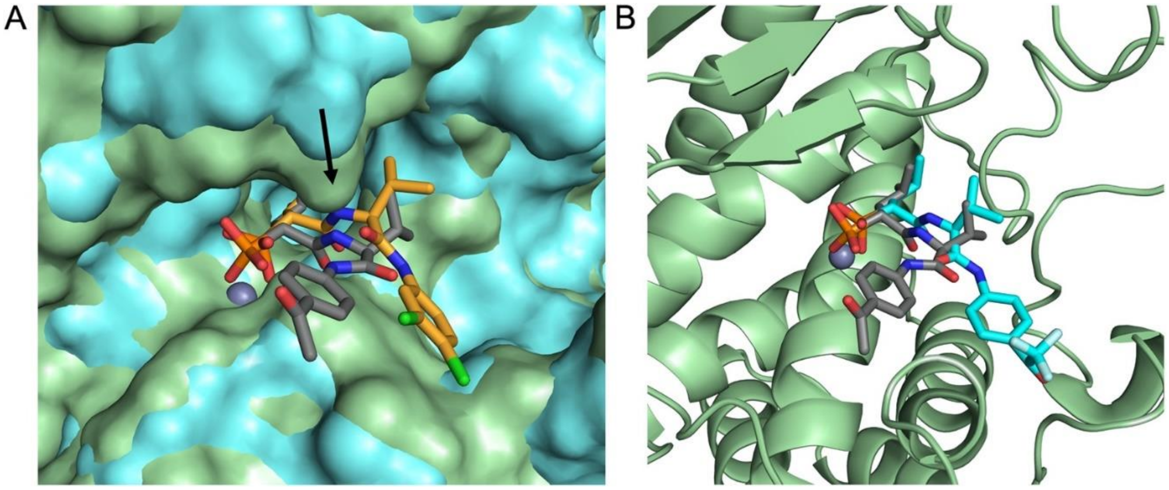

Figure S3. (**A**) Surface representation of the superposed crystal structures of LasB (cyan / green) in complex with **30** (grey) and **9** (orange). LasB adopts a more closed conformation after binding of inhibitor **9** compared to the **30** bound LasB structure (indicated by arrow). (**B**) Cartoon of the superposition of the two LasB (green) crystal structures in complex with **30** (grey) and **31** (cyan). In contrast to **9**, the aryl substituent in **30** is significantly shifted, presumably as a consequence of the intramolecular hydrogen bond between the phosphonate oxygen and the amide nitrogen.

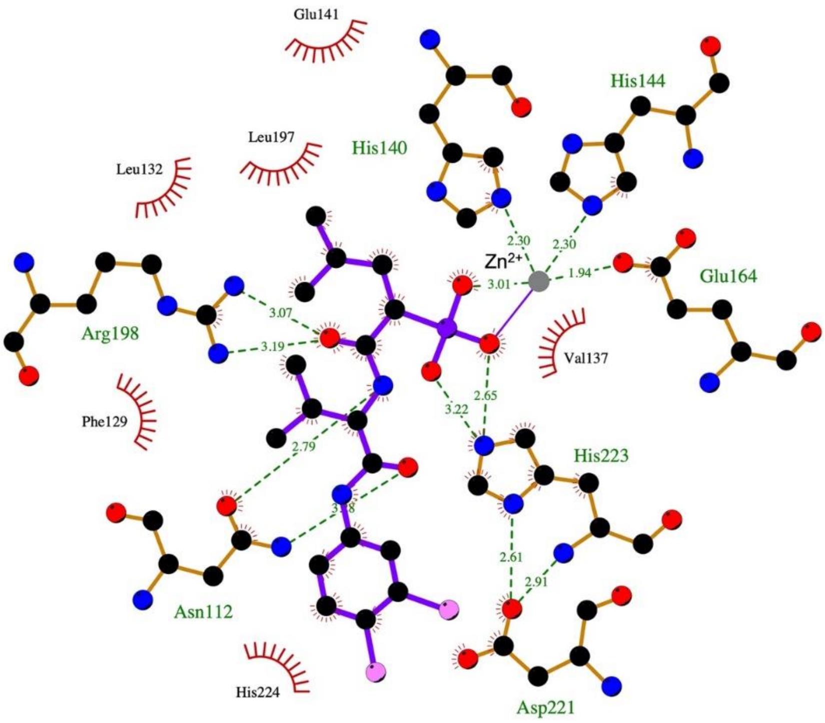

Figure S4. LigPlot+ diagram for the interactions between LasB and **9**. Ligand bonds are represented in purple whereat protein bonds are colored in dark brown. The distance for hydrogen bonds is shown and the interaction is represented as a green, dashed line. Hydrophobic interactions between **9** and LasB are shown as red spoked arcs. The Zn^{2+} cation is represented as a grey sphere.

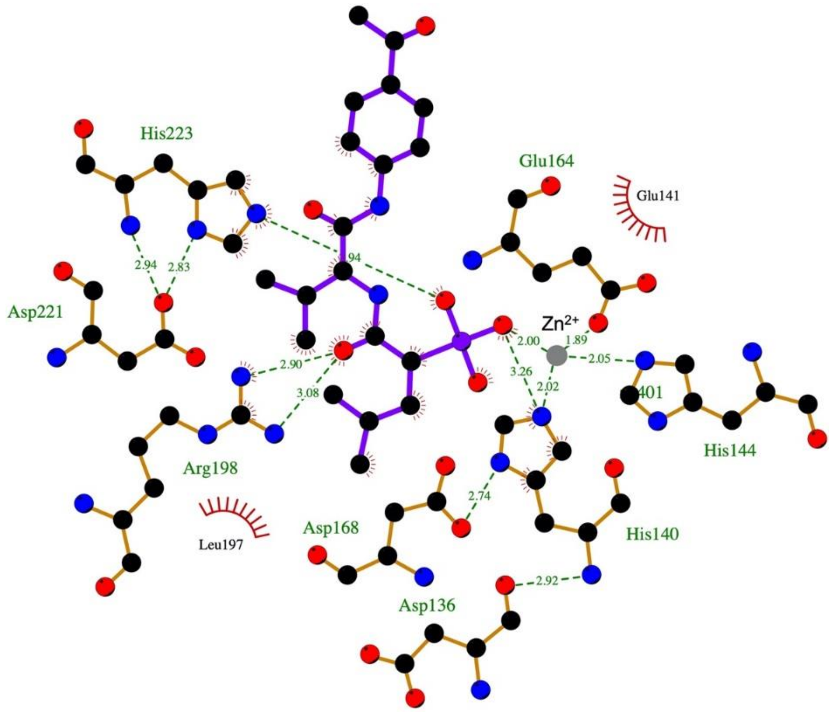

Figure S5. LigPlot+ diagram for the interactions between LasB and **30**. Ligand bonds are represented in purple whereat protein bonds are colored in dark brown. The distance for hydrogen bonds is shown and the interaction is represented as a green, dashed line. Hydrophobic interactions between **30** and LasB are shown as red spoked arcs. The Zn^{2+} cation is represented as a grey sphere.

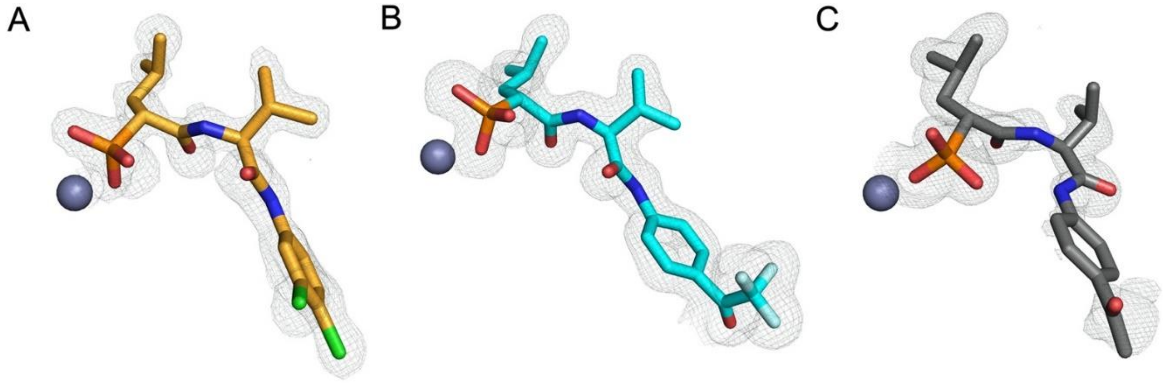

Figure S6. Polder map (grey isomesh) of the compounds (**A**) **9** (orange), (**B**) **31** (cyan) and (**C**) 30 (grey) contoured at a level of 3 σ.

3. Biological evaluation

3.1 In vitro inhibition assays

LasB was expressed and purified as described previously.[3] *In vitro* inhibition assays (LasB, MMPs, TACE). All in vitro inhibition assays were performed as described previously.[10] TACE inhibitor screening kits were purchased from Sigma-Aldrich (Saint Louis, MO). MMPs along with the SensoLyte 520 Generic MMP Activity Kit Fluorimetric were purchased from AnaSpec (Fremont, CA, USA). The assays were performed according to the guidelines of the respective manufacturer. Fluorescence signals were measured using a CLARIOstar plate reader (BMG Labtech, Ortenberg, Germany).

3.2 Kinetic solubility

The desired compounds were sequentially diluted in DMSO in a 96-well plate. 1.5 µL of each well were transferred into another 96-well plate and mixed with 148.5 µL of PBS. Plates were shaken for 5 min at 600 rpm at room temperature (r.t.), and the absorbance at 620 nm was measured. Absorbance values were normalized by blank subtraction and plotted using GraphPad Prism 8.4.2 (GraphPad Software, San Diego, CA, USA). Solubility (S) was determined based on the First X value of AUC function using a threshold of 0.005.

3.3 Lipophilicity determination

$LogD_{7.4}$ was analyzed using an HPLC−based method. The UV retention time of reference compounds with known $LogD_{7.4}$ was determined and plotted toward their $LogD_{7.4}$. Linear regression was used to determine the $LogD_{7.4}$ of unknown compounds. Analysis was performed using a Vanquish Flex HPLC system with variable wavelength detector (Thermo Fisher, Dreieich, Germany) with the following conditions: EC150/2 NUCLEODUR C18 Pyramid column, 5 µM (Macherey Nagel, Düren, Germany); eluent A: 50 mM NH4OAc pH 7.4, eluent B: acetonitrile, and flow: 0.6 mL/min. The gradient was set to 0−100% B from 0 to 2.5 min, 100% B from 2.5 to 3.0 min, 100−0% B from 3.0 to 3.2 min, and 0% B from 3.2−5.0.

3.4 Metabolic stability in liver S9 fractions

For the evaluation of combined phase I and phase II metabolic stability, the compound (1 µM) was incubated with 1 mg/mL pooled mouse liver S9 fraction (C57BL/6, Xenotech, Kansas City, USA) or human liver S9 fraction (Corning, USA), 2 mM NADPH, 1 mM UDPGA, 10 mM MgCl2, 5 mM GSH and 0.1 mM PAPS at 37 °C for 120 min. The metabolic stability of testosterone, verapamil and ketoconazole were determined in parallel to confirm the enzymatic activity of

mouse S9 fractions, for human S9 testosterone, diclofenac and propranolol were used. The incubation was stopped after defined time points by precipitation of aliquots of S9 enzymes with 2 volumes of cold acetonitrile containing internal standard (150 nM diphenhydramine). Samples were stored on ice until the end of the incubation and precipitated protein was removed by centrifugation (15 min, 4 °C, 4,000 g). Concentration of the remaining test compound at the different time points was analyzed by HPLC-MS/MS (TSQ Quantum Access MAX, Thermo Fisher, Dreieich, Germany) and used to determine half-life ($t_{1/2}$) and intrinsic clearance (Cl_{int}).

Species profiling was conducted as above using 0.5 mg/mL pooled mouse, rat or minipig liver microsomes (Xenotech, Kansas City, USA) with 2 mM NADPH and 10 mM $MgCl_2$ and testosterone, verapamil and ketoconazole as reference compounds.

3.5 Plasma stability

To determine stability in plasma, the compound (1 µM) was incubated with pooled CD-1 mouse/human/Wistar rat or Göttingen minipig plasma (Neo Biotech, Nanterre, France). Samples were taken by mixing aliquots with 4 volumes of acetonitrile containing internal standard (125 nM diphenhydramine). The plasma stability of procain, propantheline and diltiazem were determined in parallel to confirm the enzymatic activity. Samples were stored on ice until the end of the incubation and precipitated protein was removed by centrifugation (15 min, 4 °C, 4,000 g, two centrifugation steps). Concentration of the remaining test compound at the different time points was analyzed by HPLC-MS/MS (TSQ Quantum Access MAX, Thermo Fisher, Dreieich, Germany) and used to determine half-life ($t_{1/2}$).

Table S2: Additional in vitro ADMET data for selected dipeptides. n.d.: not determined.

**Compound**	**Kinetic Solubility PBS pH 7.4 [µM]**	**Liver S9 Cl_{int} [µL/mg/min]**		**Plasma $t_{1/2}$ [min]**		**$LogD_{7.4}$**
		**Mouse**	**Human**	**Mouse**	**Human**	
**13**	>200	<5.8	<5.8	>150	>150	–0.58
**14**	>200	<5.8	<5.8	>150	>150	1.14
**(*R*)-30**	>200	<5.8	<5.8	>150	>150	n.d.
**(*R*)-36**	>200	<5.8	<5.8	>150	>150	–0.67

Table S3. Stability of compound **30** in plasma and liver microsomes of the species Wistar rat and Göttingen minipig.

**Compound**	**Mouse**	**Minipig**		**Rat**	
	**Liver Microsomes $t_{1/2}$ [min]**	**Liver Microsomes $t_{1/2}$ [min]**	**Plasma $t_{1/2}$ [min]**	**Liver Microsomes $t_{1/2}$ [min]**	**Plasma $t_{1/2}$ [min]**
**30**	>120	>120	>240	>120	>240

3.6 Calu-3 Permeability

Compound permeability was assessed in vitro with Calu-3 HTB-55 cell line (ATCC). Cells were cultivated in Minimum Essential Medium supplemented with Earle´s salts, L-glutamine, 10% FCS, 1% non-essential amino acids (NEAA) and 1mM sodium pyruvate. Passages between 35 and 55 were used, medium was changed every 2–3 days. For experiments, cells were harvested using trypsin/EDTA and $1x10^5$ cells seeded on Transwell® inserts 3460. Cells were grown in air-liquid interface beginning day 3 and used for transport studies on day 11–13. Transepithelial/transendothelial electrical resistance (TEER) values exceeded 300 Ω*cm^2 before beginning transport studies. For experiments, Krebs-Ringer solution was used and cells were accommodated to the buffer for at least 1 h with no decrease in TEER. 200 µL samples were taken in regular intervals from the apical side (time intervals 0, 15, 30, 60, 120, 180, 300 min) and replenished with fresh buffer. TEER was monitored during the experiment, and epithelial barriers were considered compromised if the TEER fell below 300 Ω*cm^2 during 5 h of experiment duration. A cassette of atenolol, ciprofloxacin and carbamazepine was used as control. Test compounds were applied individually or in cassettes including up to three compounds.

Before analysis, 40 µL of sample was mixed with 80 µL of ice-cold acetonitrile containing internal standard diphenhydramine (150 nM), and the compound concentration was analyzed with HPLC-MS/MS (TSQ Quantum Access MAX, Thermo Fisher, Dreieich, Germany). Samples from the apical compartment taken at the beginning and end of the experiment were diluted 1:10 in KRB + 0.5% DMSO before mixing with acetonitrile.

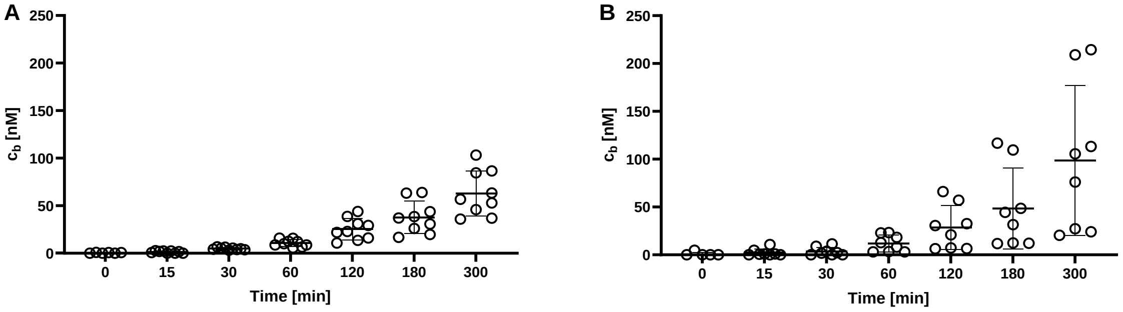

Figure S7: Basolateral concentration of **9** (A) and **30** (B) in the Calu-3 permeability model. Results show individual values, mean and standard deviation of 3 independent measurements with 3 technical replicates each.

3.7 In vivo pharmacokinetic studies

For pharmacokinetic experiments, outbred male CD-1 mice (Charles River, Netherlands), 4 weeks old, were used. The animal studies were conducted in accordance with the recommendations of the European Community (Directive 2010/63/EU, 1st January 2013). All animal procedures were performed in strict accordance with the German regulations of the Society for Laboratory Animal Science (GV-SOLAS) and the European Health Law of the Federation of Laboratory Animal Science Associations (FELASA). Animals were excluded from further analysis if sacrifice was necessary according to the human endpoints established by the ethical board. All experiments were approved by the ethical board of the Niedersächsisches Landesamt für Verbraucherschutz und Lebensmittelsicherheit, Oldenburg, Germany.

30 was subjected to a focused PK study and was administered 30 mg/kg SC. At time points t= 0.25, 0.5, 1, 2, 4, 8 and 24 hours post administration, mice (t = 3 mice per time point) were euthanized and blood was collected from the heart. For all PK studies, whole blood was collected into Eppendorf tubes coated with 0.5 M EDTA and immediately spun down at 13,000 rpm for 10 min at 4°C. The plasma was transferred into a new Eppendorf tube and then stored at -80°C until analysis. Then, a broncheoalveolar lavage was conducted using isotonic sodium chloride solution for all PK studies. For all PK studies, lung, kidney and liver were aseptically removed and homogenized using a Polytron (Kinematica) in isotonic sodium chloride solution. Organ samples were aliquoted into Eppendorf tubes and stored at -80°C until analysis. Moreover, spontaneous urine was also collected.

All PK plasma samples were analyzed via HPLC-MS/MS using an Agilent 1290 Infinity II HPLC system and coupled to an AB Sciex QTrap6500+ mass spectrometer as described previously.[10] Mass spectrometric conditions can be found in Table S5. First, a calibration curve was prepared by spiking different concentrations of **30** into the respective matrix (mouse

plasma (pooled, from CD-1 mice) for plasma samples, isotonic sodium chloride solution for BALF samples, lung tissue for lung samples, kidney tissue for kidney samples, liver tissue for liver samples and urine for urine samples). Caffeine was used as an internal standard. In addition, quality control samples (QCs) were prepared for **30** with the respective matrix. The following extraction procedure was used: 7.5 µl of a plasma sample (calibration samples, QCs or PK samples) was extracted with 37.5 µl of methanol containing 12.5 ng/ml of caffeine as internal standard for 5 min at 2000 rpm on an Eppendorf MixMate® vortex mixer. 10 µl of a urine sample (calibration samples, QCs or PK samples) was extracted with 40 µl of methanol containing 12.5 ng/ml of caffeine as internal standard for 5 min at 2000 rpm on an Eppendorf MixMate® vortex mixer. Then samples (plasma and urine) were spun down at 13.000 rpm for 5 min. Supernatants were transferred to standard HPLC-glass vials. 50 µl of a BALF / lung tissue / kidney tissue or liver tissue sample (calibration samples, QCs or PK samples) were extracted with 50 µl of methanol and 1 µl caffeine (concentration 1 µg/ml in methanol) for 5 min at 800 rpm on an Eppendorf MixMate® vortex mixer. Then samples (BALF, liver, kidney, lung) were spun down at 4,000 rpm for 40 min at 4°C. Supernatants were transferred to 96well V-bottom plates (Greiner). Urea was used to enable calculation of epithelial lining fluid (ELF) concentrations. Peak areas of each sample and of the corresponding internal standard were analyzed using MultiQuant 3.0 software (AB Sciex). Peak areas of the respective sample were normalized to the internal standard peak area. Peaks of PK samples were quantified using the calibration curve. The accuracy of the calibration curve was determined using QCs independently prepared on different days. PK parameters were determined using a non-compartmental analysis with PKSolver.[11] ELF concentrations were calculated using the following formula (2).[12]

(1) $V\_ELF=V\_BALF \times (Urea\_BALF)/(Urea\_Plasma)$

(2) $c\_ELF=c\_BALF\times V\_BALF/V\_ELF$

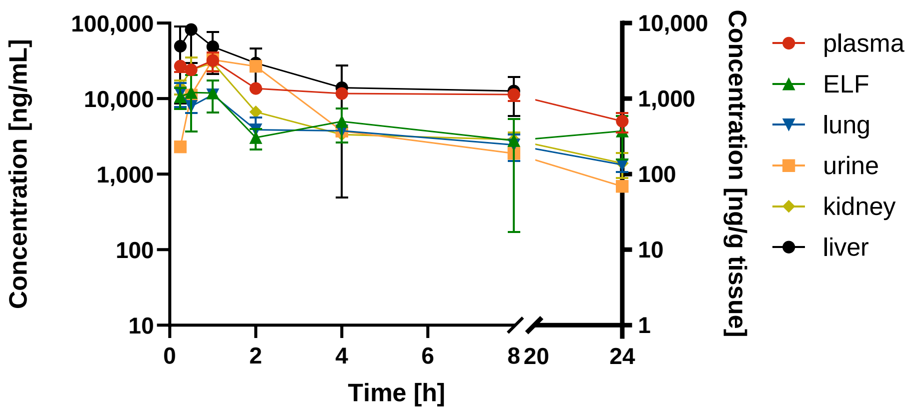

Figure S8: Pharmacokinetic study of **30** applied to mice at 30 mg/kg via subcutaenous administration. Concentrations are given for plasma, epithelial lining fluid (ELF), urine as well as lung, kidney and liver tissue. Corresponding PK parameters are given in Table S7.

Table S4. Plasma pharmacokinetic parameters of **30** after subcutaneous administration to mice (30 mg/kg).

Parameter	Compound 30
$t_{1/2}$ [h]	18.5 ± 11.0
C_{max} [µg/mL]	32.2 ± 8.5
T_{max} [h]	0.75 ± 0.4
$AUC_{0\text{-}t}$ [µg/mL*h]	248.9 ± 15.5
MRT [h]	24.6 ± 16.0
Vz/F_obs [L/kg]	1.9 ± 0.6
Cl/F_obs [mL/min/kg]	1.3 ± 0.3

Table S5: Mass spectrometric conditions. Q1 and Q3 masses for caffeine and **(*R*)-30 (**MS/MS pairs used for quantification are marked with a ‘Q’)

ID	Q1 Mass [Da]	Q3 Mass [Da]	time [msec]	DP [volts]	CE [volts]	CXP [volts]
**Caffeine**	195.024	138.000 (Q)	30.0	130.0	25.0	14.0
		110.000	30.0	130.0	31.0	18.0
**(*R*)-30**	411.125	78.8 (Q)	30.0	–95.0	–82.0	–35.0
		294.9	30.0	–95.0	–40.0	–13.0

3.8 Test against zinc metalloproteases MMP1–3 and TACE (ADAM17), and testing of the Safety Screen44™

We selected a panel of human off-targets, including three human matrix metalloproteases (MMP-1, -2 and -3) and TACE (ADAM-17). These assays were performed as described previously.[3] The TACE inhibitor screening kit was purchased from Sigma-Aldrich (Saint Louis, MO). MMPs along with the SensoLyte 520 Generic MMP Activity Kit Fluorimetric were purchased from AnaSpec (Fremont, CA, USA). The assays were performed according to the guidelines of the respective manufacturer. Fluorescence signals were measured using a CLARIOstar plate reader (BMG Labtech, Ortenberg, Germany).

The off-target Safety Screen44™ was performed by the CRO Eurofins Cerep SA (Celle-L'Evescault, France) according to their protocols.[13]

Table S6: Percent (%) inhibition of three MMPs (1–3) and TACE (ADAM17) in presence of selected LasB inhibitors at 100 µM. Results are means and SD of at least two independent experiments.

**Compound**	**MMP-1** [% inhibition at 100 µM]	**MMP-2** [% inhibition at 100 µM]	**MMP-3** [% inhibition at 100 µM]	**TACE (ADAM17)** [% inhibition at 100 µM]
**9**	13±1	<10	<10	<10
**13**	<10	<10	<10	<10
**14**	<10	<10	<10	<10
**30**	<10	<10	<10	13±4
**(*R*)-30**	<10	<10	<10	13±2
**31**	<10	<10	<10	<10
**(*R*)-36**	<10	<10	<10	<10

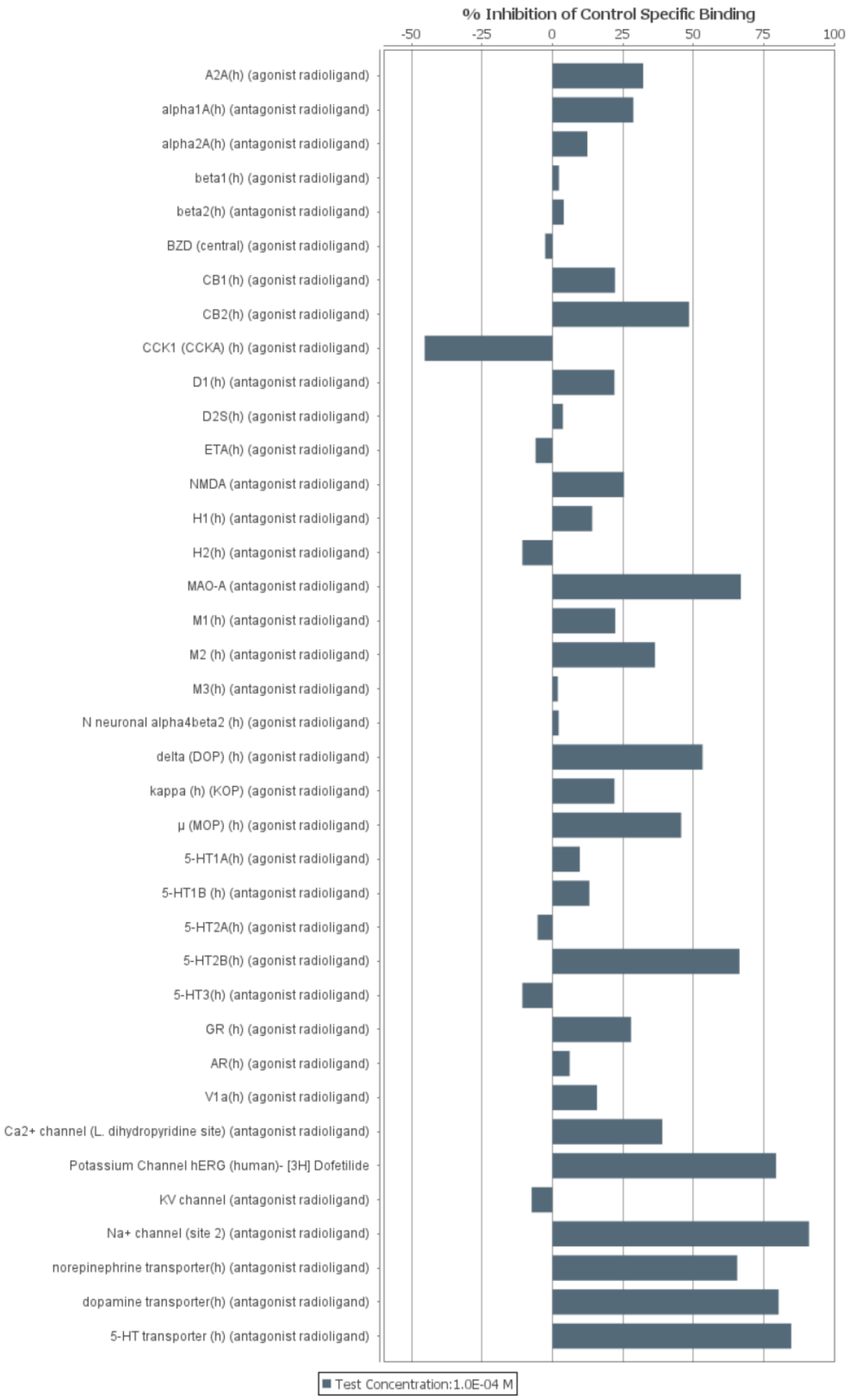

Figure S9. Effect of 100 µM of ***(R)*-30** on the binding of the natural ligands of thirty-eight human off-targets. Percent (%) binding inhibition is shown.

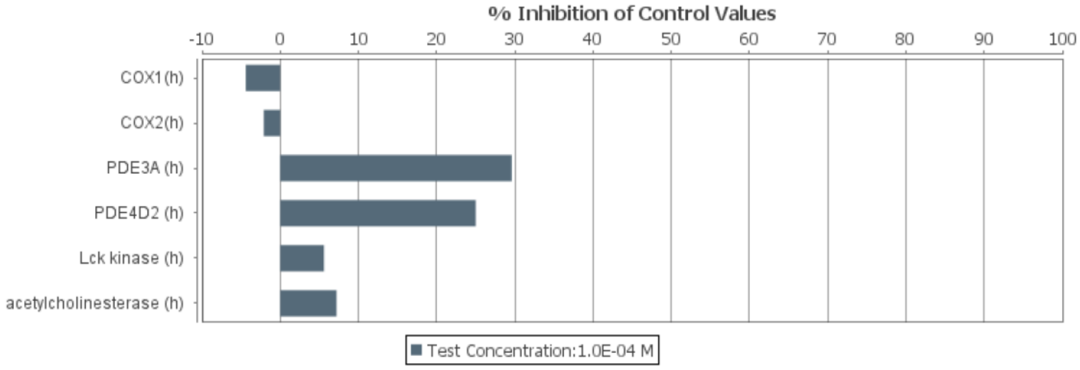

Figure S10. Effect of 100 µM of **(*R*)-30** on the enzymatic activity of six human off-targets.

Table S7. Ten human off-targets whose binding to natural ligands was inhibited by >50% by 100 µM of **(*R*)-30**.

Assay	1.0E-04 M
MAO-A (antagonist radioligand)	66.9%
delta (DOP) *(h)* (agonist radioligand)	53.3%
5-HT_{2B}*(h)* (agonist radioligand)	66.4%
Potassium Channel hERG (human)- [3H] Dofetilide	79.4%
Na^+ channel (site 2) (antagonist radioligand)	91.1%
norepinephrine transporter*(h)* (antagonist radioligand)	65.6%
dopamine transporter*(h)* (antagonist radioligand)	80.3%
5-HT transporter *(h)* (antagonist radioligand)	84.8%
COX1(h)	81.5%
COX2(h)	62.3%

3.9 Cytotoxicity studies

HepG2 (human hepatocellular carcinoma), HEK293 (human embryonal kidney) and A549 (human lung carcinoma) cells ($2x10^5$ cells per well) were seeded in 24-well in flat-bottomed plates. Culturing of cells, incubations and OD measurements were performed as described previously with small modifications.[14] Twenty-four hours after seeding the cells the incubation was started by the addition of compounds in a final DMSO concentration of 1%. The living cell mass was determined after 48 hours in a PHERAstar microplate reader (BMG labtech, Ortenberg, Germany). Two independent measurements were performed for each compound.

Table S8: Percent (%) inhibition of the metabolic activity of three human cell lines HepG2 (hepatocellular carcinoma cells), HEK293 (human embryonic kidney 293 cells) and A549 (lung carcinoma epithelial cells). Results are means and SD of at least two independent experiments.

**Compound**	**HepG2** [% inhibition at 100 µM]	**HEK293** [% inhibition at 100 µM]	**A549** [% inhibition at 100 µM]
**9**	<10	<10	<10
**13**	<10	<10	<10
**14**	13±0	51±4	<10
**30**	<10	<10	<10
**(*R*)-30**	<10	<10	<10
**31**	<10	19±5	<10
**(*R*)-36**	<10	11±9	<10

3.10 Evaluation of Zebrafish embryo toxicity

The experiment was performed according to a procedure described in the literature[15] with minor modifications using zebrafish embryos of the AB wild-type line at 1 day post fertilization (dpf). A detailed protocol has been given in our recent publication.[16]

Table S9: Zebrafish embryo toxicity of compound **30**. Starting exposure ar 2 dpf. Ten larvae were tested for each condition. Dpf: days post fertilization.

Compound	Concentration (µM)	Survival rate (%)			Comment
		3 dpf	4 dpf	5 dpf	
30	100	100	100	100	No observations of concern, e.g., no malformed ZF larvae.
	30	100	100	100	No observations of concern, e.g., no malformed ZF larvae.
	10	100	100	100	No observations of concern, e.g., no malformed ZF larvae.
	2	100	100	100	No observations of concern, e.g., no malformed ZF larvae.

3.11 Tolerability study in male CD1 mice

This in vivo experiment was carried out at the CRO Selvita (Zagreb, Croatia). The study was performed in an AAALAC I- approved Facility. The standard study plan relating to this study was reviewed by the Ethics Committee (CARE- Zagreb) as required by International Laws/Regulations and Croatian Low on Animal Welfare (“The Animal Protection Act”, Official Gazette, NN 135/06 and NN 37/13) and Animal Welfare Officer. Animals were kept in in solid bottom cages (TECNIPLAST S.p A., Italy, Type III polysulphone cage); 425 mm x 266 mm x 180 mm; 3 animals in each cage. The animals were kept on 4 cm thick layer of corn cob grit,

dust-free bedding (Scobis Due - Mucedola, Italy), with a provision of one paper shelters (Lillico Biotechnology, UK). Animals from each group were housed in 1 cage of 2 animals/cage. Mice were housed in the following ambient conditions: temperature 22 ºC ± 2, with relative humidity 55 % ± 10, 15-20 air changes per hour and 12 hours artificial lighting and 12 hours darkness per day (7 a.m. - 7 p.m.).

The experiments were carried out with male Crl: CD1 (IRC) mice purchased from Charles River, Calco (Milano, Italy). Their age was 7-8 weeks at the start of dosing.

This study was carried out with compounds **30** and **(*R*)-30**. As vehicle we used 10% DMSO, 20% Kolliphor EL and 70% PBS. For both compounds, the dose volume was 5 mL/kg (with the exception of 10 mL/kg for the group with the highest dose of 400 mg/kg of **30**). **30** was administered as a single subcutaneous dose. The used doses were 25 mg/kg, 50 mg/kg, 100 mg/kg, 200 mg/kg and 400 mg/kg. Two mice were used per group. **(*R*)-30** was administered intravenously (IV) (TID, 6h intervals) for 3 days followed by 4 days dose-free. Consequently, the compound was administered at 300 mg/kg/day and 600 mg/kg/day for 3 consecutive days. Here, three mice were used per group.

The observations and examinations that were made, comprise clinical signs, body weight, food consumption, macroscopy at termination and organ weights. Mortality was recorded twice a day, *i.e.* in the morning and at the end of the working day.

Table S10: Body weight (gram) after treatment with **30**.

Group Number		Body Weight (g) Day 1	Body Weight (g) Day 4	Body Weight (g) Day 7	Body Weight (g) Day 8
**1 Vehicle**	Mean	38.75	38.95	38.90	36.95
**(SC)**	SD	2.19	2.33	2.26	2.19
	n	2	2	2	2
**2**	Mean	38.70	38.20	37.75	36.35
**25 mg/kg**	SD	0.71	0.99	0.49	0.49
	n	2	2	2	2
**3**	Mean	39.00	39.65	40.10	38.40
**50 mg/kg**	SD	0.85	1.34	1.56	1.27
	n	2	2	2	2
**4**	Mean	38.60	39.15	38.95	35.80
**100 mg/kg**	SD	0.85	0.92	0.92	0.71
	n	2	2	2	2
**5**	Mean	38.50	39.55	39.35	37.25
**200 mg/kg**	SD	0.71	0.49	1.91	0.78
	n	2	2	2	2
**6**	Mean	38.75	39.40	39.35	37.65
**400 mg/kg**	SD	1.06	1.56	1.63	1.48
	n	2	2	2	2

Table S11: Food consumption after treatment with **30**.

Group Number	Total Food consumption g-group		Days 1 - 7 g-animal-day
**1 Vehicle**		Mean	9.16
**(SC)**	110.0	SD	0.00
		n	2
**2**		Mean	8.54
**25 mg/kg**	102.5	SD	0.00
		n	2
**3**		Mean	7.50
**50 mg/kg**	90.0	SD	0.00
		n	2
**4**		Mean	7.30
**100 mg/kg**	88.0	SD	0.00
		n	2
**5**		Mean	7.08
**200 mg/kg**	85.0	SD	0.00
		n	2
**6**		Mean	6.80
**400 mg/kg**	82.0	SD	0.00
		n	2

Table S12: Body and organ weights (gram) after treatment with **30**.

Group Number		Final Body Weight (g)	Brain (g)	Heart (g)	Thymus (g)	Liver (g)	Kidneys (g)	Spleen (g)	Testes (g)	Adrenal glands (g)	Epididym. (g)
**1 Vehicle**	Mean	36.95	0.504	0.200	0.047	1.906	0.714	0.113	0.277	0.021	0.150
**(SC)**	SD	2.19	0.004	0.028	0.001	0.156	0.082	0.001	0.004	0.001	0.025
	n	2	2	2	2	2	2	2	2	2	2
**2**	Mean	36.35	0.489	0.193	0.064	1.763	0.652	0.123	0.322	0.018	0.153
**25 mg/kg**	SD	0.49	0.057	0.004	0.004	0.097	0.037	0.001	0.034	0.001	0.004
	n	2	2	2	2	2	2	2	2	2	2
**3**	Mean	38.40	0.452	0.179	0.060	1.942	0.655	0.121	0.280	0.020	0.129
**50 mg/kg**	SD	1.27	0.025	0.012	0.006	0.098	0.025	0.016	0.008	0.002	0.006
	n	2	2	2	2	2	2	2	2	2	2
**4**	Mean	35.80	0.488	0.175	0.065	1.514	0.554	0.085	0.287	0.022	0.126
**100 mg/kg**	SD	0.50	0.019	0.006	0.004	0.033	0.050	0.004	0.016	0.001	0.004
	n	2	2	2	2	2	2	2	2	2	2
**5**	Mean	37.25	0.481	0.193	0.082	2.175	0.607	0.132	0.249	0.020	0.134
**200 mg/kg**	SD	0.78	0.021	0.003	0.016	0.023	0.029	0.001	0.013	0.004	0.011
	n	2	2	2	2	2	2	2	2	2	2
**6**	Mean	37.65	0.497	0.204	0.075	2.296	0.688	0.121	0.292	0.026	0.139
**400 mg/kg**	SD	1.05	0.022	0.011	0.021	0.181	0.088	0.006	0.020	0.000	0.004
	n	2	2	2	2	2	2	2	2	2	2

Table S13: Body weight (gram) after treatment with ***(R)*-30**.

Group Number		Body Weight (g) Day 1	Body Weight (g) Day 2	Body Weight (g) Day 3	Body Weight (g) Day 4	Body Weight (g) Day 5	Body Weight (g) Day 6	Body Weight (g) Day 7
**1 Vehicle**	Mean	36.47	35.37	34.97	34.87	35.40	35.70	35.73
	SD	2.51	3.36	3.65	3.44	2.99	3.12	3.15
	n	3	3	3	3	3	3	3
**4**	Mean	33.50	34.27	34.27	34.33	34.70	34.97	35.03
**300 mg/kg/day**	SD	0.26	0.31	0.06	0.15	0.36	0.49	0.38
	n	3	3	3	3	3	3	3

Group Number		Body Weight (g) Day 1	Body Weight (g) Day 2	Body Weight (g) Day 3	Body Weight (g) Day 4	Body Weight (g) Day 5	Body Weight (g) Day 6	Body Weight (g) Day 7
**1 Vehicle**	Mean	35.20	35.27	34.97	35.30	35.30	35.00	35.50
	SD	3.20	3.28	3.25	2.93	3.00	3.24	2.74
	n	3	3	3	3	3	3	3
**7**	Mean	36.63	36.27	37.23	37.70	37.17	37.60	37.90
**600 mg/kg/day**	SD	1.33	1.07	1.14	1.01	1.56	1.68	1.90
	n	3	3	3	3	3	3	3

Table S14: Food consumption after treatment with **(*R*)-30**.

Group Number	Total Food consumption g-group		Days 1 - 7 g-animal-day
1 Vehicle	95	Mean	5.28
		SD	0.00
		n	3
4 300 mg/kg/day	101	Mean	5.61
		SD	0.00
		n	3

Group Number	Total Food consumption g-group		Days 1 - 7 g-animal-day
1 Vehicle	81	Mean	4.50
		SD	0.00
		n	3
7 600 mg/kg/day	85	Mean	4.72
		SD	0.00
		n	3

Table S15: Body and organ weights (gram) after treatment with **(*R*)-30**.

Group Number		Final Body Weight (g)	Brain (g)	Heart (g)	Thymus (g)	Liver (g)	Kidneys (g)	Spleen (g)	Testes (g)	Adrenal glands (g)	Epididym. (g)
1 Vehicle	Mean	35.50	0.505	0.192	0.068	1.858	0.504	0.113	0.226	0.025	0.110
	SD	2.74	0.014	0.036	0.012	0.105	0.095	0.017	0.026	0.006	0.010
	n	3	3	3	3	3	3	3	3	3	3
300 mg/kg/day	Mean	35.03	0.499	0.173	0.067	2.138	0.647	0.136	0.249	0.021	0.125
	SD	0.38	0.025	0.011	0.016	0.054	0.078	0.021	0.027	0.005	0.030
	n	3	3	3	3	3	3	3	3	3	3
600 mg/kg/day	Mean	37.90	0.488	0.183	0.060	2.428	0.559	0.174	0.281	0.022	0.117
	SD	1.90	0.030	0.016	0.017	0.178	0.042	0.022	0.052	0.007	0.015
	n	3	3	3	3	3	3	3	3	3	3

3.12 Antibacterial effects

As start OD_{600} of *Pseudomonas aeruginosa* strain PA14 we used 0.03 in a total volume of 200 µL in lysogeny broth containing the compounds predissolved in DMSO (max. 1%). Final compound concentrations prepared from serial dilutions ranged from 3.125 to 100 µM (double values for each concentration). The ODs were determined after addition of the compounds and again after incubation for 18 h at 37 °C and 200 rpm in 96 well plates (Sarstedt, Nümbrecht, Germany) using a FLUOStar Omega (BMG labtech, Ortenberg, Germany). Given percent (%) inhibition values are means of at least two independent determinations. To obtain the values for a given compound, their ODs were compared to those of DMSO controls. Concerning the

growth curves with *Pseudomonas aeruginosa* strain PA54, a start OD_{600} of 0.03 in a total volume of 200 µL was used. This volume contained 1% of DMSO in all samples. As growth media we used either pure LB medium, or LB supplemented with 10 or 50% of citric acid buffer (pH 7.4, 300 mOsm/L). The OD_{600} was determined every 10 min for 10 or 18 h at either 25 °C or 37 °C, respectively, with permanent shaking in 96 well plates using a FLUOStar Omega.

Table S16: Percent (%) inhibition of the growth of *Pseudomonas aeruginosa* strain PA14 after challenge with 100 µM of seven compounds. Results are means and SD of at least two independent experiments.

**Compound**	**P. aeruginosa PA14** [% inhibition at 100 µM]
**9**	<10
**13**	<10
**14**	<10
**30**	<10
**(*R*)-30**	<10
**31**	<10
**(*R*)-36**	<10

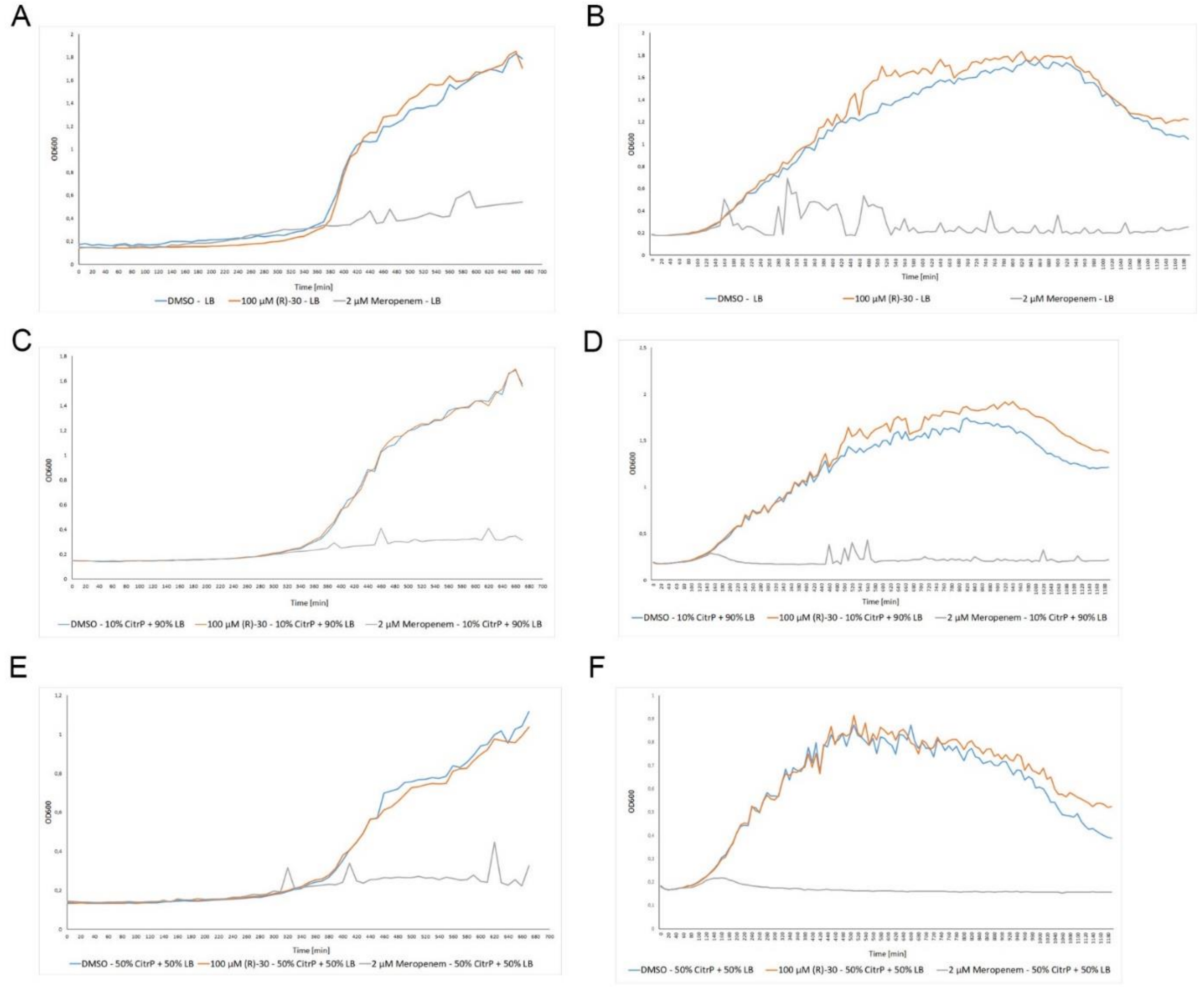

Figure S11: Growth curves of *Pseudomonas aeruginosa* PA54. Bacteria were either treated with DMSO (blue curves), **(*R*)-30** (orange) or meropenem (grey). The cell densities were determined every 10 min after incubation at either 25 °C (A, C, E) or 37 °C (B, D, F). The bacteria were grown in either pure LB medium (A, B), LB medium containing 10% isotonic citric acid buffer (C, D) or LB medium containing 50% isotonic citric acid buffer (E, F).

3.13 Inhibition of biofilm formation

Biofilm formation was determined using a MBEC Assay® Biofilm Inoculator with 96 well base (Innvotech, Edmonton, CA). An overnight culture of PA14 was centrifuged (10 min, 5000 x g). The supernatant including extracellular polymers was removed and the pellet washed with 10 mL of LB medium. Centrifugation was repeated, the supernatant discarded and the pellet resuspended in 5 mL of LB. OD was adjusted to 0.2. The bacteria were further diluted in LB medium, followed by addition of **30** or DMSO control to give OD 0.1, 100 µM **30** and 1% DMSO. 150 µL were added to each well of the 96 well plate. The outer wells were used as blanks containing 150 µL of 1% DMSO in LB. Four replicates per sample were distributed evenly across the plate, with every row containing one replicate of each sample. The well plate was

covered with the peg lid. One layer of parafilm was wrapped around the biofilm inoculator. The inoculator was incubated at 37°C, static for 24 with a beaker of H_2O ensuring humidity.
After 24 h, the plate was removed from the incubator and the peg lid was transferred to a fresh 96 well plate (Nunc U bottom, Thermo Fisher, Dreieich, Germany) containing DMSO or 100 µM **30** in 150 µL 1% DMSO/LB. The inoculator was wrapped with parafilm again and incubated for another 24 h. After that, the peg lid was transferred for 1 min to a 96 well plate containing 200 µL sterile MΩ in each well, followed by another 96 well plate containing 200 µL LB in each well. The system was sealed with parafilm and sonicated for 15 min in an ultrasonic bath with tray. Immediately after sonication, 20 µL were removed from each well using a multi-channel pipette and diluted 1:10 (stepwise, until 10^{-6}). After each dilution step, 10 µL were directly transferred to cetrimide agar plates (spot plating) using fresh pipette tips. Agar plates were incubated for 16 h at 37°C and then used for cell counting. Results are shown in Figure S12.

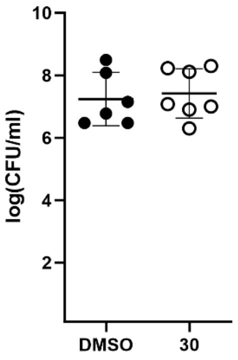

Figure S12: Colony-forming units (CFU) of *P. aeruginosa* PAO1 in biofilms after 48 h growth in presence of 100 µM **30** or DMSO as measured using the Calgary biofilm device.[17] Biofilms were grown for 48 h in presence of DMSO/**30** from the moment of biofilm inoculation. Medium was replaced after 24 h by fresh medium containing DMSO/**30**. Mean ± SD and individual values from 2 independent experiments are displayed.

3.14 In vivo target engagement studies using *Pseudomonas* keratitis model

Evaluation of *lasB* gene expression in *Pseudomonas aeruginosa* strain PA54 through relative and absolute quantification using quantitative polymerase chain reaction (qPCR): Overnight bacterial cultures of *Pseudomonas aeruginosa* strains PA54, PAO1 (DSM 19880) and PA14 (DSM 19882) were prepared in LB medium. The following day, cell lysates were prepared by adding 2 mL of QIAGEN RNAprotect® Bacteria Reagent to 1 mL of culture adjusted to an OD_{600} of 1. These mixtures were vortexed and left at room temperature for 5 min, followed by a 10 min centrifugation at 5000x g at room temperature. The resulting supernatant was removed, and the pellet was re-suspended in 20 µL of Proteinase K previously mixed with 200 µL of TE buffer (containing 30 mM Tris-Cl, 1 mM EDTA, pH 8.0, and 15 mg/mL lysozyme).

This mixture was vortexed for 10 seconds every 2 minutes over a 10-minute period. Subsequently, 700 µL of buffer RLT (provided in the QIAGEN RNeasy Plus Kit) was added to each tube and vigorously vortexed. The flow-through was then transferred to the gDNA eliminator spin column (also provided in the kit) and centrifuged for 2 minutes at 14000 rpm at room temperature. After discarding the column, 700 µL of 99% ethanol was added to the flow-through of each sample.

The next step involved RNA extraction and purification using the QIAGEN RNeasy Plus Kit, with slight modifications to the manufacturer's instructions. Centrifugation times were increased from 15 seconds to 2 minutes, and after eluting the RNA with RNase-free water, samples were treated with the Invitrogen™ DNA-free™ DNA Removal Kit to ensure the complete removal of genomic DNA. For each sample, 8 µL of DNase I buffer was combined with 2 µL of rDNase I, followed by a 25-minute incubation at 37°C. Subsequently, 10 µL of DNase inactivation reagent was added and occasionally mixed for 2 minutes at room temperature. Finally, all samples were centrifuged at 10000x g for 1.5 minutes, and the supernatant containing purified RNA was transferred to a new tube. RNA concentration was measured using a NanoDrop 2000 Spectrophotometer (Thermo Fisher Scientific), and 100 ng of mRNA was used for reverse transcription.

To generate single-stranded cDNA from the purified RNA, the Applied Biosystems™ High-Capacity cDNA Reverse Transcription Kit was employed. The reverse transcription process was conducted according to the manufacturer's instructions, and the final cDNA was used for gene expression analysis. For qPCR, TaqMan™ Fast Advanced Master Mix (Applied Biosystems™) was used, and the qPCR reaction plate was prepared following the manufacturer's instructions, with a final primer concentration of 900 nM and a probe concentration of 250 nM. Primers and Probes specific to *lasB* were designed using Geneious Prime software (Table S16). To ensure there was no contamination and no signal from genomic DNA, a no template control (NTC) and a no reverse transcriptase control (-RT) were included. The gBlocks™ Gene Fragment (Integrated DNA Technologies) for absolute quantification was prepared by adding nuclease-free water to the tube to achieve a concentration of 10 ng/µL, followed by a 20-minute incubation at 50°C. The mixture was then serially diluted by a factor of 5 and amplified alongside the samples using the same primers and probe employed for *lasB* to generate the standard curve. The StepOnePlus™ Real-Time PCR System (Applied Biosystems™) was used for the qPCR run, with thermal cycling conditions according to the TaqMan™ Fast Advanced Master Mix instructions.

The analysis of fold change and copy numbers was conducted through Microsoft Excel, and the data were graphically represented using GraphPad Prism 9.

Table S17: Primers and probes applied in this study (designs were carried out using Geneious Prime bioinformatics software)

Gene	Forward primer	Reverse Primer	Probe	Description
lasB	CATCACCGTCGACATGAACA	AGAAATGCGCGTCGTTCAG	CGCTTCGCCTGCCCGACCAA	*Pseudomonas aeruginosa* Elastase B
rpoS	CTGAACGAACGGGTGACTTC	TCGCTGAGATCGTCATCCTG	ACCGACGATCGCCCCACCGA	RNA polymerase sigma factor RpoS
rho	CAGATCCGGCGCTTCAAC	TCTCCGGACGATCGAAGTTG	GCACGGGCGACACCATCATCGG	Transcription termination factor Rho

Animals: The animal experiments were approved by Saarland University's Animal Welfare Committee (Application number: 17/2021) and conducted in accordance with German and European recommendations and guidelines for the ethical use of animals. The laboratory animals were all C57BL/6N female eight to eleven months old mice. Keeping in specific pathogen-free conditions was provided. Unlimited access to water and show was secured. Temperature was closely maintained around 22 °C and relative air humidity around 50%. Light and darkness alternated at twelve-hour intervals. All laboratory animals were purchased from Charles River GmbH, Sulzfeld, Germany.

P. aeruginosa: The *P. aeruginosa* strain PA54 was employed to induce an experimental bacterial keratitis in the aforementioned C57BL/6N mice. The strain PA54 was isolated from human infections and collected at Saarland University Medical Center's Institute for Medical Microbiology and Hygienics (IMMH) in 2009. According to Magiorakos *et al*., PA54 can be classified as multidrug-resistant due to its lacking sensibility to the anti-pseudomonal cephalosporins ceftazidime and cefepime, the anti-pseudomonal fluoroquinolones ciprofloxacin and levofloxacin, the phosphoric acid fosfomycin, and the aminoglycoside gentamicin.[18] The minimum inhibitory concentration (MIC) of the reserve antibiotic meropenem (meropenem trihydrate, Sigma) was 1 µg/mL (sensitivity limit = 2 µg/mL). The *exoU*- and *exoT*-positivity of the PA54 strain, foregrounds its keratitogenic potential.

Inoculum: The *P. aeruginosa* strain PA54 is routinely grown overnight on blood agar (TrypticaseTM Soy Agar, 5% sheep blood, BD, Heidelberg, Germany) at 37 °C. To obtain a stationary phase cell inoculum, a freshly grown colony was inoculated in tryptic soy broth (TSB; BD, Heidelberg, Germany) and cultured at 37 °C and 150 rounds per minute (rpm) for 16 h. Then, 2 mL were centrifuged at 13,200 rpm at rt for 2 min, whereupon cells were resuspended in spent medium to an OD_{600} of 10 (~1×10^{10} CFU/mL). Short storage on ice followed, before the infection procedure started.

Infection Procedure: Anesthesia and infection were performed as previously published.[19] Briefly, fentanyl (Hameln Pharma Plus GmbH, Hameln, Germany), midazolam hydrochloride (Midazolam-Hameln, 5 mg/mL, Hameln pharma GmbH, Germany) and medetomidine hydrochloride (Domitor, Orion Corporation, Espoo, Finland) were dissolved in 0.9% NaCl (NaCl 0.9%, Ecotainer 500 mL, B. Braun, Melsungen, Germany), dosed at 0.05 mg/kg, 5 mg/kg and 0.5 mg/kg body weight, respectively, and applied by intraperitoneal injection. Analgesia (Carprofen, 5 mg/kg body weight, Zoetis Deutschland GmbH, Berlin, Germany) was applied subcutaneously. Lacrimation was stopped with 10 µL of a 0.6% acetylcysteine solution (Pharmacy of Saarland University Hospitals, Homburg, Germany). Local analgesia (0.5% proxymetacaine hydrochloride, Proparakain-POS Augentropfen, Ursapharm Arzneimittel GmbH, Saarbrücken, Germany) was eye-dropped and removed after 60 seconds. The blunt side of a scalpel was used to pass five times over the cornea to remove the superficial epithelium. Then, three parallel, vertical, 1–2 mm long scratches were added to the cornea with a PA54-loaded 27-Gauge needle (B. Braun). Afterwards, 5 µL (~5×10^{7} CFU/mL) of the inoculum were pipetted onto the scratched eye and left untouched for 20 min (Preston et al., 1995; Wu et al., 2017). Finally, 1.2 mg/kg body weight naloxone hydrochloride (Naloxon Inresa, 0.4 mg/mL, Inresa Arzneimittel GmbH, Freiburg, Germany), 0.5 mg/kg flumazenil (Flumazenil Inresa 0.5 mg, 0.1 mg/mL, Inresa Arzneimittel GmbH) and 2.5 mg/kg atipamezole hydrochloride (Antisedan, 5 mg/ml, Vetoquinol GmbH, Ismaning, Germany) were dissolved in 0.9% NaCl and injected subcutaneously, awakening the mouse.

Documentation and Treatment: Documentation was performed every 24 h. Keratitis severity was visualized using a stereomicroscope (Leitz ELVAR, Wetzlar, Germany) at 16× magnification combined with a high-resolution microscope digital camera (Seben GmbH, Berlin, Germany) and evaluated using a previously described clinical scoring scheme (Lyu et al., 2020). Briefly, a score of 0 indicates no infiltrates, 1 indicates that less than 50% of the cornea is semitransparent, 2 indicates that more than 50% of the cornea is semitransparent, 3 indicates that less than 50% of the cornea is opaque, 4 indicates that more than 50% of the cornea is opaque, and 5 indicates that either spontaneous corneal perforation or phthisis bulbi

has occurred. The body weight of the mice was measured daily and used as welfare indicator. General condition and behavior were also recorded daily. Treatment was performed every eight hours starting six hours after infection along 72h. Anesthesia was reached using 3% isoflurane inhalation (Isoflurane-Piramal, Piramal Critical Care Deutschland GmbH, Halbergmoss, Germany) under stable oxygen supply (1.0–1.2 L/min). Then a volume of 5 µL of the appropriate substance was applied to the infected eye. A two-minute incubation period followed. The animal was finally reawakened by isoflurane-removal. Four treatment groups were created. The sham treatment group was applied phosphate buffered saline containing 1% DMSO. The meropenem treatment group was applied a 0.9% NaCl solution containing 500 µg meropenem/mL and 1% DMSO. The LasB-inhibitor treatment group was applied a 0.9% NaCl solution containing 1 mg LasB-inhibitor/mL and 1% DMSO. The combination treatment group was applied a 0.9% NaCl solution containing 1 mg LasB-inhibitor/mL, 500 µg meropenem/mL, and 1% DMSO.

Animal Sacrifice: Laboratory animals were sacrificed by intraperitoneal injection of ketamine hydrochloride/xylazine hydrochloride (Ursotamine, 100 mg/mL, Serumwerk Bernburg AG, Germany; Rompun 2%, xylazine, 25 ml, Bayer AG) at a dose of 20 mg/kg body weight three days after infection. The vena cava served was punctured to collect blood. Anticoagulation was achieved by Ca^{2+}-deprivation using a drop of 0.5 M ethylenediaminetetraacetic acid (EDTA). The right and initially infected eye was removed by incising the surrounding skin if necessary.

Eye Homogenization: The removed eyes were homogenized using a POLYTRON PT 1200 E dispenser (Kinematika AG, Lucerne, Switzerland). To avoid bacterial carry-over, the eyes were cleaned with distilled water, 70% ethanol and 0.9% NaCl solution, respectively.

Microbiological Evaluation: Serial dilutions were plated on blood agar for microbiological evaluation of the homogenates. A blood stripe also incubated on blood agar served as a control for potential systemic spread of the pathogen. In both cases, counting was performed after 18h of incubation at 37°C.

Flow Cytometry: The eye homogenates, upon receipt, were sieved using a 70 µm cell filter (Falcon, Corning) to prepare a single cell suspension. Total cell count and cell viability were determined using a NucleoCounter® NC-200™ (Chemomatec, Kaiserslautern, Germany). Single cell suspensions were fixed using 1% paraformaldehyde, centrifuged, and resuspended in FACS buffer (0.1mM EDTA plus 1% fetal bovine serum in PBS). CD16/CD32 antibodies were then added to the samples and incubated at 4 °C for 40 min to block Fc receptors. The surface markers were stained using the following fluorescent dye-conjugated antibodies:

F4/80-PE (clone BM8), CD45- APC (clone 30-F11), CD11b-FITC (clone M1/70), CD11c APC-Cy7 (clone N418), Ly6G PE-Cy7 (1A8-Ly6g), and NK1.1-PerCP-Cy5.5 (clone PK136) (Biolegend, Amsterdam, Netherlands) and incubated at 4 °C for 45 min. The blood samples were first mixed with an ACK-Lysis Buffer (Thermo Fisher Scientific, Braunschweig, Germany) for 20 min. The lysis was terminated in time by adding DPBS. Centrifugation was used to selectively collect the leukocytes. Total cell count estimation and surface marker staining were performed as described above for the eye samples. The BD FACSverse Cell analyzer machine was used for flow cytometry analysis, whereas the resulting data were analyzed using FlowJo Software **(**Version 10.6.2; Becton, Dickinson, and Company (BD), Heidelberg, Germany**).**

Enzyme-linked Immunoadsorption Assay (ELISA): After centrifugation of the homogenates at 2500 g and 4 °C for 10 min, the cell-free suspension was frozen at –70 °C. Quantification of mKC, mTNF-α, mMPO and mIL-1β in the centrifuged homogenate was performed using R&D ELISA kits according to the manufacturer's protocol. Briefly, 96-well microplates were coated with the respective capture antibody and incubated at rt overnight. The following day, the wells were blocked with reagent diluent (1% BSA solution) for 2h, incubated with the samples for 2h, incubated with the detection antibody for 2h and finally incubated with streptavidin-HRP for 20 min. The reaction was started by adding the BM Blue POD substrate solution and stopped after 20 min with 2N H_2SO_4. The absorbance was measured at 450 nm. A serially diluted standard was used in parallel to calculate the concentration of each protein.

Statistical Analysis: Statistical analysis was performed using the GraphPad Prism Software Package (Version 10.0.2). Pairwise comparison for connected and not-connected samples was done with the non-parametric Mann-Whitney *U* test and with the Wilcoxon-signed-rank-test respectively. The non-parametric Friedman test was employed for multiple comparisons for connected samples. The *P*-value was two-sided and considered statistically significant when < 0.05.

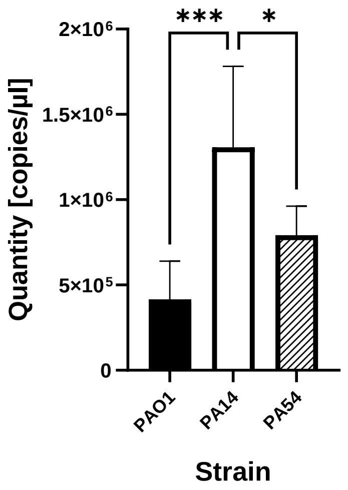

Figure S13: Gene expression analysis of *lasB* in *Pseudomonas aeruginosa* strains PAO1, PA14, and PA54 cultivated in LB medium. Absolute quantification of *lasB* expression in using the standard curve method. These results represent the average of 3 independent experiments ± SD. The ordinary one-way ANOVA was performed, comparing the mean values of PAO1 and PA54 with the highly virulent isolate PA14. (ns = not significant, * = p ≤ 0.05, *** = p ≤ 0.001). Data were plotted using GraphPad Prism 9.

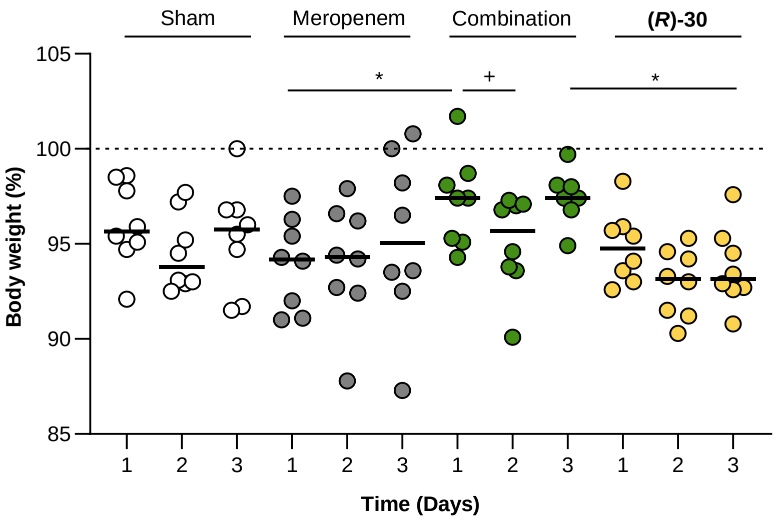

Figure S14: Impact of the PA54 corneal infection and treatment on the body weight over time. Percentages (%) are the percentage of the initial body weight. Aged mice (*n* = 8 per group) were infected with PA strain PA54 and treated for every 8 h with the treatment indicated for up to 72 h. Data represent the values of every individual animal (symbols) and the median (horizontal line). *, *P* < 0.05 (Mann-Whitney *U* test between two independent groups) +, *P* < 0.05 (Wilcoxon signed-rank test between different time points within a group).

3.15 Bioanalytical sample preparation of keratitis samples for determination of concentration of (*R*)-30 in eye and serum

All PD serum and eye homogenate samples were analyzed via HPLC-MS/MS using an Agilent 1290 Infinity II HPLC system and coupled to an AB Sciex QTrap6500+ mass spectrometer. First, a calibration curve was prepared by spiking different concentrations of **(*R*)-30** into the respective matrix (mouse serum (pooled, from CD-1 mice) for plasma samples, isotonic sodium chloride solution for eye homogenate samples). Caffeine was used as an internal standard. In addition, quality control samples (QCs) were prepared for **(*R*)-30** with the respective matrix. The following extraction procedure was used: 7.5 µL of a serum sample (calibration samples, QCs or PD samples) was extracted with 37.5 µL of methanol containing 12.5 ng/mL of caffeine as internal standard for 5 min at 2,000 rpm on an Eppendorf MixMate® vortex mixer. Then samples (serum) were spun down at 13,000 rpm for 5 min. Supernatants were transferred to standard HPLC-glass vials. 50 µL of an eye homogenate sample (calibration samples, QCs or PD samples) were extracted with 50 µL of methanol and 1 µL caffeine (concentration 1 µg/mL in methanol) for 5 min at 800 rpm on an Eppendorf MixMate® vortex mixer. Then samples (eye homogenate) were spun down at 4,000 rpm for 40 min at 4 °C. Supernatants were transferred to 96well V-bottom plates (Greiner). HPLC conditions were as follows: column: Agilent Zorbax Eclipse Plus C18, 50x2.1 mm, 1.8 µm; temperature: 30 °C; injection volume: 5 µL; flow rate: 700 µL/min; solvent A: water + 0.1% formic acid; solvent B: acetonitrile + 0.1% formic acid; gradient: 99% A at 0 min and until 1 min, 99% – 0% A from 1.0 min to 2.2 min, 0% A until 4 min. Mass spectrometric conditions were as follows: Scan type: MRM, negative and positive mode; Q1 and Q3 masses for caffeine and **(*R*)-30** can be found in table S5 shown above. Peak areas of each sample and of the corresponding internal standard were analyzed using MultiQuant 3.0 software (AB Sciex). Peak areas of the respective sample were normalized to the internal standard peak area. The MS/MS pairs used for quantification are marked with a ‘Q’ in the table, the other MS/MS pairs for the respective compound were used for qualification. Peaks of PD samples were quantified using the calibration curve. The accuracy of the calibration curve was determined using QCs independently prepared on different days.

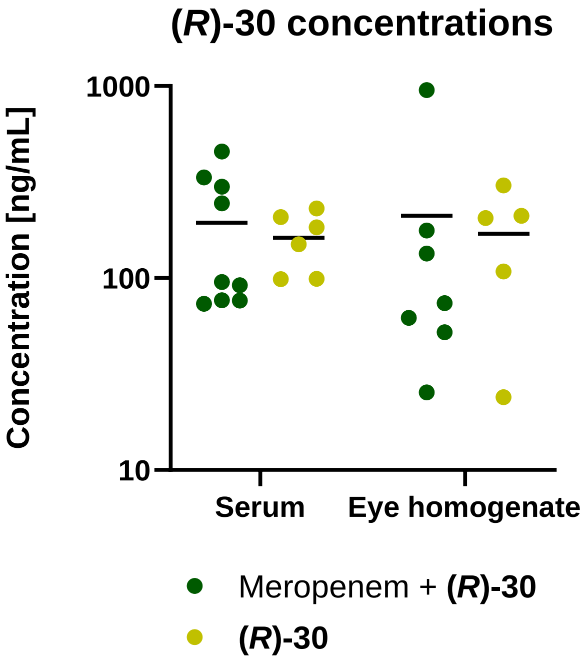

Figure S15: MS-based quantification of **(*R*)-30** levels in serum and eye homogenate. Concentrations were determined after single treatment (**(R)-30** alone) or after combination with Meropenem.

3.16 LabB-KC studies

To test whether KC might be a substrate for LasB, an ELISA was used to quantify KC levels after co-incubation with LasB. Briefly, 500 pg/ml of the recombinant murine KC standard (R&D part 840327) was incubated for 10 minutes in PBS supplemented with 1% BSA and 0.1% DMSO (vehicle control), LasB (100 nM), **(*R*)-30** (100 µM), or a combination of both. Detection of KC and absorbance measurements were performed as described above (3.11).

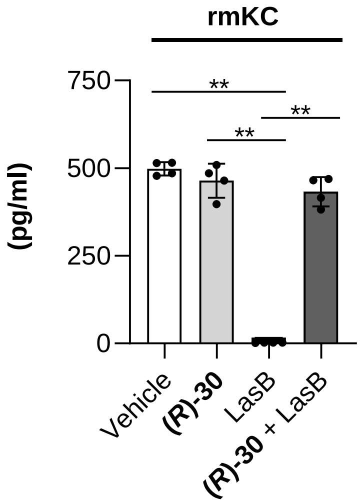

Figure S16: Impact of LasB and **(*R*)-30** on KC stability. 500 pg aliquots of recombinant murine KC (rmKC) were dissolved in PBS supplemented with 1% BSA and 0.1% DMSO and coincubated for 10 min with **(*R*)-30** (100 µM; light grey bar), LasB (100 nM; black bar) or a combination of both (dark grey bar), respectively. rmKC incubated in the solvent (PBS + 1% BSA + 0.1% DMSO) served as vehicle control. Integrity of rmKC was determined by ELISA using anti-mKC antibodies. Data shown represent the mean and SD of four replicates. **, $p < 0.01$ (Brown-Forsythe and Welch ANOVA test with Dunnett's T3 multiple comparison test).

3. Statistical Analysis

Statistics information for Figure 3										
Figure	Subfigure	Comparison	Symbol in Figure	Pre-processing	Data presentation	Sample size	Statistical method	Testing level	Post-hoc test	Software
3	A	Identical timepoints between groups: Comparison of the mean of each column with the mean of a control column (Sham-tretament, day 3)	°	no	Individual values and median	8	Ordinary one-way ANOVA	Alpha 0.05; Adjusted P value <0.05	Dunnett's multiple comparison test with a single pooled variance	GraphPad Prism (version 10.3.0)
3	A	Different timepoints within a group: Comparison of the mean of each column with the mean of a control column (day 1)	#	no	Individual values and median	8	RM one-way ANOVA with Geisser-Greenhouse correction	Alpha 0.05; Adjusted P value <0.05	Dunnett's multiple comparison test with individual variances computed for each comparison	GraphPad Prism (version 10.3.0)
3	B	Infected groups: Comparison of the mean of each column with the mean of a control column (Sham-treatment)	*	no	Individual values and median	8	Brown-Forsythe and Welch ANOVA test	Alpha 0.05; Adjusted P value <0.05	Dunnett's T3 multiple comparison test with individual variances computed for each comparison	GraphPad Prism (version 10.3.0)
3	B	All groups: Comparison of the mean of each column with the mean of a control column (Non-infected)	+	no	Individual values and median	8	Brown-Forsythe and Welch ANOVA test	Alpha 0.05; Adjusted P value <0.05	Dunnett's T3 multiple comparison test with individual variances computed for each comparison	GraphPad Prism (version 10.3.0)
3	C	Infected groups: Comparison of the mean of each column with the mean of a control column (Sham-treatment)	*	no	Individual values and median	8	Brown-Forsythe and Welch ANOVA test	Alpha 0.05; Adjusted P value <0.05	Dunnett's T3 multiple comparison test with individual variances computed for each comparison	GraphPad Prism (version 10.3.0)
3	C	All groups: Comparison of the mean of each column with the mean of a control column (Non-infected)	+	no	Individual values and median	8	Brown-Forsythe and Welch ANOVA test	Alpha 0.05; Adjusted P value <0.05	Dunnett's T3 multiple comparison test with individual variances computed for each comparison	GraphPad Prism (version 10.3.0)
3	D	Infected groups: Comparison of the mean of each column with the mean of a control column (Sham-treatment)	*	no	Individual values and median	6	Brown-Forsythe and Welch ANOVA test	Alpha 0.05; Adjusted P value <0.05	Dunnett's T3 multiple comparison test with individual variances computed for each comparison	GraphPad Prism (version 10.3.0)
3	D	All groups: Comparison of the mean of each column with the mean of a control column (Non-infected)	+	no	Individual values and median	6	Brown-Forsythe and Welch ANOVA test	Alpha 0.05; Adjusted P value <0.05	Dunnett's T3 multiple comparison test with individual variances computed for each comparison	GraphPad Prism (version 10.3.0)
3	E	Infected groups: Comparison of the mean of each column with the mean of a control column (Sham-treatment)	*	no	Individual values and median	6	Brown-Forsythe and Welch ANOVA test	Alpha 0.05; Adjusted P value <0.05	Dunnett's T3 multiple comparison test with individual variances computed for each comparison	GraphPad Prism (version 10.3.0)
3	E	All groups: Comparison of the mean of each column with the mean of a control column (Non-infected)	+	no	Individual values and median	6	Brown-Forsythe and Welch ANOVA test	Alpha 0.05; Adjusted P value <0.05	Dunnett's T3 multiple comparison test with individual variances computed for each comparison	GraphPad Prism (version 10.3.0)
3	F	Infected groups: Comparison of the mean of each column with the mean of a control column (sham-treatment)	*	no	Individual values and median	6	Brown-Forsythe and Welch ANOVA test	Alpha 0.05; Adjusted P value <0.05	Dunnett's T3 multiple comparison test with individual variances computed for each comparison	GraphPad Prism (version 10.3.0)
3	F	All groups: Comparison of the mean of each column with the mean of a control column (Non-infected)	+	no	Individual values and median	6	Brown-Forsythe and Welch ANOVA test	Alpha 0.05; Adjusted P value <0.05	Dunnett's T3 multiple comparison test with individual variances computed for each comparison	GraphPad Prism (version 10.3.0)
3	G	Infected groups: Comparison of the mean of each column with the mean of a control column (Sham-treatment)	*	no	Individual values and median	6	Brown-Forsythe and Welch ANOVA test	Alpha 0.05; Adjusted P value <0.05	Dunnett's T3 multiple comparison test with individual variances computed for each comparison	GraphPad Prism (version 10.3.0)
3	G	All groups: Comparison of the mean of each column with the mean of a control column (Non-infected)	+	no	Individual values and median	6	Brown-Forsythe and Welch ANOVA test	Alpha 0.05; Adjusted P value <0.05	Dunnett's T3 multiple comparison test with individual variances computed for each comparison	GraphPad Prism (version 10.3.0)

4. References

[1] E. Badiola, B. Fiser, E. Gómez-Bengoa, A. Mielgo, I. Olaizola, I. Urruzuno, J. M. García, J. M. Odriozola, J. Razkin, M. Oiarbide et al., *Journal of the American Chemical Society* **2014**, *136*, 17869.

[2] P. van der Veken, I. El Sayed, J. Joossens, C. Stevens, K. Augustyns, A. Haemers, *Synthesis* **2005**, *2005*, 634.

[3] A. M. Kany, A. Sikandar, J. Haupenthal, S. Yahiaoui, C. K. Maurer, E. Proschak, J. Köhnke, R. W. Hartmann, *ACS infectious diseases* **2018**, *4*, 988.

[4] D. Kolling, J. Haupenthal, A. K. H. Hirsch, J. Koehnke, *Chembiochem : a European journal of chemical biology* **2023**, *24*, e202300185.

[5] A. Burkhardt, T. Pakendorf, B. Reime, J. Meyer, P. Fischer, N. Stübe, S. Panneerselvam, O. Lorbeer, K. Stachnik, M. Warmer et al., *Eur. Phys. J. Plus* **2016**, *131*, 56.

[6] a) P. Evans, *Acta crystallographica. Section D, Biological crystallography* **2006**, *62*, 72; b) P. R. Evans, *Acta crystallographica. Section D, Biological crystallography* **2011**, *67*, 282; c) A. J. McCoy, R. W. Grosse-Kunstleve, P. D. Adams, M. D. Winn, L. C. Storoni, R. J. Read, *Journal of applied crystallography* **2007**, *40*, 658.

[7] a) A. M. Kany, A. Sikandar, S. Yahiaoui, J. Haupenthal, I. Walter, M. Empting, J. Köhnke, R. W. Hartmann, *ACS chemical biology* **2018**, *13*, 2449; b) M. M. Thayer, K. M. Flaherty, D. B. McKay, *Journal of Biological Chemistry* **1991**, *266*, 2864.

[8] a) P. Emsley, B. Lohkamp, W. G. Scott, K. Cowtan, *Acta crystallographica. Section D, Biological crystallography* **2010**, *66*, 486; b) P. D. Adams, P. V. Afonine, G. Bunkóczi, V. B. Chen, I. W. Davis, N. Echols, J. J. Headd, L.-W. Hung, G. J. Kapral, R. W. Grosse-Kunstleve et al., *Acta crystallographica. Section D, Biological crystallography* **2010**, *66*, 213.

[9] R. A. Laskowski, M. B. Swindells, *Journal of chemical information and modeling* **2011**, *51*, 2778.

[10] J. Konstantinović, A. M. Kany, A. Alhayek, A. S. Abdelsamie, A. Sikandar, K. Voos, Y. Yao, A. Andreas, R. Shafiei, B. Loretz et al., *ACS central science* **2023**, *9*, 2205.

[11] Y. Zhang, M. Huo, J. Zhou, S. Xie, *Computer methods and programs in biomedicine* **2010**, *99*, 306.

[12] S. Kiem, J. J. Schentag, *Antimicrobial agents and chemotherapy* **2008**, *52*, 24.

[13] Eurofins, "Safety Screen44 Panel", can be found under https://www.eurofinsdiscovery.com/catalog/safetyscreen44-panel-tw/PP241.

[14] J. Haupenthal, C. Baehr, S. Zeuzem, A. Piiper, *International journal of cancer* **2007**, *121*, 206.

[15] J. Maes, L. Verlooy, O. E. Buenafe, P. A. M. de Witte, C. V. Esguerra, A. D. Crawford, *PloS one* **2012**, *7*, e43850.

[16] J. Konstantinović, S. Yahiaoui, A. Alhayek, J. Haupenthal, E. Schönauer, A. Andreas, A. M. Kany, R. Müller, J. Koehnke, F. K. Berger et al., *Journal of medicinal chemistry* **2020**, *63*, 8359.

[17] H. Ceri, M. E. Olson, C. Stremick, R. R. Read, D. Morck, A. Buret, *Journal of clinical microbiology* **1999**, *37*, 1771.

[18] A.-P. Magiorakos, A. Srinivasan, R. B. Carey, Y. Carmeli, M. E. Falagas, C. G. Giske, S. Harbarth, J. F. Hindler, G. Kahlmeter, B. Olsson-Liljequist et al., *Clinical microbiology and infection : the official publication of the European Society of Clinical Microbiology and Infectious Diseases* **2012**, *18*, 268.

[19] C. N. Englisch, N. A. Wadood, L. Pätzold, A. Gallagher, G. Krasteva-Christ, S. L. Becker, M. Bischoff, *Annals of anatomy = Anatomischer Anzeiger : official organ of the Anatomische Gesellschaft* **2023**, *249*, 152099.
